# Genetic Structure and Demographic History Reveal Migration of the Diamondback Moth *Plutella xylostella* (Lepidoptera: Plutellidae) from the Southern to Northern Regions of China

**DOI:** 10.1371/journal.pone.0059654

**Published:** 2013-04-02

**Authors:** Shu-Jun Wei, Bao-Cai Shi, Ya-Jun Gong, Gui-Hua Jin, Xue-Xin Chen, Xiang-Feng Meng

**Affiliations:** 1 Institute of Plant and Environmental Protection, Beijing Academy of Agriculture and Forestry Sciences, Beijing, China; 2 State Key Laboratory of Rice Biology and Ministry of Agriculture Key Laboratory of Agricultural Entomology, Institute of Insect Sciences, Zhejiang University, Hangzhou, China; 3 Zhumadian Academy of Agricultural Sciences, Zhumadian, China; George Washington University, United States of America

## Abstract

The diamondback moth *Plutella xylostella* (Linnaeus) (Lepidoptera: Plutellidae) is one of the most destructive insect pests of cruciferous plants worldwide. Biological, ecological and genetic studies have indicated that this moth is migratory in many regions around the world. Although outbreaks of this pest occur annually in China and cause heavy damage, little is known concerning its migration. To better understand its migration pattern, we investigated the population genetic structure and demographic history of the diamondback moth by analyzing 27 geographical populations across China using four mitochondrial genes and nine microsatellite loci. The results showed that high haplotype diversity and low nucleotide diversity occurred in the diamondback moth populations, a finding that is typical for migratory species. No genetic differentiation among all populations and no correlation between genetic and geographical distance were found. However, pairwise analysis of the mitochondrial genes has indicated that populations from the southern region were more differentiated than those from the northern region. Gene flow analysis revealed that the effective number of migrants per generation into populations of the northern region is very high, whereas that into populations of the southern region is quite low. Neutrality testing, mismatch distribution and Bayesian Skyline Plot analyses based on mitochondrial genes all revealed that deviation from Hardy-Weinberg equilibrium and sudden expansion of the effective population size were present in populations from the northern region but not in those from the southern region. In conclusion, all our analyses strongly demonstrated that the diamondback moth migrates within China from the southern to northern regions with rare effective migration in the reverse direction. Our research provides a successful example of using population genetic approaches to resolve the seasonal migration of insects.

## Introduction

Many animals undergo seasonal migration to escape deteriorating habits, colonize new resources, avoid predation, competition and parasitism and benefit reproduction [1]–[5]. Understanding of the migration mechanisms is meaningful for species evolution, conservation biology and pest management [1], [6]–[8]. Compared with mammals and birds, migration pathways and destinations are poorly understood in insects [9]–[12]. It is difficult to apply conventional approaches–e.g., fluorescent marker dyes, radio-isotopes and radar monitoring [13]–to study the migration of insects, due to the very long distances over which they can fly, their small size and short lifespan and rapid aerial population dilution. Population genetic approaches have the potential to overcome many of those challenges [14], [15].

However, migration usually homogenizes the genetic differentiation among populations [6], [7], [16], leading to difficulty in deducing migration routes. Presently, most studies employing insect species, of which the migration patterns were well established, to test population genetic theories [6], [17], [18]. However, very few studies could reveal the seasonal migration pattern of insects using population genetic approaches [7]. Successful application of population genetic approaches to explore the pattern of seasonal migration requires a proper scale of the research area, time-dependent sampling, and polymorphic molecular markers, as well as the retrospective pattern of migration that the species displays.

The diamondback moth *Plutella xylostella* (Linnaeus) (Lepidoptera: Plutellidae) is one of the most destructive insect pests that affects cruciferous plants worldwide [19]. Combined management costs and yield losses due to the diamondback moth are estimated to be 4 to 5 billion United States dollars annually worldwide [20]. This moth is most likely of Mediterranean or South Africa origin [21], [22], but it is now recorded everywhere where crucifers are grown. Biological studies and field surveys have found that the diamondback moth cannot successfully overwinter in temperate regions where crucifers are not grown year-round, such as in western Canada [23] and northern Japan [24]. In the tropics and sub-tropics where crucifers are planted throughout the year, all life stages of this moth appear year-round [25], [26]. The diamondback moth was known to be a transoceanic migrant insect in Europe and American during its large-scale outbreaks in temperate regions [27]–[29].

Population genetic approaches have been previously used to reveal the migration of the diamondback moth. All studies have detected no genetic differentiation within the populations of Australia and New Zealand [30], [31], China [32], [33], the United States [34], Korea [35]–[37] and Hawaii [38]. However, none of these studies could directly reveal the migration pattern of the diamondback moth.

In China, where the climate ranges from temperate to subtropical to tropical, diamondback moth outbreaks occur yearly and cause heavy damage. Although Ma and Chen [39] speculated that the diamondback moth populations occurring in northern China were migrant from the southern region, the moth is still considered a local pest. Compared with previously researched regions, such as England, Australia and Korea, China offers an excellent opportunity for studying the regional migration of the diamondback moth because of its large size and range of climate types.

In the present study, we investigated the population genetic structure and demographic history of the diamondback moth across China by intense sampling, using both mitochondrial genes and microsatellite loci. The aim was to (1) apply genetic approaches to resolve the migration pattern of this insect without much background information and (2) reveal the migration pattern of the diamondback moth in China. If the migration of the diamondback moth is confirmed, the details of its life cycle and overwinter abilities in northern China should be reconsidered, and new controlling strategies based on forecasting systems could be developed for regional management of this insect pest. Additionally, the sampling and analysis methods used here would be a potential model to reveal the migration of other insects, such as the oriental armyworm *Mythimna separata* (Lepidoptera: Noctuidae) [40], the beet armyworm *Spodoptera exigua* (Lepidoptera: Noctuidae) [41], the meadow moth *Loxostege sticticalis* (Lepidoptera: Pyralidae) [42], the cotton bollworm *Helicoverpa armigera* (Lepidoptera: Noctuidae) [43] and the planthopper *Nilaparvata lugens* (Hemiptera: Delphacidae) [44].

## Results

### Genetic Variation

Five mitochondrial genes, *cox1*, *trnD*, *atp8*, *atp6* and *nad5*, from the 27 populations of the diamondback moth showed different genetic variation. We observed 205, 2, 12, 60 and 93 haplotypes in the *cox1*, *trnD*, *atp8*, *atp6* and *nad5* genes, respectively, in the 794 individuals (GenBank Accession numbers: KC154863-KC155245). The *cox1* alignment had the highest variable and parsimony-informative sites, haplotype (*h*) and nucleotide (π) diversity, followed by the *nad5*, *atp6* and *atp8* alignments, suggesting that these genes possess a large amount of genetic diversity. By contrast, *trnD* demonstrated low values of the diversity parameters (Table S1). Thus, sequences of the *trnD* gene were excluded from the subsequent analysis because of their short sequence length and rare genetic variation.

When the genes *cox1*, *atp6*, *atp8* and *nad5* were combined (2100 bp), 350 haplotypes were observed in 794 individuals. Among the 350 haplotypes, 246 were unique to one individual, 56 were unique to individuals from the same population, and 48 were shared by individuals from different populations. There were 272 (13.0%) variable characters, of which 156 (7.4%) were parsimony informative. The haplotype (*h*) and nucleotide (π) diversity were 0.9814 and 0.0024, respectively.

Based on the combined genes, all 27 populations showed high haplotype diversity and low nucleotide diversity. However, a relatively low value of the haplotype diversity was observed in the JSLY (0.5030), GXLZ (0.7330), JSNJ (0.7520) and FJQZ (0.7540) populations (Table 1).

**Table 1:** Parameters of genetic diversity and demographic analysis of 27 populations of the *Plutella xylostella.*

Population	*S*	*h*	π (%)	*P*	*D* (*p*)	*F_S_* (*p*)	τ (95% CI)	*P*_SSD_
HNSY	45	0.938	0.0033	6.8506	−1.4716 (0.0560)	−2.5492 (0.1670)	13.1934 (6.5724, 26.4603)	0.0254
HNDZ	35	0.984	0.0021	4.3126	−1.8698 (0.0140)	−19.6546 (0.0000)	53.5372 (24.4827, 123.8507)	0.0015
GDGZ	26	0.913	0.0013	2.6460	−2.1320 (0.0030)	−17.0327 (0.0000)	25.0555 (12.3531, 51.9361)	0.0016
GXLZ	14	0.733	0.0010	2.1310	−1.3247 (0.0750)	−1.0709 (0.3170)	3.2259 (1.4094, 7.0407)	0.0498
GXBS	53	0.970	0.0056	11.7724	−0.4487 (0.3570)	−6.2777 (0.0230)	43.3056 (20.3702, 96.3584)	0.0206
YNQJ	42	0.985	0.0035	7.4384	−1.3029 (0.0850)	1.1791 (0.7290)	5.9124 (2.6522, 12.8901)	0.0260
FJXM	21	0.821	0.0021	4.3425	−0.6331 (0.2890)	0.6288 (0.6340)	3.9824 (1.7973, 8.4785)	0.0520
FJLY	41	0.94	0.0026	5.3816	−1.7706 (0.0160)	−3.8907 (0.0780)	13.1934 (6.5724, 26.4603)	0.0168
FJQZ	7	0.754	0.0009	1.8943	0.2129 (0.6350)	−0.6193 (0.4080)	2.5582 (1.0729, 5.7651)	0.0615
JXNC	39	0.901	0.0024	4.9908	−1.8133 (0.0140)	−4.3585 (0.0450)	13.1934 (6.5724, 26.4603)	0.0063
ZJJH	17	0.932	0.0011	2.3248	−1.6531 (0.0350)	−7.9346 (0.0000)	11.0023 (5.2898, 22.8026)	0.0307
SHSX	28	0.970	0.0021	4.3586	−1.3776 (0.0700)	−11.1011 (0.0000)	25.0555 (12.3531, 51.9361)	0.0028
JSNT	27	0.920	0.0016	3.3264	−1.8344 (0.0130)	−10.4392 (0.0000)	18.0821 (9.0143, 36.6250)	0.0114
JSNJ	15	0.752	0.0009	1.8299	−1.7404 (0.0200)	−3.5768 (0.0220)	4.8393 (2.2417, 10.1055)	0.1870
JSYZ	22	0.883	0.0012	2.5402	−1.9088 (0.0120)	−9.6482 (0.0000)	13.1934 (6.5724, 26.4603)	0.0033
JSLY	10	0.503	0.0015	3.0368	0.6431 (0.7790)	1.7979 (0.8130)	1.9698 (0.7838, 4.6292)	0.3365
HNXY	38	0.966	0.0019	3.9862	−2.1461 (0.0050)	−12.0787 (0.0000)	25.0555 (12.3531, 51.9361)	0.0049
HNSQ	15	0.846	0.0011	2.3195	−1.3048 (0.0870)	−5.8628 (0.0010)	8.1735 (3.9844, 16.4981)	0.0029
SDQD	56	0.993	0.0028	5.9724	−2.1638 (0.0020)	−22.6331 (0.0000)	125.6752 (48.4094, 362.0404)	0.0036
SDYT	54	0.986	0.0025	5.2575	−2.2980 (0.0000)	−21.8471 (0.0000)	89.5436 (37.3400, 233.7848)	0.0405
QHXN	30	0.988	0.0020	4.1028	−1.8836 (0.0130)	−16.7654 (0.0000)	69.6777 (26.1922, 203.8010)	0.0009
HBCL	29	0.959	0.0017	3.6207	−1.8214 (0.0190)	−11.3118 (0.0000)	21.2377 (10.5459, 43.4357)	0.0029
HBBS	55	0.984	0.0028	5.8391	−2.1693 (0.0030)	−20.2598 (0.0000)	89.5436 (37.3400, 233.7848)	0.0042
BJYQ	61	0.984	0.0030	6.2368	−2.2372 (0.0030)	−19.2849 (0.0000)	89.5436 (37.3400, 233.7848)	0.0036
LNSY	54	0.977	0.0026	5.4736	−2.2387 (0.0000)	−16.3038 (0.0000)	53.5372 (24.4827, 123.8507)	0.0739
JLSP	51	0.973	0.0028	5.9678	−2.0014 (0.0110)	−15.1628 (0.0000)	53.5372 (24.4827, 123.8507)	0.0158
NMTL	54	0.986	0.0029	6.0828	−2.0715 (0.0070)	−22.3595 (0.0000)	125.6752 (48.4094, 362.0404)	0.0044

Number of polymorphic sites (*S*), haplotype diversity (*h*), nucleotide diversity (*π*) and the average number of pairwise differences (*P*) in the analyzed regions of the diamondback moth *Plutella xylostella* (L.); neutrality tests: Tajima’s D (*D*), Fu’s F statistics (*F_S_*) and expansion (coalescence) time under the sudden expansion assumption in mutation-generations (τ) with 95% confidence interval (CI). Significance values (*p*) of the parameters were evaluated using 1000 simulations; *P*_SSD_: *P* value for SSD (sum of squared deviations).

All microsatellite loci showed high levels of variation. The number of alleles ranged from 77 to 135, and the observed heterozygosity ranged from 0.17 to 0.90 in the 27 populations. There was no significant genotypic disequilibrium among the nine loci. Tests for Hardy-Weinberg equilibrium within populations and loci showed significant deviation (Table S2).

### Population Genetic Structure

The pairwise *F_ST_* difference based on mitochondrial genes showed significant differentiation in 521 of the 702 population pairs (Table 2). Most southern populations, such as FJXM, GDGZ, GXLZ and FJQZ, were significantly different from all other populations, as indicated by the *p* value of *F_ST_*. However, there were less differentiated population pairs in the northern region than in the southern regions. The microsatellite data showed significant pairwise differentiation in 13 of the 702 population pairs.

**Table 2:** Pairwise *F_ST_* values of 27 populations of the *Plutella xylostella* based on the combined mitochondrial genes of *cox1*, *atp8*, *atp6* and *nad5*.

Population	HNSY	HNDZ	GDGZ	GXLZ	GXBS	YNQJ	FJXM	FJLY	FJQZ	JXNC	ZJJH	SHSX	JSNT	JSNJ	JSYZ	JSLY	HNXY	HNSQ	SDQD	SDYT	QHXN	HBCL	HBBS	BJYQ	LNSY	JLSP	NMTL
HNSY	0																										
HNDZ	0.061	0																									
GDGZ	0.0564	0.1461	0																								
GXLZ	0.1448	0.1431	0.2794	0																							
GXBS	0.049	0.0852	0.1569	0.1675	0																						
YNQJ	0.0886	0.0569	0.1922	0.1964	0.0336	0																					
FJXM	0.0765	0.0972	0.2212	0.2203	0.1091	0.1452	0																				
FJLY	0.0447	0.0349	0.146	0.1296	0.0607	0.0647	0.0764	0																			
FJQZ	0.0806	0.0727	0.1982	0.2496	0.109	0.104	0.1158	0.0683	0																		
JXNC	0.0143	0.0543	0.0677	0.1604	0.052	0.0716	0.0913	0.033	0.033	0																	
ZJJH	0.0869	0.0305	0.2073	0.208	0.101	0.0697	0.1187	0.0555	0.0263	0.0416	0																
SHSX	0.0697	0.0099	0.1709	0.1725	0.0805	0.0657	0.0881	0.0525	0.0558	0.0594	0.0228	0															
JSNT	0.0785	0.0234	0.1856	0.1808	0.0926	0.0803	0.099	0.0363	0.0929	0.0699	0.052	0.0494	0														
JSNJ	0.1354	0.1619	0.305	0.3086	0.1463	0.1711	0.1986	0.1514	0.2739	0.166	0.2281	0.1947	0.1795	0													
JSYZ	0.0555	0.0204	0.1385	0.1718	0.1065	0.0884	0.1211	0.036	0.1019	0.0491	0.0637	0.0705	0.0372	0.1472	0												
JSLY	0.3125	0.3689	0.4687	0.5163	0.2867	0.3706	0.4215	0.3322	0.5136	0.3941	0.4899	0.3733	0.4444	0.539	0.4575	0											
HNXY	0.0531	0.0165	0.1417	0.1416	0.089	0.0754	0.0863	0.03	0.0643	0.0457	0.0268	0.0366	0.0316	0.0699	0.0158	0.3837	0										
HNSQ	0.1294	0.0304	0.2507	0.2566	0.1215	0.0574	0.1723	0.0999	0.1246	0.1075	0.0553	0.0532	0.0541	0.2555	0.0941	0.5001	0.061	0									
SDQD	0.0276	0.0045	0.1255	0.1249	0.0368	0.0361	0.0543	0.0058	0.0354	0.0215	0.023	0.0015	0.0191	0.1187	0.0228	0.3173	0.0118	0.053	0								
SDYT	0.0228	0.0017	0.1146	0.098	0.0483	0.0499	0.0649	0.0049	0.0393	0.0173	0.0218	0.017	0.0156	0.119	0.0116	0.3396	0.003	0.0513	−0.0141	0							
QHXN	0.0415	−0.0007	0.1478	0.148	0.082	0.0782	0.0707	0.0278	0.0516	0.0412	0.0332	0.0118	0.0376	0.1658	0.0301	0.3704	0.0088	0.0689	−0.0044	−0.0063	0						
HBCL	0.0549	0.0011	0.1486	0.1492	0.0842	0.0738	0.0933	0.0297	0.0549	0.0422	0.0229	0.0195	0.0273	0.1542	0.0265	0.3831	0.0055	0.0594	0.0046	−0.0005	0.0008	0					
HBBS	0.0372	0.0028	0.1273	0.1257	0.0414	0.0354	0.0652	0.0152	0.0434	0.0321	0.0226	0.0097	0.0076	0.1254	0.0264	0.3287	0.0115	0.0285	−0.0111	−0.01	0.0073	0.0012	0				
BJYQ	0.0349	−0.0011	0.1261	0.1202	0.0467	0.0338	0.0669	0.0156	0.0452	0.0265	0.02	0.0081	0.0215	0.1094	0.0253	0.3084	0.0023	0.0355	−0.0098	−0.0128	−0.0016	−0.0007	−0.0076	0			
LNSY	0.0332	0.001	0.1136	0.1252	0.0502	0.034	0.0858	0.0258	0.0434	0.0287	0.0182	0.0055	0.0285	0.1103	0.0238	0.3219	0.0026	0.0355	−0.0099	−0.0073	−0.0014	−0.0028	−0.0091	−0.0105	0		
JLSP	0.0325	0.0083	0.1299	0.1258	0.0388	0.0403	0.0691	0.0195	0.0423	0.0216	0.0238	0.0124	0.0305	0.1276	0.0307	0.3229	0.0132	0.052	−0.0096	−0.0089	0.0031	0.0067	−0.007	−0.0062	−0.0048	0	
NMTL	0.0447	−0.0047	0.1277	0.1332	0.0459	0.0153	0.0894	0.0242	0.0568	0.0347	0.0279	0.0119	0.0244	0.126	0.0211	0.3124	0.0186	0.0275	−0.0061	−0.0022	0.0052	0.0042	−0.0052	−0.0078	−0.0137	−0.0001	0

**Figure 1:**
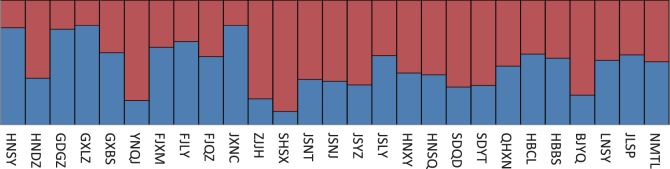
Membership coefficients of 27 *Plutella xylostella* populations divided into two inferred clusters obtained from the STRUCTURE program.

Next, we tested the division of the 27 populations into different geographical groups. The SAMOVA results based on mitochondrial genes showed that the *F_ST_*, *F_CT_* and *F_SC_* values decreased as the group number increased from 2 to 16 (Figure S1). When the populations were divided into 20 groups, the biggest group consisted of the BJYQ, HBBS, JLSP, LNSY, SDQD and SDYT populations from the northern region. The STRUCTURE program results based on microsatellite data divided the 794 individuals into two clusters. However, the two clusters were almost evenly distributed among all geographical populations and different regions (Figure 1). The global test of *F_ST_* in ARLEQUIN (*P* = 0.49447, 100000 Markov steps) based on microsatellite data also showed no differentiation among populations. Our analyses indicated that no geographical differentiation exists among populations in China.

**Figure 2:**
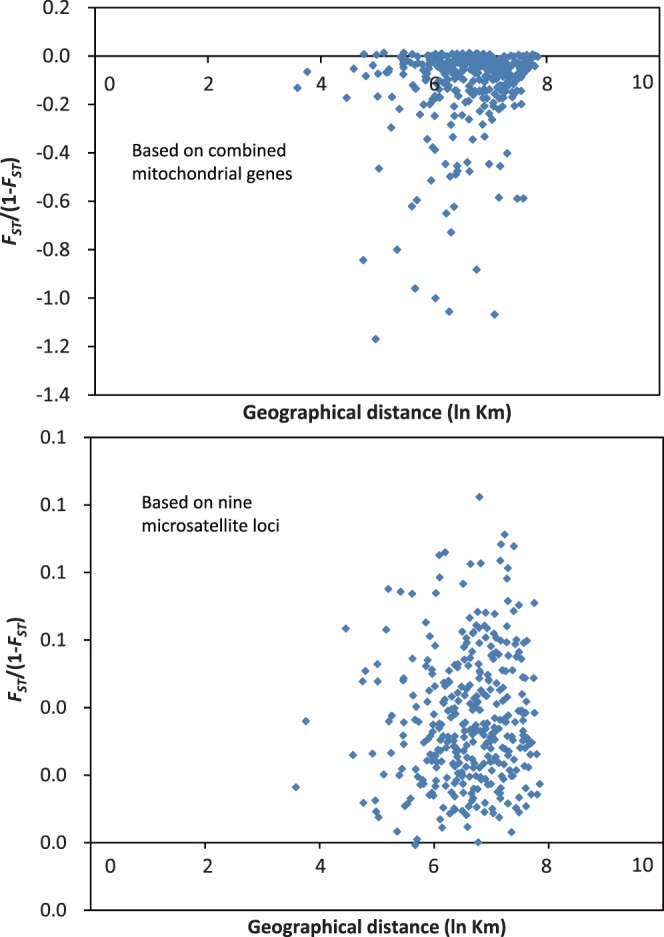
Scatter plots of genetic distance vs. geographical distance for pairwise population comparisons (for combined mitochondrial genes: r = 0.16075, *P* = 0.036496; for nine microsatellite loci: r = 0.0975, *P* = 0.1584; both analyses are calculated from 10000 randomizations).

Finally, the Mantel test results produced an r value of 0.16075 for combined mitochondrial genes (*P* = 0.036496) and 0.0975 for microsatellite data (*P* = 0.1584) (Figure 2), indicating that no correlation was found between genetic and geographical distance among the populations of the diamondback moth in China.

### Haplotype Phylogeny

**Figure 3:**
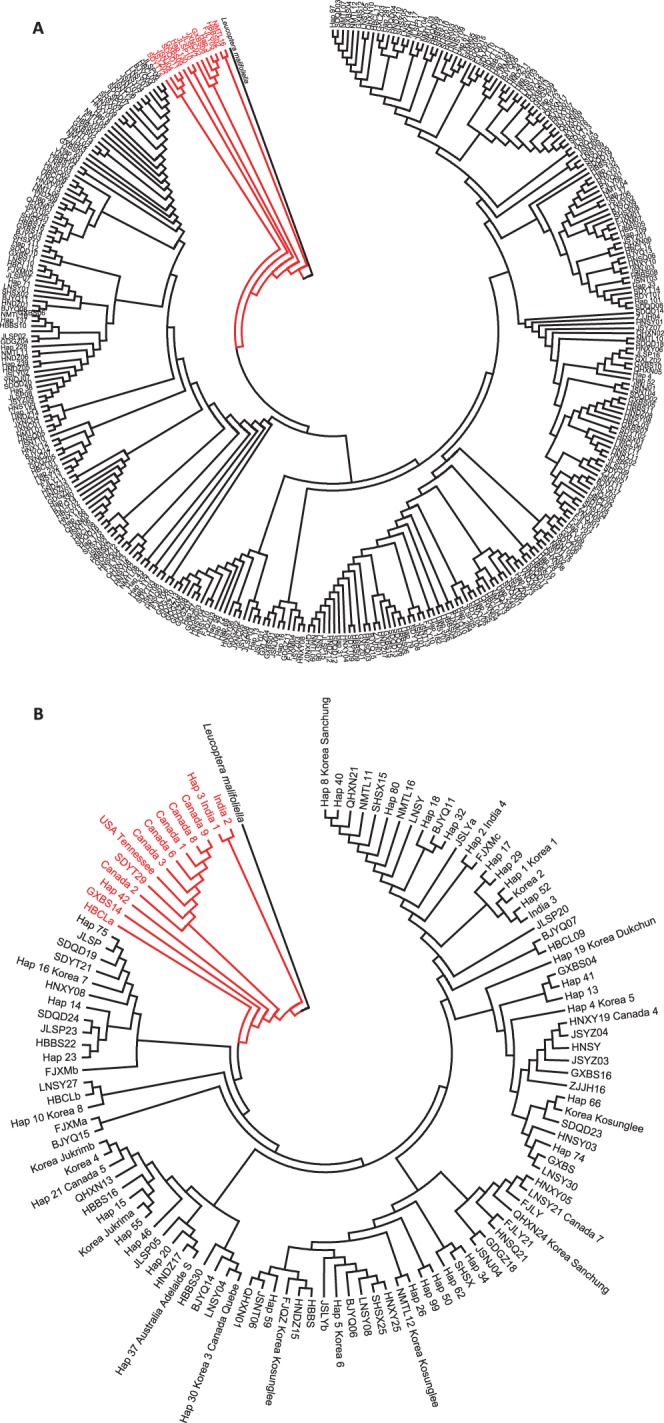
Neighbor-Joining phylogenetic trees of the haplotypes of the diamondback moth from China based on the combined genes of *cox1*, *atp8*, *atp6* and *nad5* (A), and globally based on the *cox1* gene (B). When the haplotype was unique to one individual, the population name with a number was used as the haplotype name; When the haplotype was unique to individuals from the same population, the population name with a letter was used as the haplotype name; When the haplotype was shared by different populations from China, the “Hap” with a number was used as the haplotype name; When the haplotype was found in other countries, the country name was added/used in the haplotype name.

When the 350 haplotypes of the combined genes from China were analyzed, two obvious mtDNA groups were present in the BI and NJ trees. One group contained mostly haplotypes, whereas the other group contained 13 haplotypes that were distributed from the southern to northern regions of China (Figure 3A). When we compared the haplotypes of the *cox1* gene (372 bp) worldwide, 10 of the 15 haplotypes from Korea, one from Australia and one of seven from Canada were identical to haplotypes from China. The phylogenetic analysis also divided the haplotypes worldwide into two groups (Figure 3B). One group contained mostly haplotypes from China and all haplotypes from Korea, whereas the other one contained one haplotype from China and India, one from China, one from India, one from USA, and six from Canada.

### Haplotype Network

**Figure 4:**
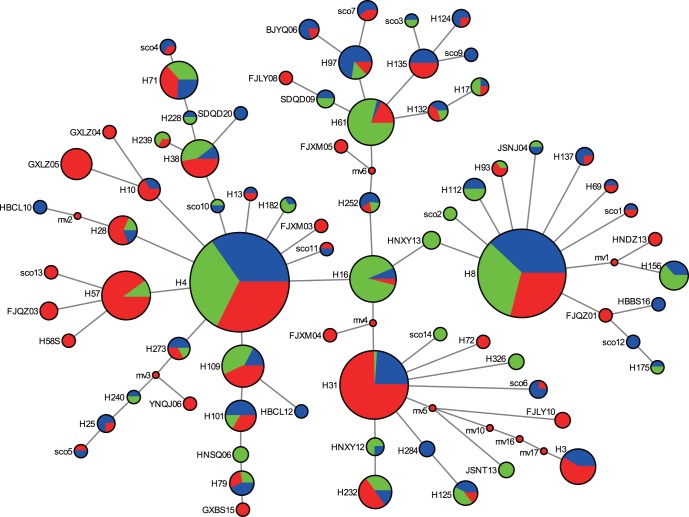
The haplotype network of the combined mitochondrial genes of *cox1*, *atp8*, *atp6* and *nad5*. The area of a circle is proportional to the number of observed individuals. Colors within the nodes: red, southern China; green, middle regions of China; blue, northern China.

The haplotype network tree of combined genes and *cox1* formed four major groups (Figure 4), whereas that of *atp6* and *nad5* obviously displayed a star-like pattern with the most common haplotypes in the star’s center (Figure S2, S3, S4). All the dominant haplotypes were evenly distributed in the southern, middle and northern regions of China, except the third dominant haplotype in the network of combined genes and *cox1*, most of which were mainly observed in the southern region of China (Table S4). In addition, most of the terminal haplotypes were from southern China.

### Gene Flow

The analysis between each pair of the 27 populations showed that high gene flow was present. Unidirectional estimates of *M* ranged from 331.7 (from QHXN to FJQZ) to 662.8 (from FJLY to LNSY) (Table S3). When the *M* values were translated into effective migrants per generation (*N_e_m*), the numbers of migrants into northern populations were high, ranging from 9.8 to 36.6 for each population. By contrast, the numbers of effective migrants into southern populations were lower than 4.8 for each population, except HNDZ and GXBS (Table 3). When the three geographical groups were analyzed, the migrants from the southern and middle regions to the northern regions were high, with values of 76.7 and 92.1, respectively, whereas the migrants from the northern to southern and middle regions were low, with values of 21.7 and 6.1, respectively.

**Table 3:** Numbers of effective migrants per generation (*N_e_m*) in the 27 *Plutella xylostella* populations based on the combined genes of *cox1*, *atp8*, *atp6* and *nad5.*

Population, *i*	θ*i*	HNSY →*i*	HNDZ →*i*	GDGZ →*i*	GXLZ →*i*	GXBS →*i*	YNQJ →*i*	FJXM →*i*	FJLY →*i*	FJQZ →*i*	JXNC →*i*	ZJJH →*i*	SHSX →*i*	JSNT →*i*	JSNJ →*i*	JSYZ →*i*	JSLY →*i*	HNXY →*i*	HNSQ →*i*	SDQD →*i*	SDYT →*i*	QHXN →*i*	HBCL →*i*	HBBS →*i*	BJYQ →*i*	LNSY →*i*	JLSP →*i*	NMTL →*i*	Total *i*
HNSY	0.00210	–	1.1	1.2	0.9	1.1	1.0	1.1	1.0	1.3	0.9	1.2	1.0	0.9	0.9	1.1	1.2	1.0	0.9	1.0	1.0	1.2	1.1	1.1	0.8	1.1	1.0	0.9	26.8
HNDZ	0.03140	16.4	–	14.5	17.0	14.7	14.2	15.8	13.9	16.7	15.5	13.5	12.8	13.0	16.5	15.0	16.1	13.3	15.7	13.0	13.0	15.7	13.8	15.6	17.0	18.6	17.4	14.1	393.0
GDGZ	0.00183	0.9	0.7	–	0.9	0.8	0.7	0.8	0.8	0.9	1.0	0.8	0.8	0.7	0.8	0.8	0.9	0.8	0.8	0.9	0.8	0.8	0.8	0.8	0.8	0.8	0.9	0.8	21.3
GXLZ	0.00049	0.2	0.2	0.2	–	0.2	0.2	0.2	0.2	0.3	0.2	0.2	0.2	0.2	0.2	0.2	0.3	0.2	0.2	0.2	0.2	0.2	0.2	0.2	0.2	0.2	0.2	0.2	5.6
GXBS	0.03328	15.0	16.2	15.7	19.5	–	18.9	15.6	16.1	16.4	15.3	15.0	15.9	14.1	16.3	15.7	14.9	18.7	14.0	14.1	17.0	16.8	16.5	17.7	16.9	16.3	16.9	17.0	422.4
YNQJ	0.00195	0.9	0.9	0.9	1.0	1.0	–	1.0	0.9	1.1	0.8	1.0	0.9	1.1	0.8	1.0	1.1	0.9	0.8	1.0	1.1	1.1	0.9	1.0	0.9	0.9	0.9	0.9	24.8
FJXM	0.00067	0.3	0.3	0.3	0.3	0.3	0.3	–	0.3	0.4	0.3	0.3	0.3	0.3	0.3	0.3	0.3	0.3	0.3	0.3	0.4	0.3	0.3	0.3	0.3	0.3	0.3	0.3	8.0
FJLY	0.00215	1.1	1.0	1.0	1.0	1.2	1.1	1.0	–	1.3	1.2	1.1	1.0	1.1	0.8	1.1	1.2	0.9	1.0	1.1	1.0	1.1	1.1	1.1	1.0	1.0	0.9	1.1	27.4
FJQZ	0.00041	0.2	0.2	0.2	0.2	0.2	0.2	0.2	0.2	–	0.2	0.2	0.2	0.2	0.2	0.2	0.2	0.1	0.2	0.2	0.2	0.1	0.2	0.2	0.2	0.2	0.2	0.2	4.6
JXNC	0.00358	1.7	1.6	2.3	1.8	2.0	1.8	1.8	1.8	2.2	–	1.5	1.9	1.6	1.8	1.6	1.9	1.6	1.7	1.7	1.8	1.8	2.0	1.7	2.1	1.8	1.5	1.9	46.7
ZJJH	0.00861	4.6	4.4	3.4	4.6	4.7	3.6	5.1	4.0	4.9	4.6	–	5.3	3.8	3.9	3.8	4.7	4.1	4.2	3.9	4.1	4.3	4.4	3.7	4.8	3.9	4.4	4.0	111.2
SHSX	0.00767	3.9	3.6	3.8	3.5	3.9	3.5	3.5	4.1	4.2	3.7	4.6	–	3.6	4.1	3.4	3.6	3.8	3.4	3.9	4.0	3.9	3.7	3.9	3.5	3.9	3.8	3.6	98.5
JSNT	0.00377	1.9	1.6	1.7	2.0	1.5	2.0	1.9	2.0	2.3	1.8	1.7	1.9	–	1.7	1.7	1.9	1.7	1.8	1.8	1.7	2.0	1.9	1.7	1.7	1.6	2.1	1.9	47.6
JSNJ	0.00072	0.3	0.3	0.3	0.4	0.4	0.3	0.3	0.3	0.4	0.4	0.3	0.3	0.4	–	0.3	0.4	0.4	0.3	0.4	0.3	0.4	0.3	0.4	0.3	0.3	0.4	0.4	9.0
JSYZ	0.01236	6.1	6.3	7.3	6.4	5.8	5.9	5.7	5.8	6.1	6.2	5.1	5.9	5.4	7.6	–	7.6	5.5	5.6	6.8	6.6	6.0	6.0	5.6	5.6	6.0	5.1	5.8	157.9
JSLY	0.00025	0.1	0.1	0.1	0.1	0.1	0.1	0.1	0.1	0.1	0.1	0.1	0.1	0.1	0.1	0.1	–	0.1	0.1	0.1	0.1	0.1	0.1	0.1	0.1	0.1	0.1	0.1	2.8
HNXY	0.01196	5.4	5.4	5.3	5.3	6.0	5.2	5.8	7.3	6.0	5.3	5.1	6.1	6.3	7.8	5.8	6.2	–	4.8	5.2	6.2	6.3	6.7	6.7	7.2	6.4	6.4	7.0	157.3
HNSQ	0.00239	1.1	1.1	1.0	1.2	1.1	1.1	1.2	0.9	1.4	1.2	1.1	1.1	1.2	1.0	1.1	1.3	1.2	–	1.0	1.3	1.1	1.1	1.1	1.2	1.3	1.2	1.1	29.8
SDQD	0.06237	31.4	34.2	27.1	27.8	32.8	29.8	30.0	30.8	34.9	28.8	32.9	31.7	32.9	28.2	29.3	29.6	32.9	29.6	–	32.8	31.4	34.9	27.1	35.3	37.0	31.4	31.3	815.7
SDYT	0.06377	33.1	36.2	38.7	34.3	37.0	35.1	31.7	30.5	37.3	29.1	35.7	27.2	28.7	32.7	29.7	30.7	33.4	29.8	36.6	–	30.8	32.4	33.6	35.9	30.0	29.7	30.1	849.9
QHXN	0.05435	24.6	25.4	28.5	29.0	29.8	24.7	29.5	21.9	31.1	32.6	29.0	24.8	27.9	26.4	27.0	28.6	23.0	30.7	32.4	28.6	–	23.7	23.1	26.4	33.7	29.1	23.8	715.3
HBCL	0.01117	5.9	5.6	5.4	5.6	5.7	4.5	4.9	5.6	5.9	5.2	5.0	5.1	4.4	5.2	6.1	6.7	6.3	5.2	6.1	6.5	5.5	–	4.8	5.3	5.4	6.3	6.0	144.4
HBBS	0.06404	29.9	35.7	30.7	30.3	32.2	25.3	31.0	26.2	32.5	34.3	27.2	29.8	30.8	28.5	30.8	36.6	30.9	26.0	36.0	34.3	27.6	32.4	–	32.2	34.1	26.9	36.5	808.5
BJYQ	0.06248	33.9	26.3	23.2	32.1	29.9	36.1	28.8	27.9	32.6	29.6	25.7	27.2	31.7	35.7	25.1	29.5	30.9	30.9	29.7	30.1	31.1	34.6	30.3	–	35.1	30.3	29.8	788.3
LNSY	0.05803	24.3	25.0	23.0	27.9	28.2	30.1	22.6	24.9	38.5	34.2	31.2	26.3	34.4	31.3	27.3	32.1	34.1	29.2	31.9	27.3	27.5	25.6	31.4	26.1	–	31.7	28.8	754.6
JLSP	0.02269	11.1	11.8	11.0	11.1	11.1	11.5	10.4	9.6	12.4	11.3	11.7	9.2	11.9	9.9	10.7	11.3	10.7	11.2	12.2	11.6	10.8	11.5	12.9	9.8	10.3	–	11.0	287.8
NMTL	0.06468	34.7	38.1	28.2	33.2	28.3	28.9	27.1	31.1	32.4	28.1	33.2	31.8	26.8	28.7	37.8	39.0	34.7	33.8	27.3	25.9	32.8	30.4	32.7	36.1	33.3	30.0	–	824.6

### Haplotype Distribution

**Figure 5:**
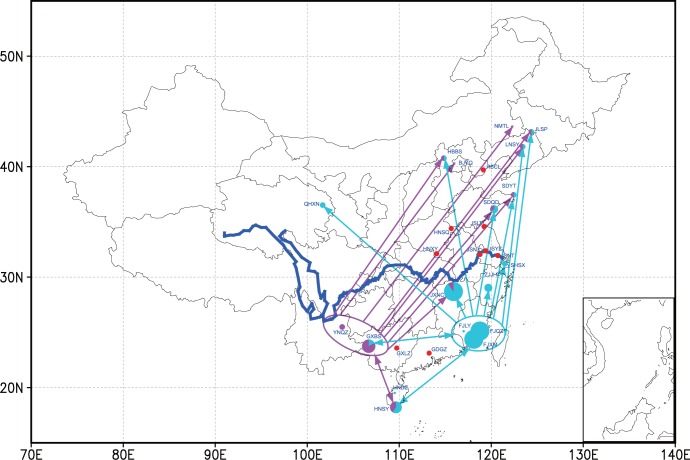
Distributions of two dominant haplotypes of the combined mitochondrial genes of *cox1*, *atp8*, *atp6* and *nad5*. Detailed locations and codes of the populations are shown in Table 4. The area of the circle indicates the number of the haplotypes Hap3 (blue-green) and Hap31 (pink). The red circle indicates the other collection locations without the haplotypes of Hap3 and Hap 31. The blue line on the map indicates the Yangtze River. Populations north of the Yangtze river could not overwinter. The arrows indicate the possible migration routes of the two haplotypes from the southern to northern regions of China.

Several haplotypes were dominant and evenly distributed among the different regions of China (Tables S4), whereas most haplotypes were unique to individuals and populations. Both of the above haplotype types were uninformative for directly revealing the migration route. Here, we compared the distribution of two moderately dominant haplotypes (Hap31 and Hap3). In the southern region, the Hap3 haplotype was found in populations from the Fujian and Hainan provinces, whereas the Hap31 haplotype was found in the GXBS, YNQJ and HNSY populations. Furthermore, both haplotypes were found in most populations in the middle and northern regions (Figure 5).

### Demographic History

Neutrality tests were conducted using Tajima’s D and Fu’s F statistics. When the 27 populations were considered as one group, Tajima’s D was significantly negative for the genes *cox1*, *atp8*, *atp6* and *nad5*. We then calculated Tajima’s D for each of the 27 populations using the combined genes and found that ‘it was not significantly negative in all populations from southern China except the populations HNDZ (*p* = 0.0140), GDGZ (*p* = 0.003) and FJLY (*p* = 0.0160), whereas Tajima’s D was statistically negative in all populations from northern China. Similar tendencies were found using Fu’s F statistics (Table 1).

Mismatch distributions for each population obtained from the combined genes were unimodal in all populations from the northern regions, most populations from the middle regions (exceptions are JSNJ and JSLY), and four populations from the southern regions (HNDZ, HNSY, GDGZ and FJLY), indicating that a sudden demographic expansion occurred in these populations (Figure S5).

The effective population size estimated for each population showed that an obvious expansion occurred in populations from the northern regions (except HBCL and QHXN) and GXBS from the southern regions, whereas a decrease or no variation of effective population size was observed in populations from the middle and southern regions (Figure S6).

## Discussion

### Recent Introduction and Adaption of the Diamondback Moth in China

In the diamondback moth, low mitochondrial DNA diversity among Australian populations were found based on a 257-bp *cox1* sequence [30]. When a long *cox1* gene sequence was used for analysis of Chinese and Korea populations, the haplotype diversity became high [32], [37]. The gene sequences used in our study represent all three types of protein-coding genes (cytochrome c oxidase, ATP synthase F0 and NADH dehydrogenase subunit) and the 22 tRNA genes. These genes are located evenly throughout the circular molecules of the mitochondrial genome, which are powerful for studying the population genetic structure of the diamondback moth, as previously demonstrated in other insects [14], [45].

Our study revealed high haplotype diversity (*h*) with low nucleotide diversity (π) in the populations of the diamondback moth from China. A similar situation was previously found in many species, with a high migration rate and small effective population size [46], [47]. Low nucleotide diversity but high haplotype diversity might be indicative of genetic bottleneck events. The diamondback moth is believed to have originated in the Mediterranean area, the source of some of our most important crucifers [21], [48], or in southern Africa, with a large number of indigenous plants from the Brassicaceae, and is rich in parasitoids [22]. This pest became worldwide in the recent 156 years [49]. For example, in Taiwan, China, the diamondback moth was first reported as a pest over 100 years ago [50]. This relatively recent introduction of the diamondback moth followed by its population expansion might be the cause of the low nucleotide diversity observed.

A plausible explanation for the high overall degree of haplotype diversity identified might be that these haplotypes are important for adaptation to different local habitats [51]. The diamondback moth has developed resistance to various insecticides in the field [52]. Variation in pesticide resistance was found between populations geographically nearby [38]. Genetic difference was found between pesticide-resistant and susceptible strains [53]–[55] and between populations at different temperatures and altitudes [56]. In the tropics and subtropics, the continuous cultivation and short life cycle of the diamondback moth can result in more than 25 generations a year being exposed to synthetic insecticides routinely used by growers. This high level of selection pressure of pesticide and local climate might be the key factors leading to high haplotype diversity. This is congruent with the network analysis, in which the terminal haplotypes usually originated from the southern region. Additionally, demographic analysis showed that the populations from the southern region were in selective neutrality with a stable effective population size and no sudden demographic expansion. These analyses indicated that the diamondback moth had reached stability in gene frequency in southern China.

### Evidence for Migration of the Diamondback Moth in China

Early studies presumed that the diamondback moth hibernates in host-plant debris through the winter in temperate regions where crucifers are not grown year-round [57], [58]. However, in none of these studies were insects collected during the coldest months and brought out of hibernation [19]. Presently, many studies have demonstrated that the diamondback moth cannot overwinter in temperate regions [59], [60]. In China, Ma and Chen [39] first proved that the diamondback moth could not overwinter in the northeast region using indoor and outdoor data during the winter and consequently proposed the hypothesis that this moth migrates with the south-western airflow into the north-eastern area of China based on their long distance flight ability, aerial-trapped samples, and sudden increase of trapping number in the spring. Chen and Liu [61] domestrated that the lethal time for 90% (LT90) population mortality of the diamondback moth exposed to −10°C was shorter than 69 hours. Those biological and ecological studies indicated that the diamondbakc moth could not overwinter in the cold regions of China.

In the present study, we did not find geographical differentiation among the 27 populations of the diamondback moth in China based on both mitochondrial sequences and microsatellite data. A consequent Mantel test revealed no isolation by distance, as in Australia and New Zealand revealed by microsatellite markers [7]. The network analysis showed that all the dominant haplotypes were evenly distributed in the southern, middle and northern regions of China. These genetic features are evidence for migration, as demonstrated in other migratory species [6], [15], [31], [46], [62].

### The Migration Route within China

In temperate regions of the northern hemisphere, migrations of moths are often directed northward in spring and summer and southward in autumn [5], [63]–[65]. Our analysis of gene flow indicates that the diamondback moth migrated from the southern to northern regions of China. However, rare effective migration was detected in the reverse direction. The unidirectional migration with seldom return of the progeny has been found in other insect species [66]. Our finding is congruent with the yearly occurrence of the diamondback moth in China. First, in the northern region, the population of diamondback moth increases quickly starting in May as the temperature increases [67], during which time the first generations are found in the southern region in field [68]. Second, many insects employ the wind to facilitate their long-distance migration because of their small body size [64], [69]. For example, Lokki et al. [70] reported mass migration of the diamondback moth to Spitsbergen from South Finland and Finnish Lapland, carried by a strong south-southeastern storm. Light trapping of moths on the northern coast of Tasmania has also revealed peaks in abundance of the diamondback moth in spring and summer, sometimes associated with storm fronts [31]. The East Asian subtropical monsoon that occurs from the southeastern and southwestern to northern directions in the spring and summer facilitates migration of this insect in China from the southern to northern regions. Third, the outbreak of this pest in the northern region usually occurs in June and July. However, during mid-summer and autumn, the diamondback moth causes relative less damage with a low population quantity likely caused by the increase of natural enemies [68], unsuitable high temperature and frequent rainfall during the summer [71]. The low population density might lead to decreased effective migration from the northern region back to the southern region.

The detailed migration route of the diamondback moth is complicated. The effective migrants per generation from multiple populations into each northern population were high, indicating that northern populations resulted from an admixture of multiple southern populations. The effective migrants per generation into two southern populations–i.e., HNDZ and GXBS–were relatively higher than those into other southern populations, indicating migration amongst populations in the southern region. However, the high value of effective migrants per generation among northern populations might be caused by the shared sources from the southern region. When we marked the two moderately distributed haplotypes on a map, the migration route was obviously congruent with the predicted pattern based on statistical results (Figure 5).

### Origin of the Diamondback Moth in China

There are two groups of the diamondback moth in China and worldwide (Figure 3). The haplotypes from the major groups are dominant and widely distributed in China. The phylogenetic tree inferred from all haplotypes of China could represent the proportion of the two types of haplotype. However, the worldwide phylogenetic tree just represents the division of the haplotypes worldwide. Frequent migration between China and Korea is reasonable for their near distance, as revealed in rice planthoppers [72]. It is congruent with our results that all the haplotypes found in Korea and Australia were classified into the major group of China. Additionally, the same haplotypes of the diamondback moth between Australia and Korea have been reported [30]. Within the phylogenetic tree, although individuals from the North America and those from Asia and Australia formed two different groups, the haplotypes from the North American group could be found in China from the southern to northern regions. Nevertheless, the Chinese and global structures of the haplotypes might indicate that most of the diamondback moths presently occurred in China might be from the southern regions of China and/or Australia, with minor individuals introduced from other regions, such as Canada and American. It is also possible that the two groups of the diamondback moth were introduced into China and occurred afterward in the mixture.

### Implications for Pest Management

Our data show that the diamondback moth migrates from southern to northern within China with rare effective migration in reverse direction. This migration has many implications for the management of this pest. Although this species develops resistance to insecticides rapidly, presently, applications of synthetic insecticides remain overwhelmingly the most common control strategy [73]. Therefore, it should be considered that insecticide resistance alleles arising in the southern region might spread quickly into the northern region via migration.

Generation predictions in the northern region should not merely depend on the development rate and the accumulated temperature; instead, it should also take into consideration the timing of the arrival of new migrants from the southern region. Aerial detection of the adult dynamics should be incorporated into the forecast of the diamondback moth.

### Conclusions

In conclusion, we investigated the population genetic structure and demographic history of the diamondback moth in China. Both the mitochondrial sequences and microsatellite data showed no differentiation or isolation by distance in the 27 geographical populations, indicating migration among the diamondback moth populations in China. Pairwise differentiation based on mitochondrial sequences, gene flow, and demographic history strongly showed that the migration pattern of this moth is in the northward direction with rare effective reverse re-migration. Phylogenetic analysis of the mitochondrial gene haplotypes indicated that one dominant and one minor group were present in China in the mixture. Our research provides a successful example of a method for understanding the seasonal migration of insects.

## Materials and Methods

### Ethics Statement

The diamondback moth is a pest insect of vegetables. The study of this pest is welcomed by farmers because understanding the behavior this moth may be helpful to protect their vegetables from pest damage. Thus, no specific permits were required for the described field studies. Additionally, the field studies did not involve endangered or protected species.

### Insect Sampling

In China, biological studies revealed that the diamondback moth could not overwinter in regions north of the Yangtze River [59], [74]. In our study, 794 diamondback moth larvae were collected from cabbage fields at 27 locations. The sampled populations included nine collected from the south of the Yangtze River, nine from the north of the Yangtze River, and nine around the east of the Yangtze River, covering all representative regions of China (Table 4 and Figure 5). Five populations were collected in the year 2010, and 22 populations were collected in 2011. Most populations from southern China were collected in the early part of 2011 when the population density of the diamondback moth was low in the field, whereas most populations from northern China were collected immediately when the population reached a high density. All specimens were preserved in absolute ethanol and stored at −80°C prior to DNA extraction.

**Table 4:** Collection details of the *Plutella xylostella* populations used in this study.

Region	Code	Collection location	Latitude(N)	Longitude(E)	Elevation (Feet)	Collection date	Number of individual
South	HNSY	Hainan Province, Sanya	18°15′10/54″	109°30′42/92″	19	Mar-11	30
	HNDZ	Hainan Province, Danzhou	19°31′16/05″	109°34′50/92″	471	Apr-11	30
	GDGZ	Guangdong Province, Guangzhou	23°07′44/99″	113°15′51/97″	17	Nov-10	30
	GXLZ	Guangxi Province, Liuzhou	23°35′52/26″	109°44′22/85″	862	Mar-11	30
	GXBS	Guangxi Province, Baise	23°54′08/54″	106°41′40/73″	564	Jan-11	30
	YNQJ	Yunnan Province, Qujing	25°29′24/00″	103°47′46/11″	6158	Aug-11	24
	FJXM	Fujian Province, Xiamen	24°28′47/41″	118°05′21/91″	50	Feb-11	30
	FJLY	Fujian Province, Longyan	25°06′02/28″	117°02′02/51″	1145	Mar-11	30
	FJQZ	Fujian Province, Quanzhou	24°54′27.27′′	118°35′12.52′′	43	Feb-11	30
Middle	JXNC	Jiangxi Province, Nanchang	28°40′59/38″	115°51′29/12″	48	Dec-10	30
	ZJJH	Zhejiang Province, Jinhua	29°04′44/99″	119°38′50/72″	147	Jun-11	27
	SHSX	Shanghai, Shouxian	31°13′49/41″	121°28′25/33″	21	Apr-11	30
	JSNT	Jiangsu Province, Nantong	31°57′56/21″	120°43′15/36″	6	Jun-11	30
	JSNJ	Jiangsu Province, Nanjing	32°03′36/59″	118°47′57/23″	103	Jun-11	30
	JSYZ	Jiangsu Province, Yangzhou	32°23′39/16″	119°24′46/68″	25	Oct-10	30
	JSLY	Jiangsu Province, Lianyungang	34°35′49/48″	119°13′15/17″	12	Jun-11	30
	HNXY	Henan Province, Xinyang	32°07′23/64″	114°04′07/94″	277	May-11	30
	HNSQ	Henan Province, Shangqiu	34°24′51/06″	115°39′23/02″	167	Apr-11	30
North	SDQD	Shangdong Province, Qingdao	36°04′02/32″	120°22′57/27″	173	Jun-11	30
	SDYT	Shandong Province, Yantai	37°27′49/52″	122°26′52/23″	30	Jun-11	30
	QHXN	Qinghai Province, Xining	36°31′00/54″	101°40′49/81″	8256	Jul-10	23
	HBCL	Hebei Province, Changli	39°42′46/14″	119°09′45/73″	77	May-11	30
	HBBS	Hebei Province, Bashang	40°46′03/27″	114°53′09/42″	2371	Aug-10	30
	BJYQ	Beijing Province, Yanqing	40°27′23/97″	115°58′29/92″	1677	Jul-11	30
	LNSY	Liaoning Province, Shenyang	41°48′20/59″	123°25′53/29″	155	Aug-11	30
	JLSP	Jilin Province, Siping	43°10′00/97″	124°21′01/27″	537	Aug-11	30
	NMTL	Neimenggu Province, Tongliao	43°37′10/00″	122°15′56/67′′	594	Aug-11	30

### DNA Extraction and Sequencing

Total genomic DNA was extracted from one abdominal segment of individual larva using the DNeasy Blood and Tissue Kit (Qiagen, Germany). The gut was removed prior to DNA extraction.

To choose the gene region for the population genetic study and design the primers for amplification, we sequenced the complete mitochondrial genome of the diamondback moth as the reference sequence (GenBank accession No. JF911819). Three segments from mitochondrial genomes, including a partial sequence of *cox1* and *nad5* and full-length sequences of *atp8*, *atp6* and *trnD*, were used as molecular markers for the current study according to the types of the mitochondrial protein-coding genes and previous studies [45]. Primers were designed using Primer Premier version 5 [75] according to the reference mitochondrial genome (Table S5). Polymerase chain reaction (PCR) was conducted using the Mastercycler pro system (Eppendorf, Germany) under the following conditions: an initial denaturation for 2 min at 94°C, followed by 35 cycles of 10 s at 96°C, 15 s at 48°C for the region of *cox1*-*cox2* and 54°C for the regions of *atp8*-*atp6* and *nad5*, and 1 min at 72°C, and a subsequent final extension for 10 min at 72°C. PCR components were added as recommended by the manufacturer of Takara LA Taq (Takara Biomedical, Japan). Amplified products were purified and sequenced directly from both strands using the ABI 3730xl DNA Analyzer by Sanboyuanzhi Biotechnology Co., Ltd (Beijing, China).

Two sets of microsatellite primers have been reported for the diamondback moth [7], [76]. Those developed by Endersby et al. [7] were found to produce many null alleles in populations outside Australia [31]. Thus, we used nine microsatellite loci from Esselink et al. [76] (Table S6). All loci were fluorescently labeled and amplified following the method of Schuelke [77] under the annealing temperatures recommended by Esselink et al. [76] for the first 30 cycles. The sizes of the amplified PCR products were determined using the ABI 3730xl DNA Analyzer (Applied Biosystems) with GeneScan 500 LIZ size standard (Applied Biosystems), performed by Tsingke Biotechnology Co., Ltd (Beijing, China). Allele designation was obtained using the software GENEMAPPER, version 4.0 (Applied Biosystems).

### Statistical Analysis

#### Genetic diversity

Sequencing results determined from both strands were assembled using the SEQMAN program within the LASERGENE suite version 7.1.2 (DNASTAR, Inc., USA). Sequences of the five genes were aligned independently using CLUSTALW [78] implemented in MEGA version 5 [79] with default parameters. Alignment of nucleotide sequences of the protein-coding genes was inferred from the amino acid alignment. The number of polymorphic sites (*S*), haplotype diversity (*h*), nucleotide diversity (*π*) and the average number of pairwise differences (*P*) were calculated using the ARLEQUIN suite, version 3.5 [80].

The microsatellite data determined from GENEMAPPER were first checked for stuttering, large allele dropout and null alleles using MICRO-CHECKER version 2.2.3 [81]. Allele frequency, the number of alleles, observed and expected heterozygosity, *F_ST_* and genetic distances were calculated using MICROSATELLITE ANALYSER (MSA) version 4.0.5 [82]. Tests for deviation from Hardy-Weinberg equilibrium at each locus for each population were performed using GENEPOP version 4.0.11 [83].

#### Population structure

For mitochondrial genes, the pairwise *F_ST_* values between each pair of the 27 populations were calculated using the ARLEQUIN suite, version 3.5 [80]. The method of simulated annealing was implemented in the SAMOVA program version 1.0 [84] to examine the genetic structure. The number of groups was set from 2 to 20, and the number of initial conditions was set at 100. The pairwise difference was used to calculate the molecular distance. The values of fixation indices among populations within groups (*F_SC_*), among groups (*F_CT_*) and within populations (*F_ST_*) were compared among different group numbers within each dataset. For microsatellite data, population differentiation was investigated using the Bayesian clustering approach implemented in the program STRUCTURE, version 2.3.3 [85]. The admixture ancestry model and the correlated allele frequency model with a burn-in period of 50000 iterations and 1 million Markov chain Monte Carlo repetitions were used to calculate the probable number of genetic clusters (*K*). We performed 35 independent runs for each *K* (from 1 to 20) to confirm consistency across runs. The most accurate number of groups (*K*) was visually examined when plotting *K* against Δ*K* and using the Evanno method in the online program STRUCTURE HARVESTER [86].

To establish whether any isolation-by-distance effect occurred, matrices of genetic distance data [*F_ST_*/(1-*F_ST_*)] and the logarithms of geographical distance (ln Km) between all the sampling sites were constructed. These matrices were analyzed for their degree of correlation using a Mantel test [87] implemented in the software ZT version, 1.1 [88].

#### Gene flow

To test whether there was asymmetric dispersal between populations, the mutation-scaled population size (θ = *N_e_*µ, where μ is the mutation rate per site per generation) and the mutation-scaled migration rate (*M* = *m*/µ, where m is the migration rate) were calculated using Bayesian search strategies and the software MIGRATE, version 3.2.16 [89]. The 27 populations were either directly used or divided into three geographical groups–i.e., south, middle and north (Table 4)–for analysis. The effective number of migrants entering and leaving each population per generation *xN_e_m* is θ*M* (*x* is a multiplier that depends on the ploidy and inheritance of the data, and here, *x* is one for mitochondrial DNA). The parameters used in the calculations are as follows: long-chains = 1, long-inc = 20, long-sample = 1000000, burn-in = 1000000, heating = YES: 1: (1.0, 1.5, 3.0, 6.0), heated-swap = YES and replicate = YES: 5. Four runs of MIGRATE analysis were conducted to verify the consistency in our results. For each run, we changed the random number seed and starting values for θ and *M*. In the first run, θ and *M* were estimated from *F_ST_* values and in the subsequent runs. In the subsequent runs, Bayesian estimates of θ and *M* from the previous run were used. The estimated θ, *M* and θ*M* from the final run are reported here.

#### Haplotype phylogeny and network analysis

The phylogenetic relationship among the 350 haplotypes from the combined genes of *cox1*, *atp8*, *atp6* and *nad5* sequenced in our study were constructed. To compare the phylogenetic relationship of the haplotypes from China and other regions, the partial sequence of the *cox1* gene of the diamondback moth was downloaded from GenBank. There are 163 sequences with homologous regions to the *cox1* segment sequenced in our study. A total of 106 sequences with information regarding sampling location were left for further analysis. Phylogenetic inferences were conducted using the Bayesian method (BI) in the software MRBAYES, version 3.2 [90] and the Neigbor-Joining (NJ) method in the software MEGA version 5 [79]. The software jModeltest version 0.1.1 was used to aid the selection of the best-fit nucleotide substitution model [91]. The GTR+I+G model for four-state nucleotide sequences was used in the BI analysis. The data matrix was partitioned by three codon positions. Bayesian analyses were conducted with eight independent Markov chains that were run for 10,000,000 Metropolis-coupled MCMC generations, with tree sampling every 1000 generations and a burn-in of 2500 trees. Trees were visualized and produced using FIGTREE software version 1.3.1 (http://tree.bio.ed.ac.uk/software/figtree/). For the NJ method, the LogDet (Tamura-Kumar) model was used for analysis. The species *Leucoptera malifoliella* (Lepidoptera: Yponomeutoidea: Lyonetiidae) was selected as outgroup, because this is the evolutionary nearest species to the diamondback moth with sequenced mitochondrial genome sequence [92].

The haplotype network of each *cox1*, *atp6*, *nad5* gene and the combined genes were inferred using the median-joining algorithm [93]. Before the calculation, the star contraction method with a maximum star radius value of 10 was used to simplify the data matrix of the *cox1* gene and the combined genes [94]. The “Frequency>1” criterion, which simplifies networks by ignoring unique haplotypes in the data set, was used, and the epsilon value was set to 0 for the calculation. After the calculation, the MP calculation was used to identify and clean up unnecessary median vectors and links [95]. All the above calculations were conducted using the software Network, version 4.6.1.0 (Fluxus Technology Ltd, England). The network’s results were drawn and prepared using the software Network Publisher, version 1.3.0.0 (Fluxus Technology Ltd, England). The shortest trees with median vectors were shown.

#### Demographic analysis

The demographic history of each population was examined. Background selection using Tajima’s D [96], and Fu’s F statistics [97] were tested. The distribution of pairwise differences between individual sequences was analyzed using mismatch distribution analysis [98], [99]. Both analyses were conducted using the program ARLEQUIN suite, version 3.5 [80].

Bayesian Skyline Plot implemented in the software BEAST, version 1.7.2 [100], was used to investigate the population history of the diamondback moth by estimating changes in the effective population size over time [101]. Each of the 27 populations was analyzed independently. Because all individuals were collected around the year 2010 or 2011, we set the date value as 0 to be default. The SRD06 model was used for analysis. This model links 1st and 2nd codon positions but allows the 3rd positions to have a different relative rate of substitution, transition-transversion ratio and gamma-distributed rate heterogeneity. The strict clock model was used with the estimated rate. The prior uniform distribution was used for the rate clock with a lower value of 0 and upper value of 1. Two independent runs with 100 million generations were performed. Samples from the Markov Chain were taken every 10000 steps. The output of BEAST was compared and analyzed using Tracer, version 1.5 (http://tree.bio.ed.ac.uk/software/tracer/). When the two runs were converging on the same distribution, one of them was used for Bayesian Skyline Reconstruction analysis. Otherwise, additional runs were performed with increased generations until two of them were converging.

## Supporting Information

Figure S1 **The values of fixation indices among populations within groups (** ***F_SC_*** **), among groups (** ***F_CT_*** **) and within populations (** ***F_ST_*** **) when the 27 populations are divided into 2 to 20 geographical groups, calculated using SAMOVA based on the combined mitochondrial genes of** ***cox1*** **,** ***atp8*** **,** ***atp6*** **and** ***nad5*** **.**
(PDF)

Figure S2 **The haplotype network of the gene** ***cox1*** **.** The area of a circle is proportional to the number of observed individuals. Colors within the nodes indicate the following: red, southern China; green, middle regions of China; blue, northern China.
(PDF)

Figure S3 **The haplotype network of the gene** ***atp6*** **.** The area of a circle is proportional to the number of observed individuals. Colors within the nodes indicate the following: red, southern China; green, middle regions of China; blue, northern China.
(PDF)

Figure S4 **The haplotype network of the gene** ***nad5*** **.** The area of a circle is proportional to the number of observed individuals. Colors within the nodes indicate the following: red, southern China; green, middle regions of China; blue, northern China.
(PDF)

Figure S5 **Mismatch distribution of the combined genes of** ***cox1*** **,** ***atp8*** **,** ***atp6*** **and** ***nad5*** **in the 27 populations of the** ***Plutella xylostella*** **from China using the ARLEQUIN suite, version 3.5**
(PDF)

Figure S6 **Bayesian Skyline Plot of the 27 populations of the** ***Plutella xylostella*** **from China based on the combined genes of** ***cox1*** **,** ***atp8*** **,** ***atp6*** **and** ***nad5*** **.**
(PDF)

Table S1 **Genetic diversity of the five mitochondrial genes and demographic analysis of the 27** ***Plutella xylostella*** **populations as one group.**
(DOCX)

Table S2 **Summary statistics of the nine microsatellite loci examined in the 27 populations of the** ***Plutella xylostella*** **.**
(DOCX)

Table S3 **Estimates (** ***M*** **and θ) of the migration among 27** ***Plutella xylostella*** **populations from China based on combined genes.**
(DOC)

Table S4 **The number of the dominant haplotypes in the 27 populations.** Hap4, Hap31 and Hap8 are the three most dominant haplotypes in the combined genes, and Hap4, Hap2 and Hap2 are the most dominant haplotypes in the genes *cox1*, *atp6* and *nad5*, respectively.
(DOCX)

Table S5 **Mitochondrial genes and their amplification conditions used in this study.**
(DOCX)

Table S6 **Microsatellite loci used in this study.**
(DOCX)
