# Supplementary figures and images for: Genetic Structure and Demographic History Reveal Migration of the Diamondback Moth Plutella xylostella (Lepidoptera: Plutellidae) from the Southern to Northern Regions of China
Source: PLoS One. 2013 Apr 2;8(4):e59654. doi: 10.1371/journal.pone.0059654 (PMC3614937; doi:10.1371/journal.pone.0059654)

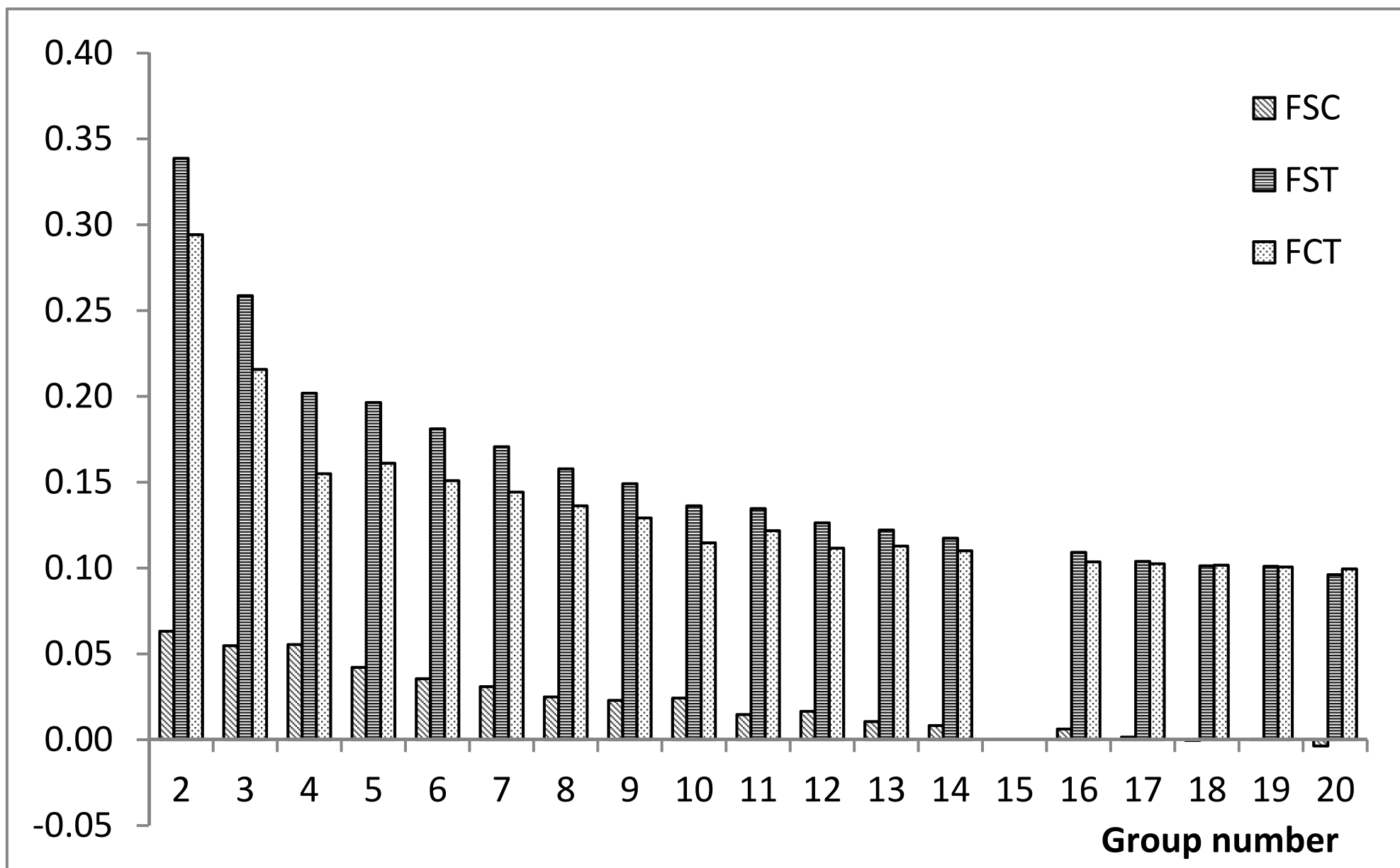

Supplement: Figure S1 — The values of fixation indices among populations within groups ( FSC ), among groups ( FCT ) and within populations ( FST ) when the 27 populations are divided into 2 to 20 geographical groups, calculated using SAMOVA based on the combined mitochondrial genes of cox1 , atp8 , atp6 and nad5 . (PDF) [file pone.0059654.s001.pdf]

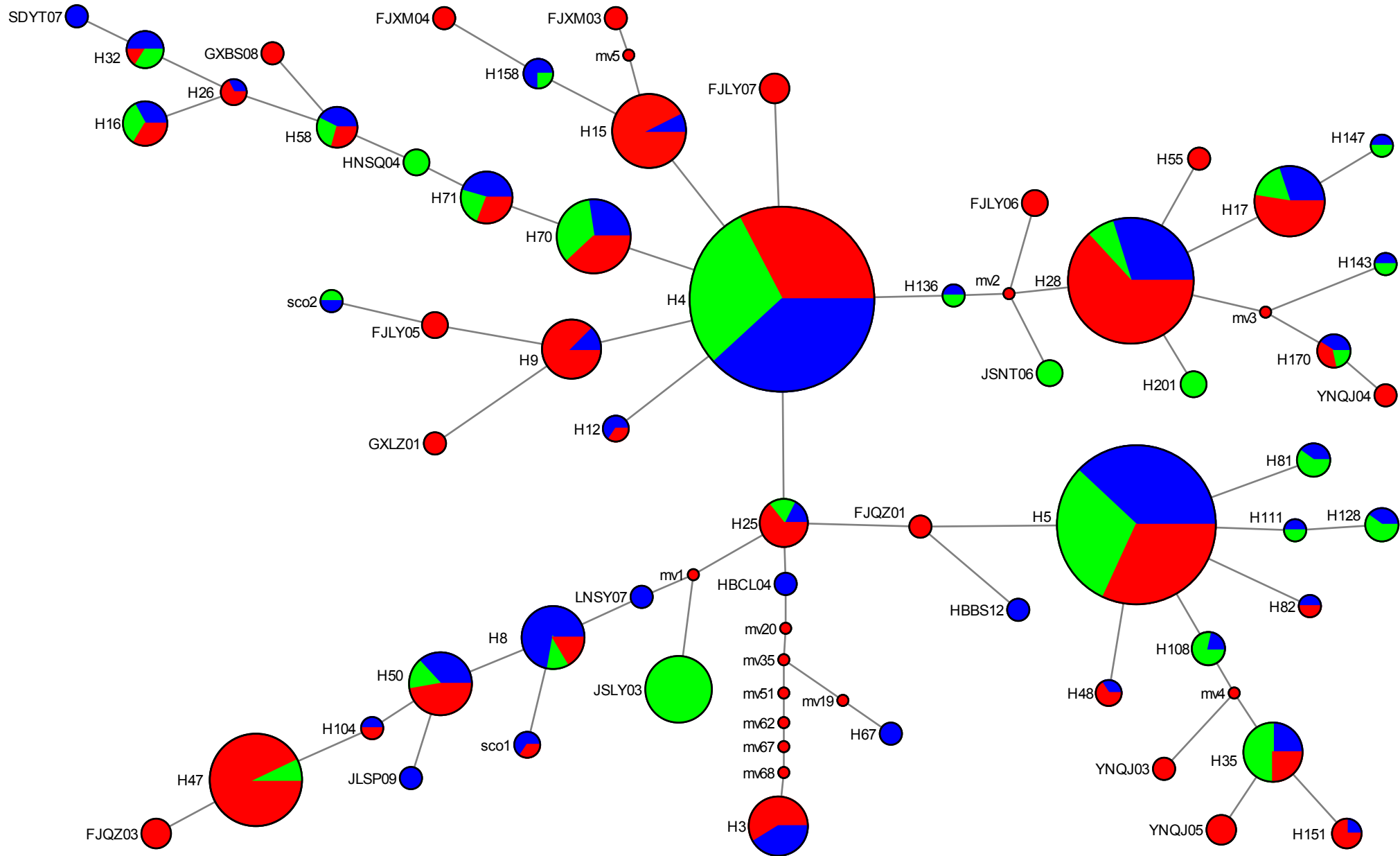

Supplement: Figure S2 — The haplotype network of the gene cox1 . The area of a circle is proportional to the number of observed individuals. Colors within the nodes indicate the following: red, southern China; green, middle regions of China; blue, northern China. (PDF) [file pone.0059654.s002.pdf]

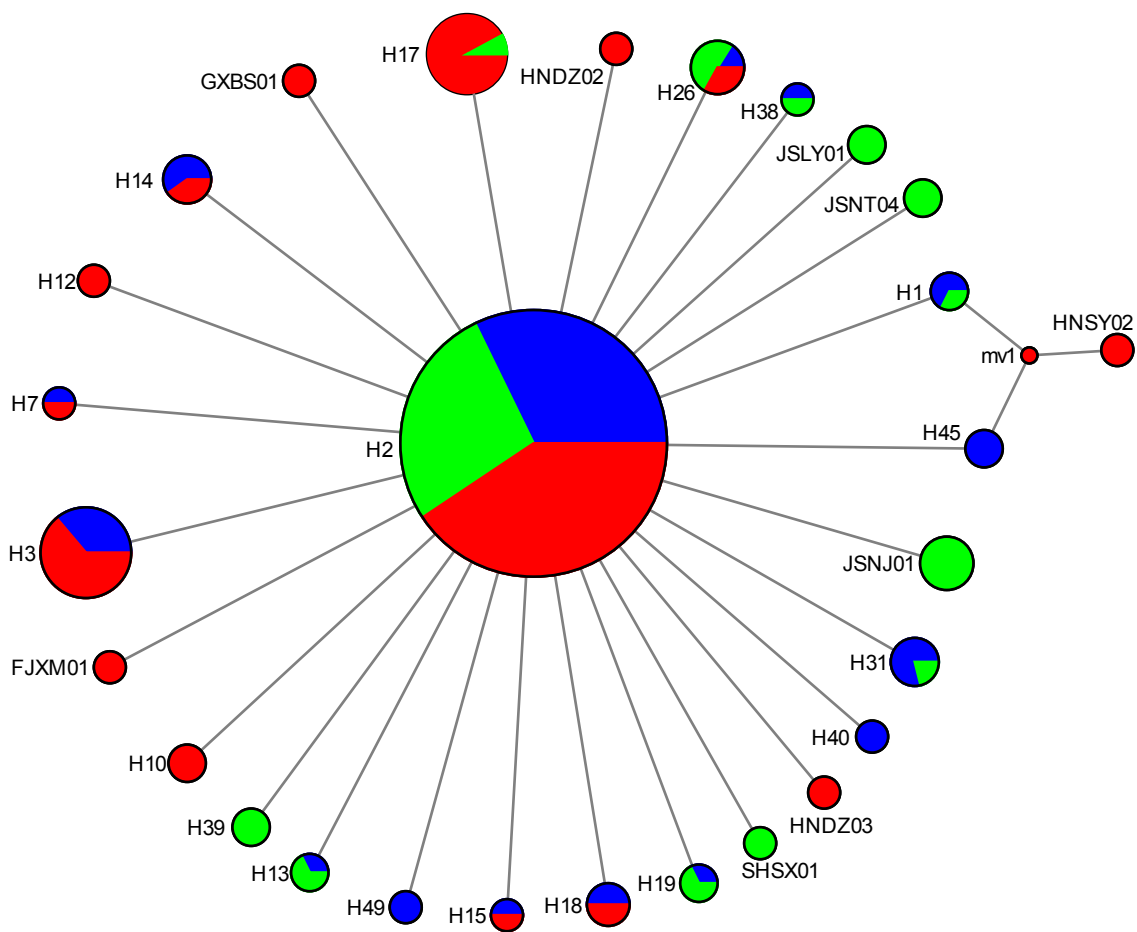

Supplement: Figure S3 — The haplotype network of the gene atp6 . The area of a circle is proportional to the number of observed individuals. Colors within the nodes indicate the following: red, southern China; green, middle regions of China; blue, northern China. (PDF) [file pone.0059654.s003.pdf]

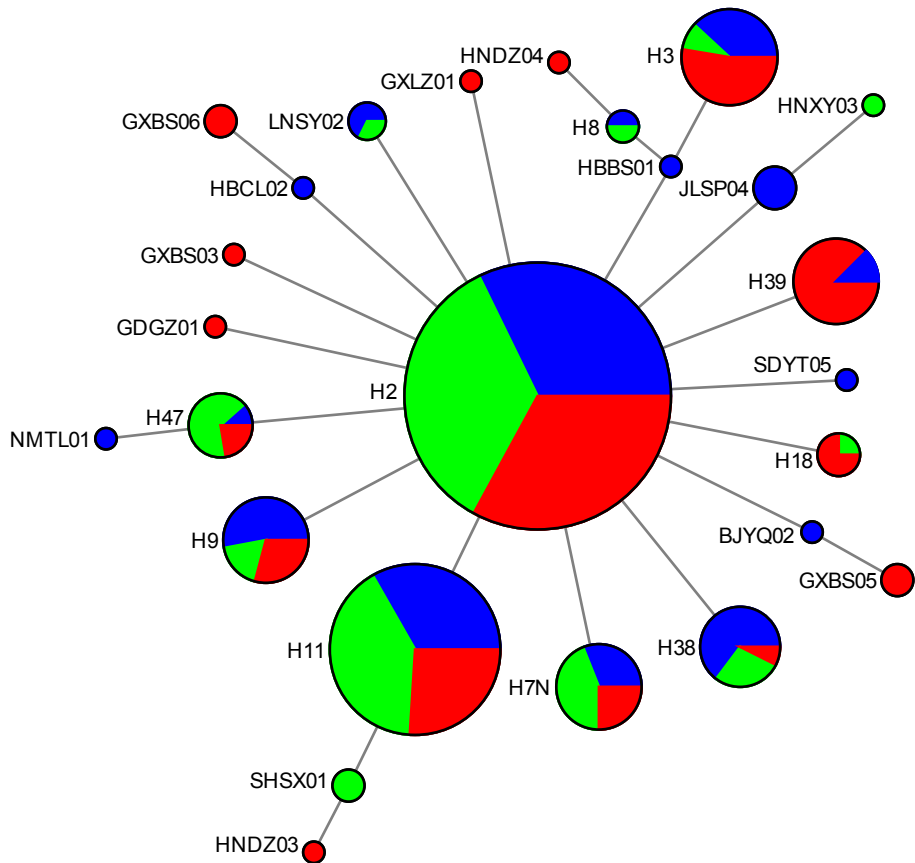

Supplement: Figure S4 — The haplotype network of the gene nad5 . The area of a circle is proportional to the number of observed individuals. Colors within the nodes indicate the following: red, southern China; green, middle regions of China; blue, northern China. (PDF) [file pone.0059654.s004.pdf]

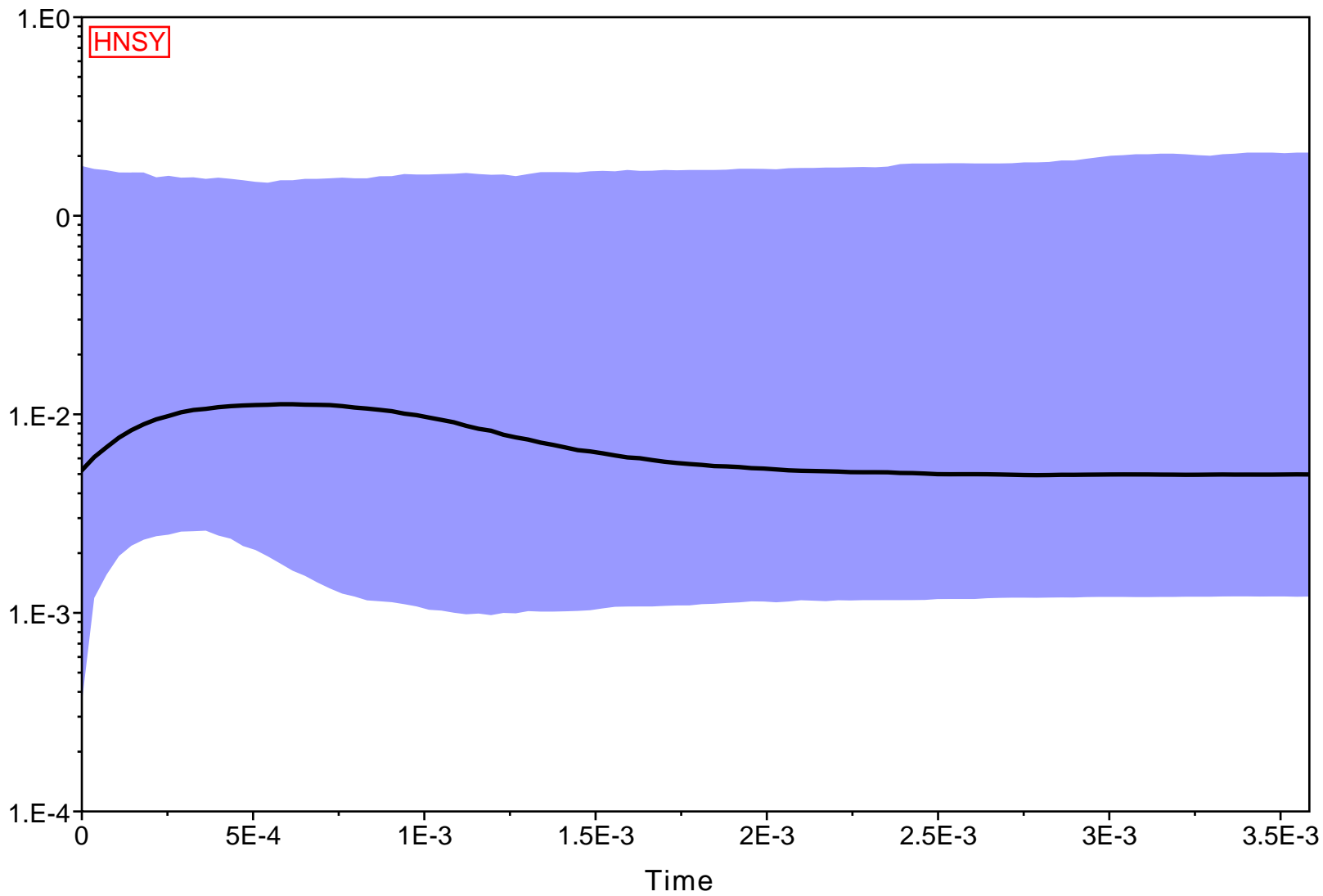

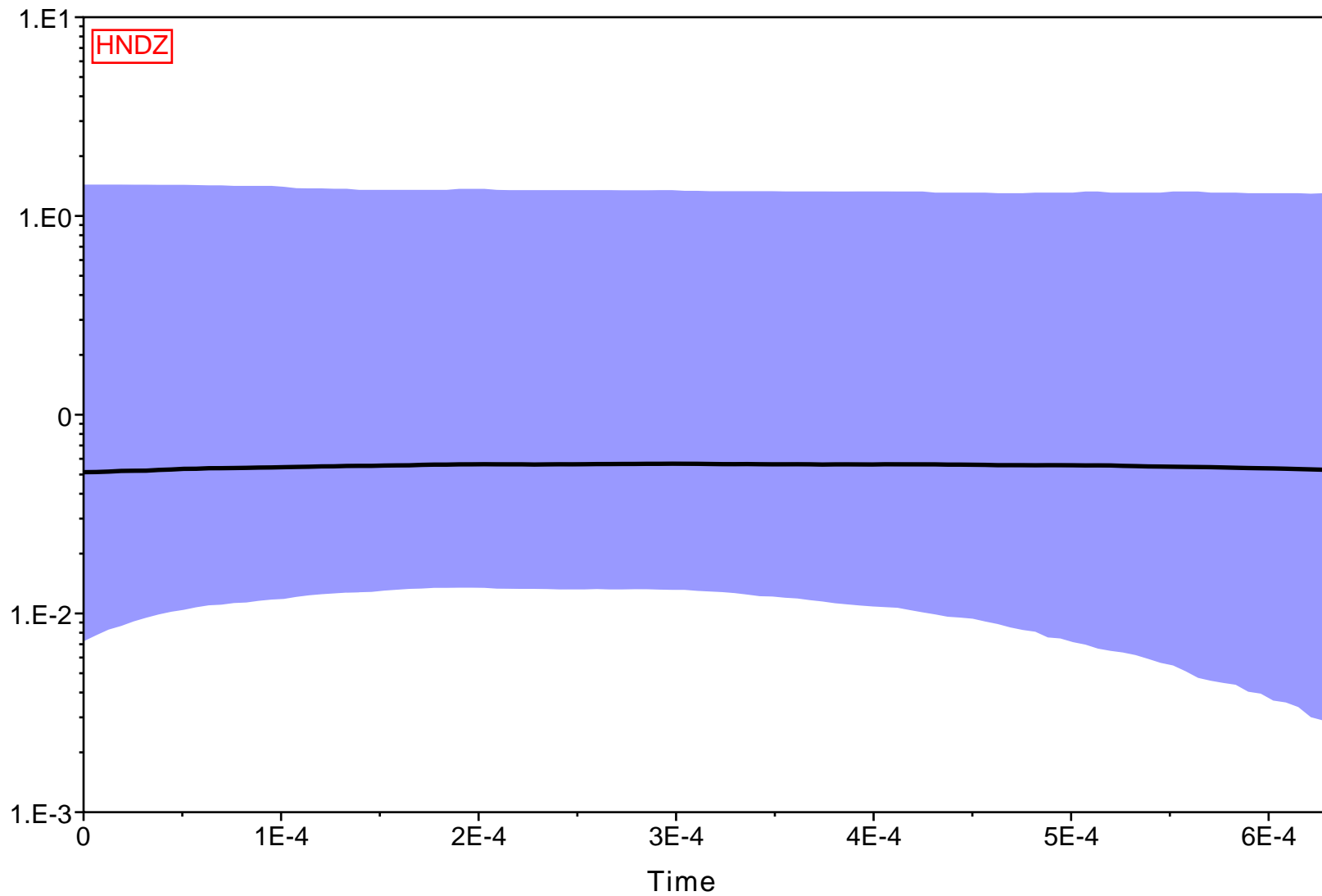

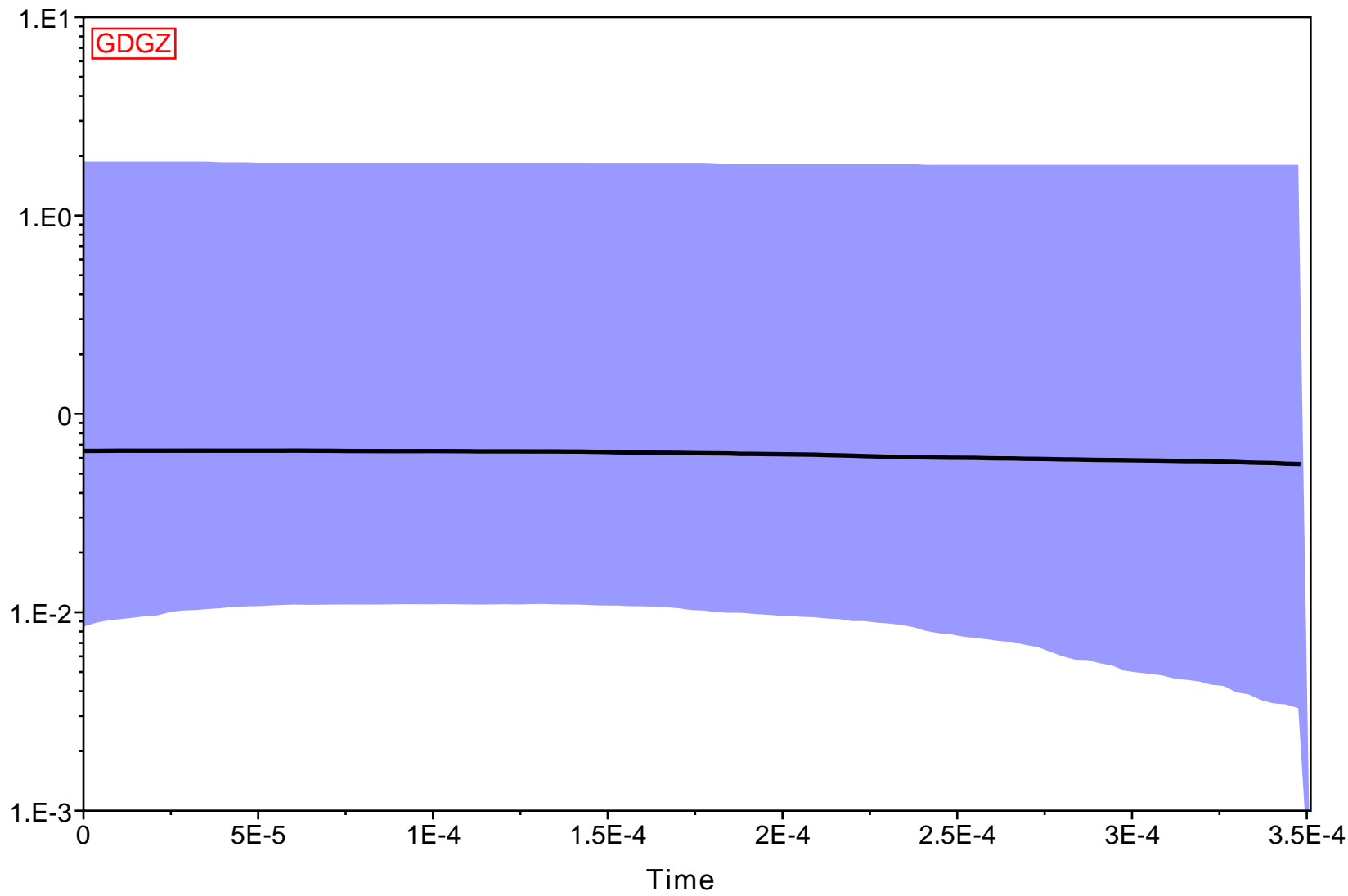

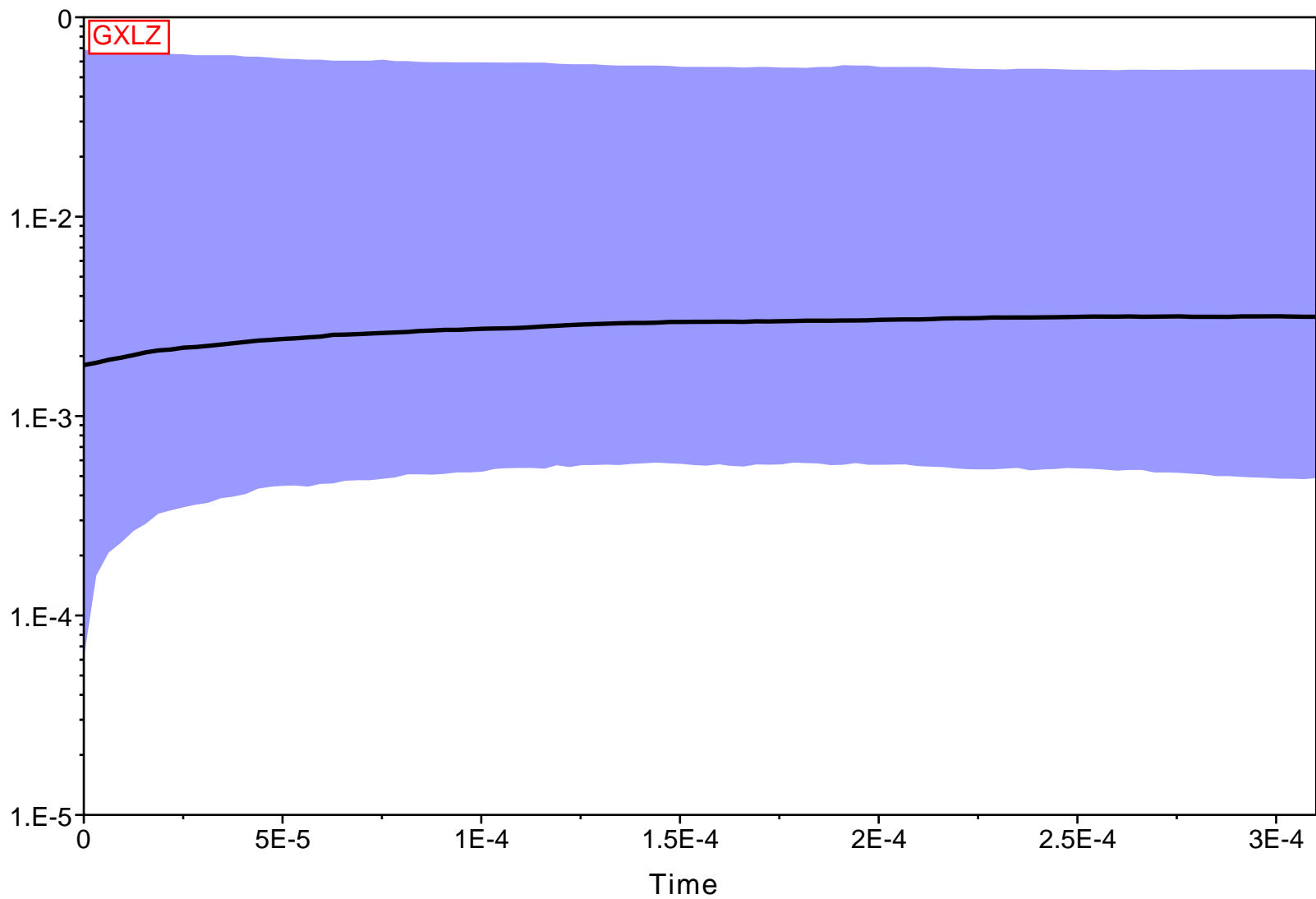

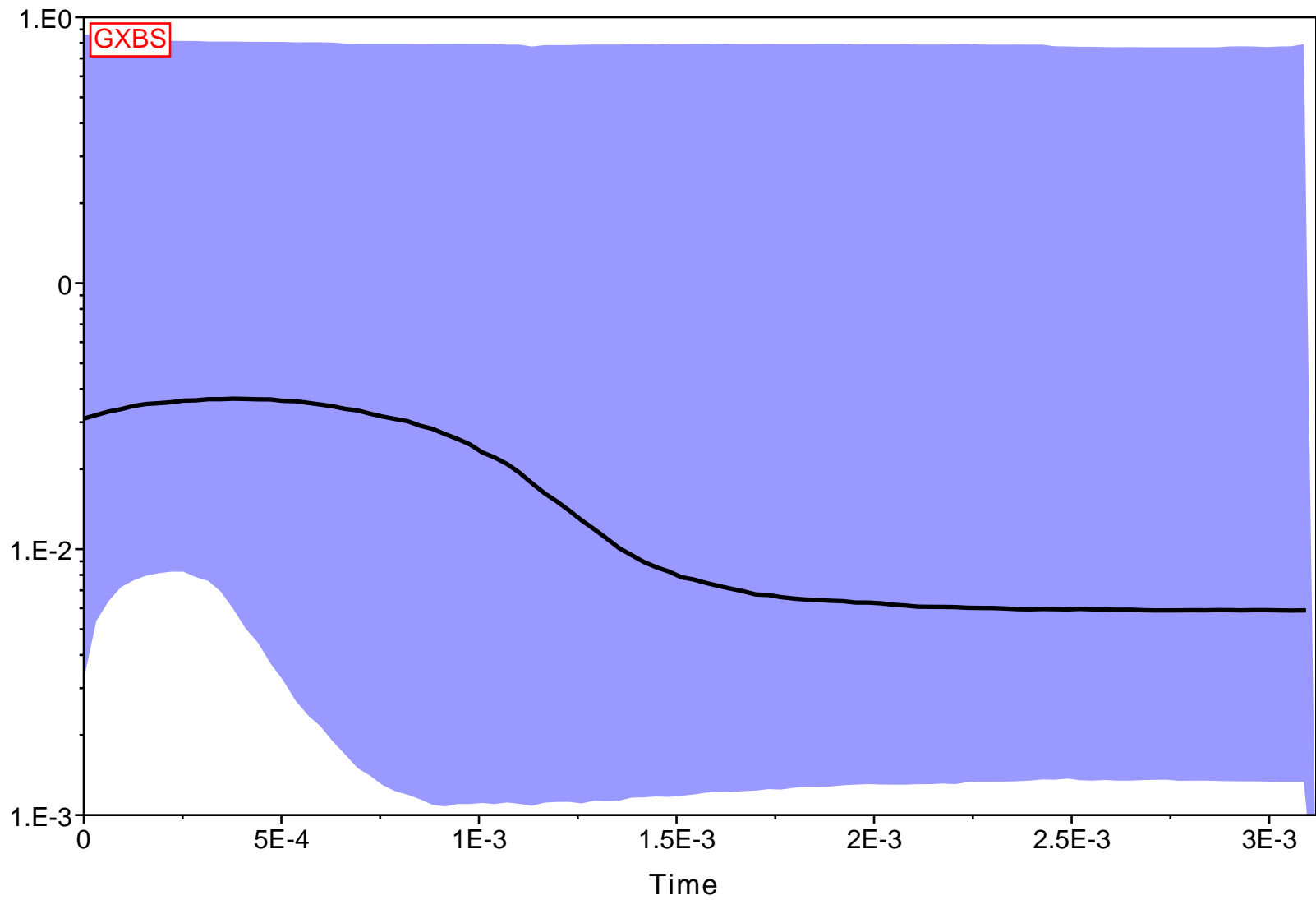

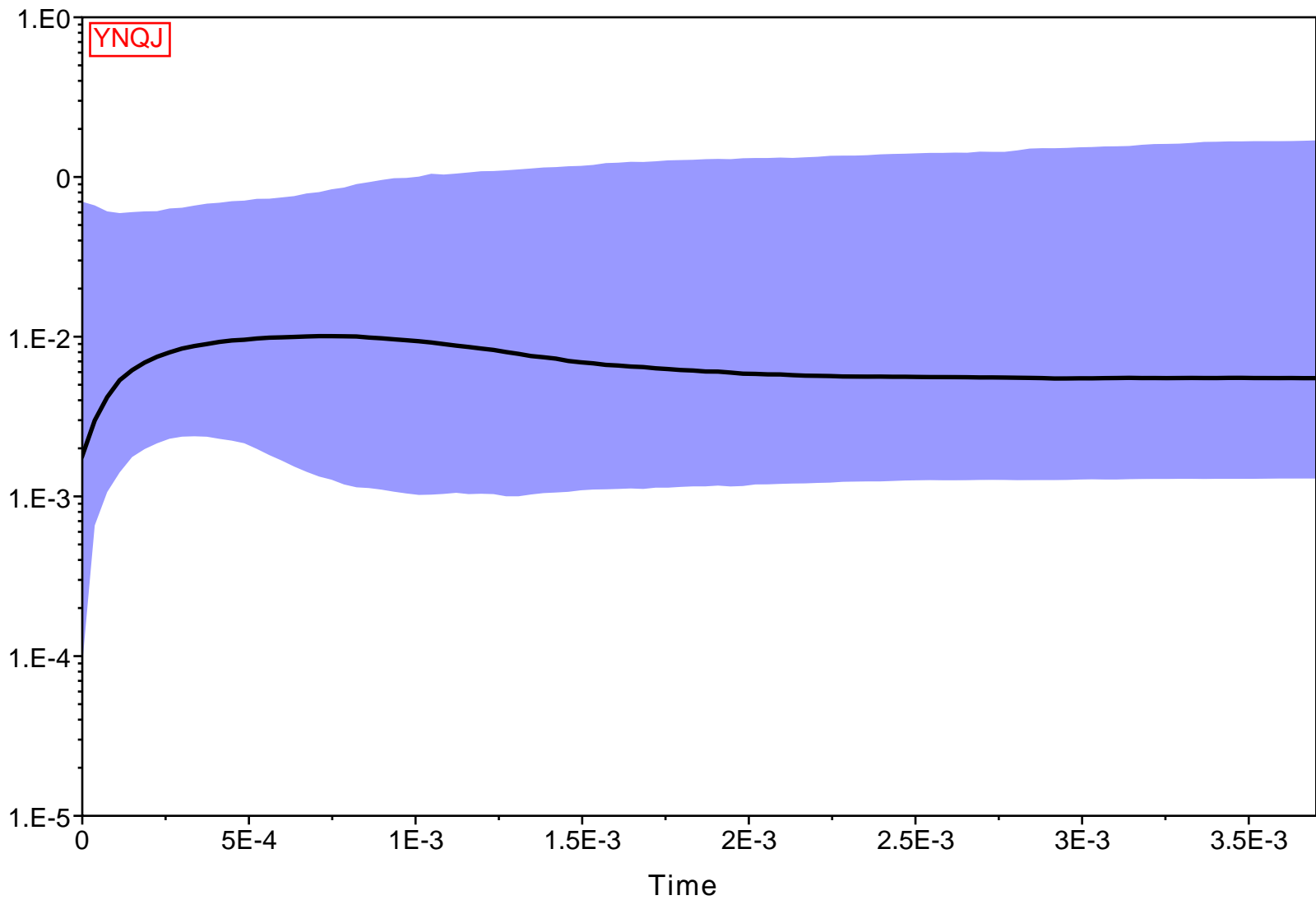

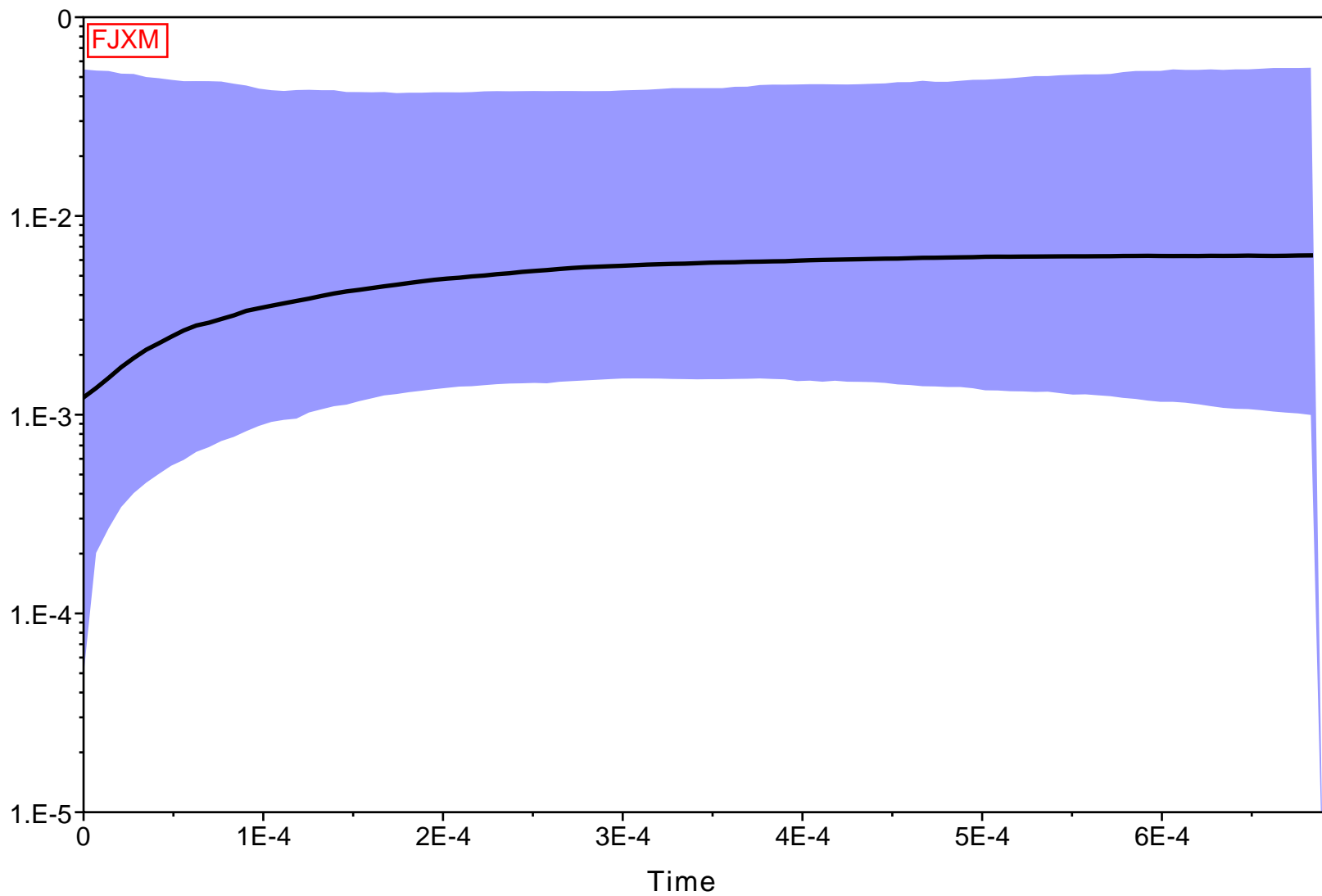

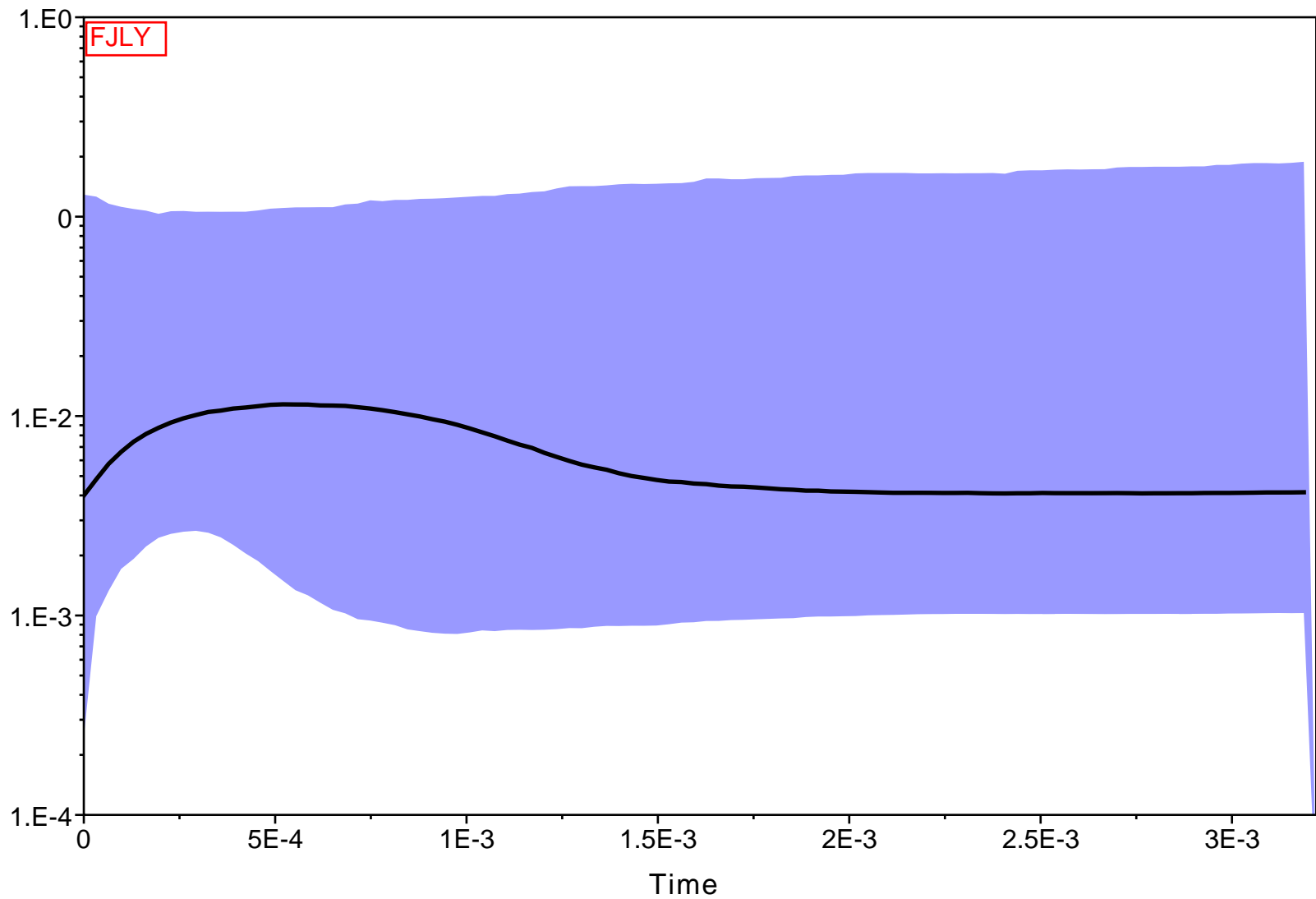

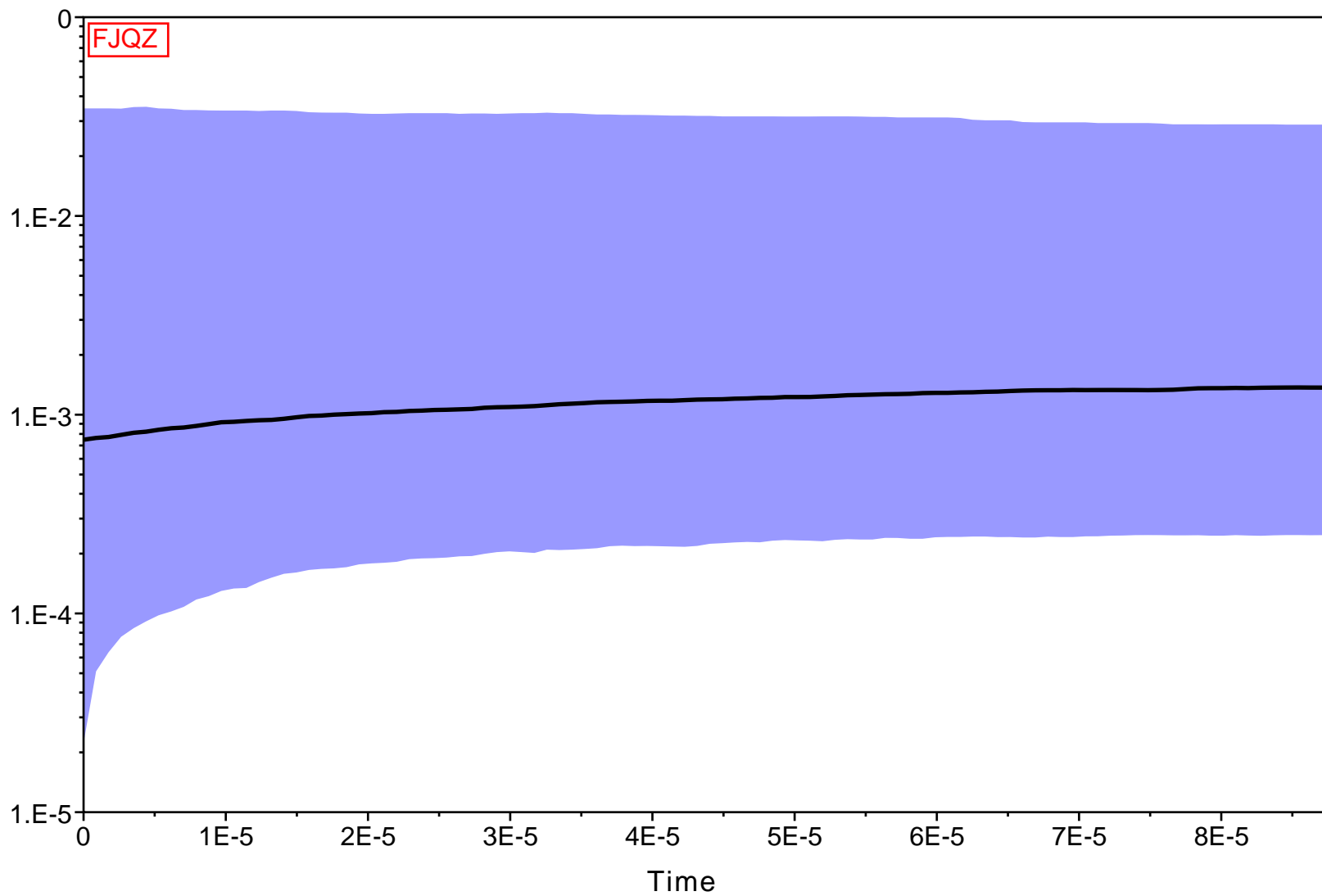

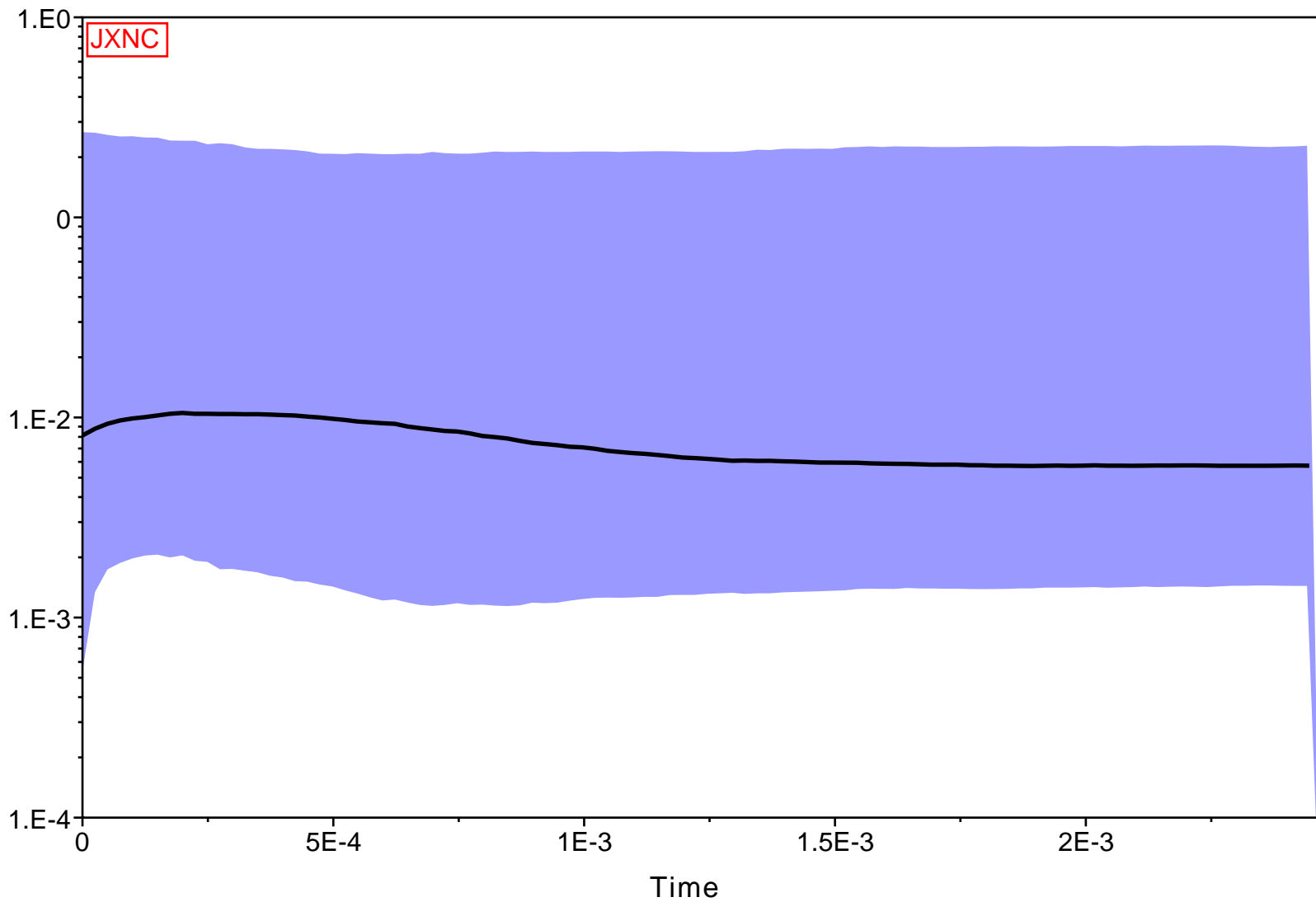

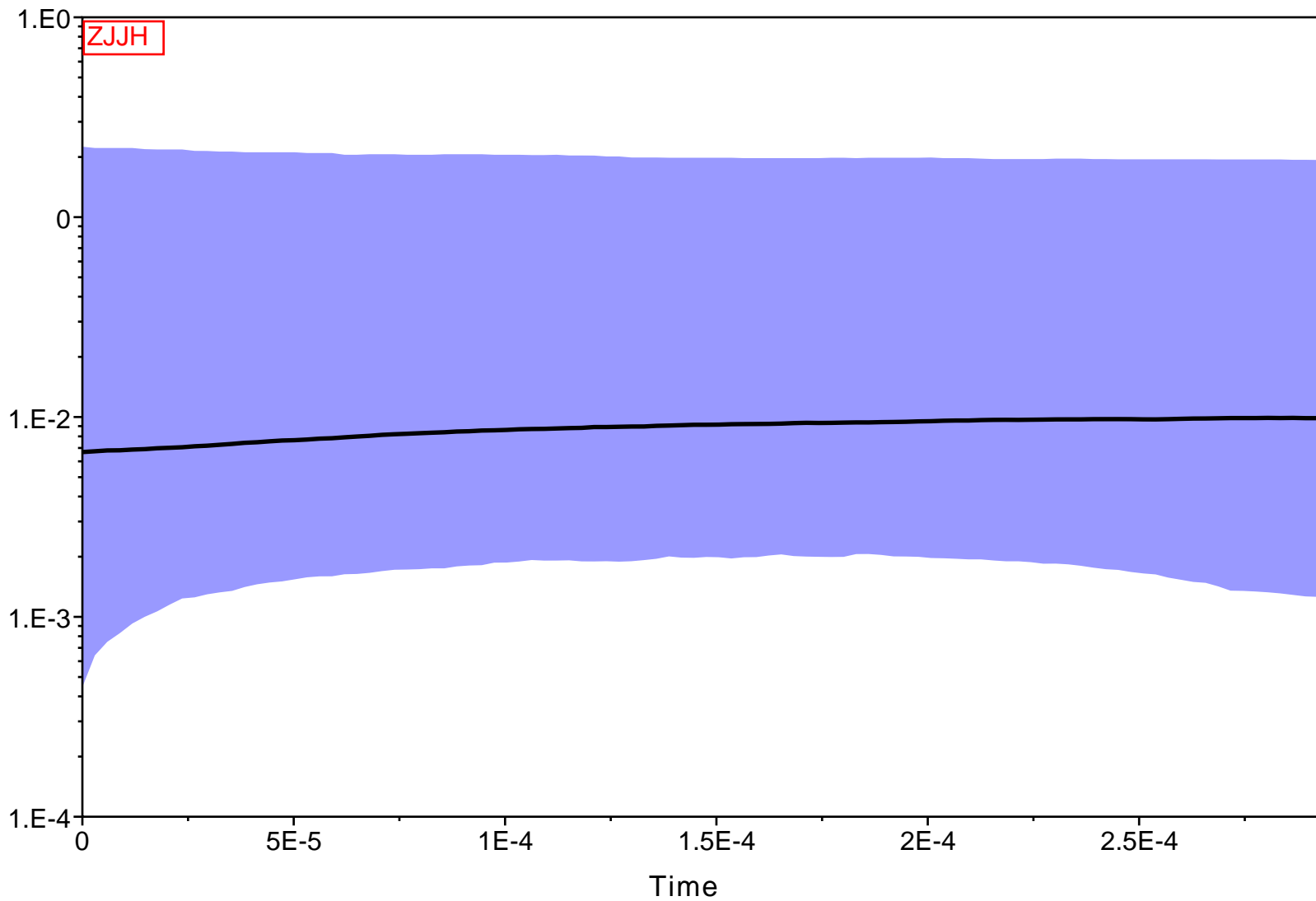

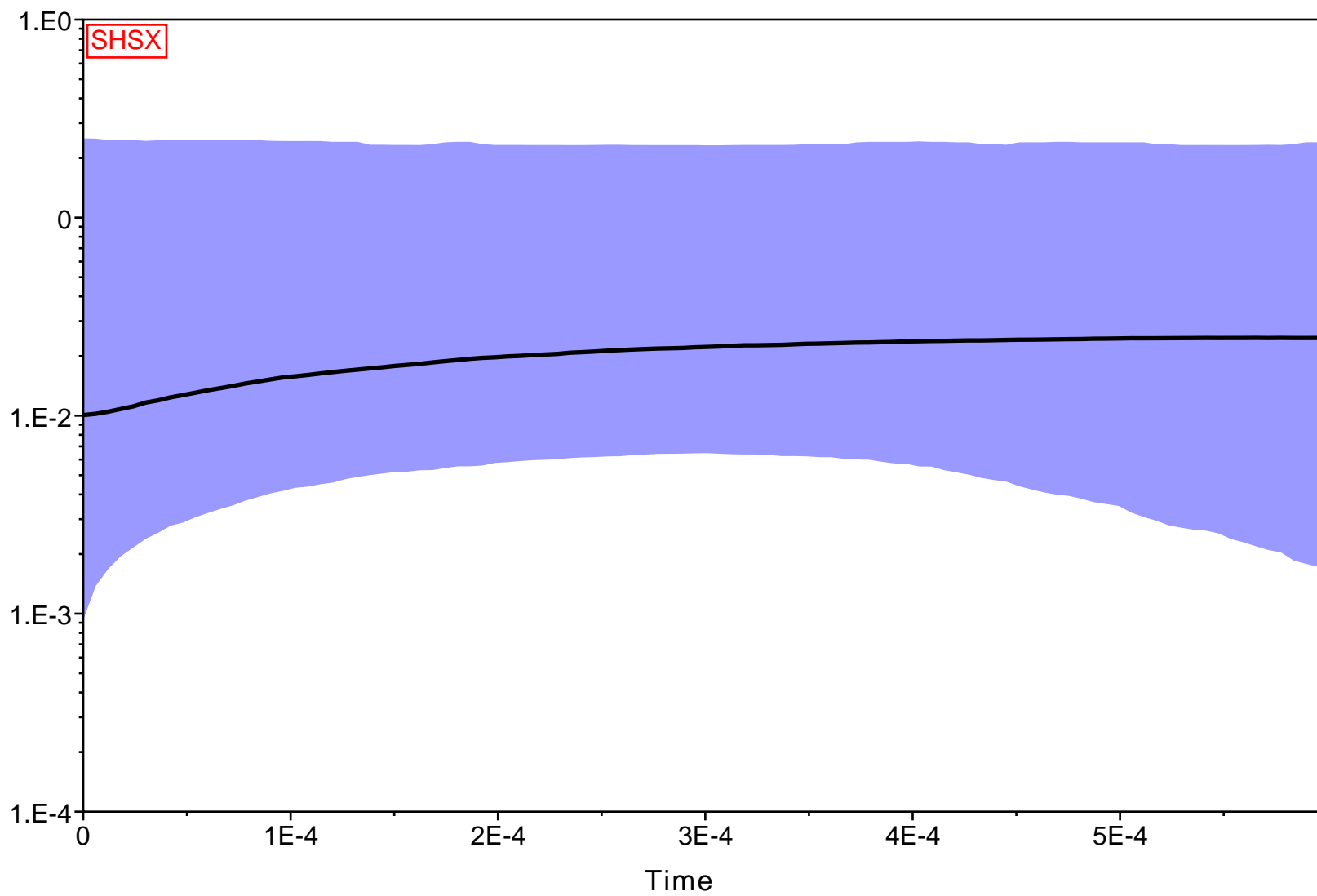

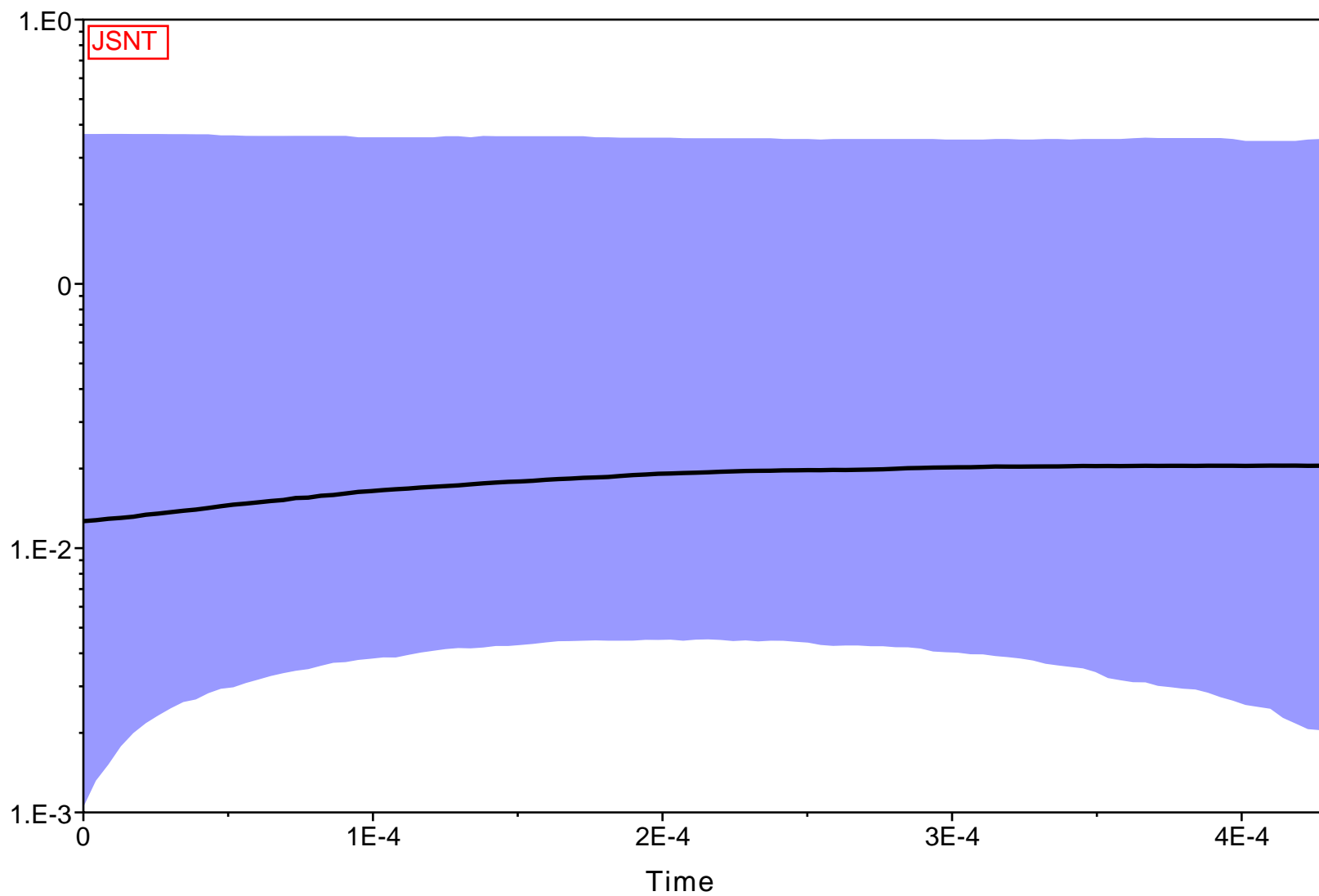

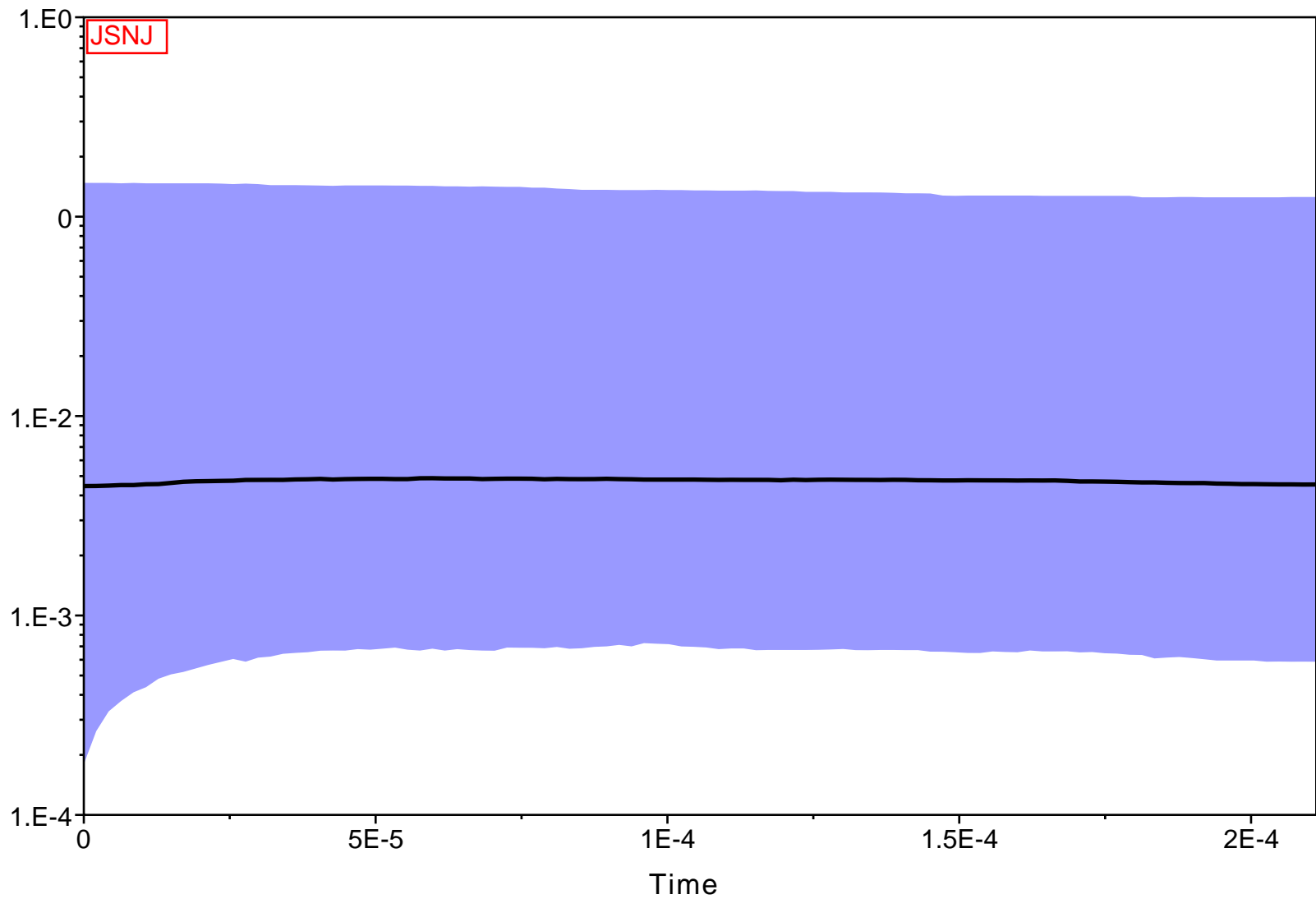

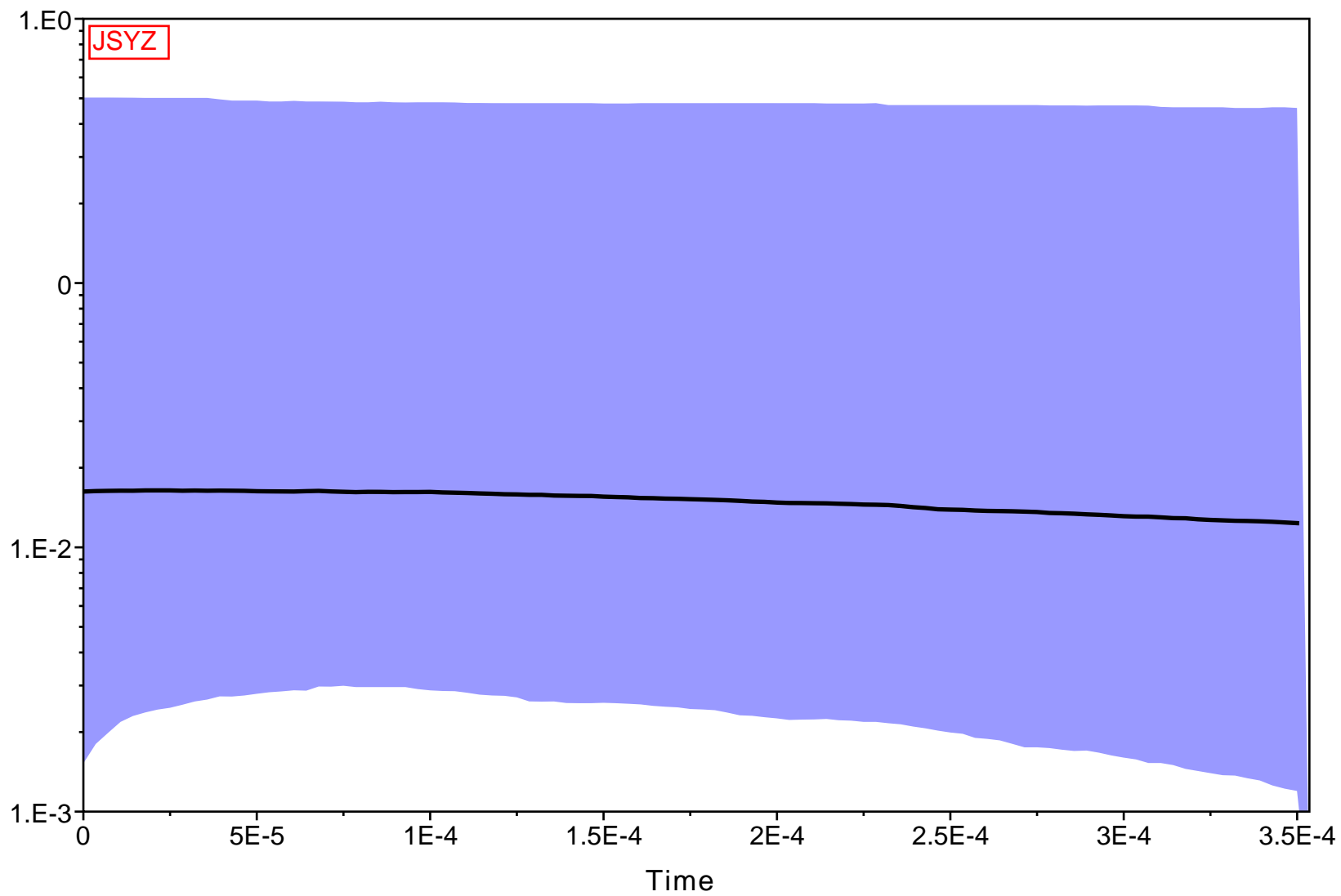

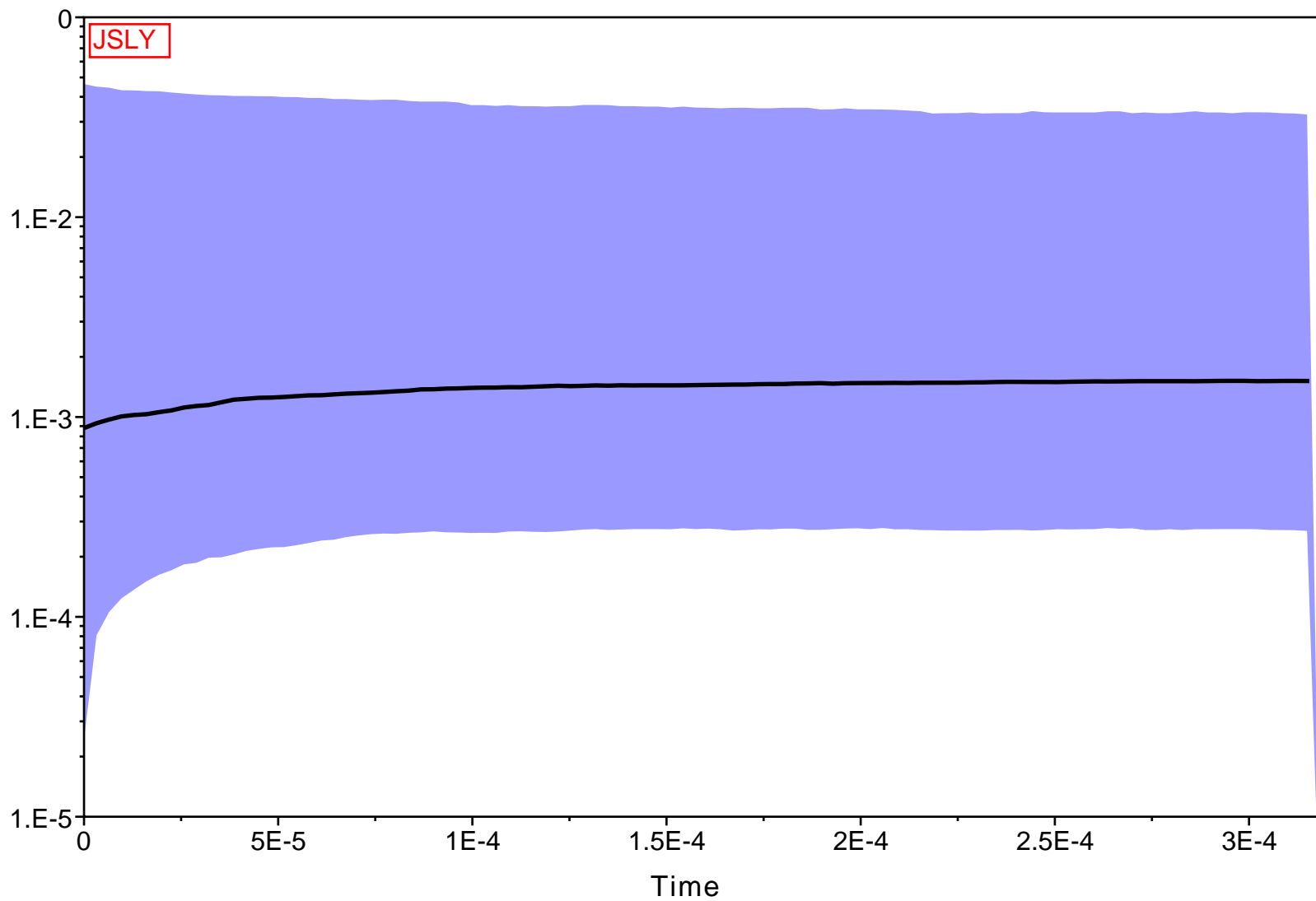

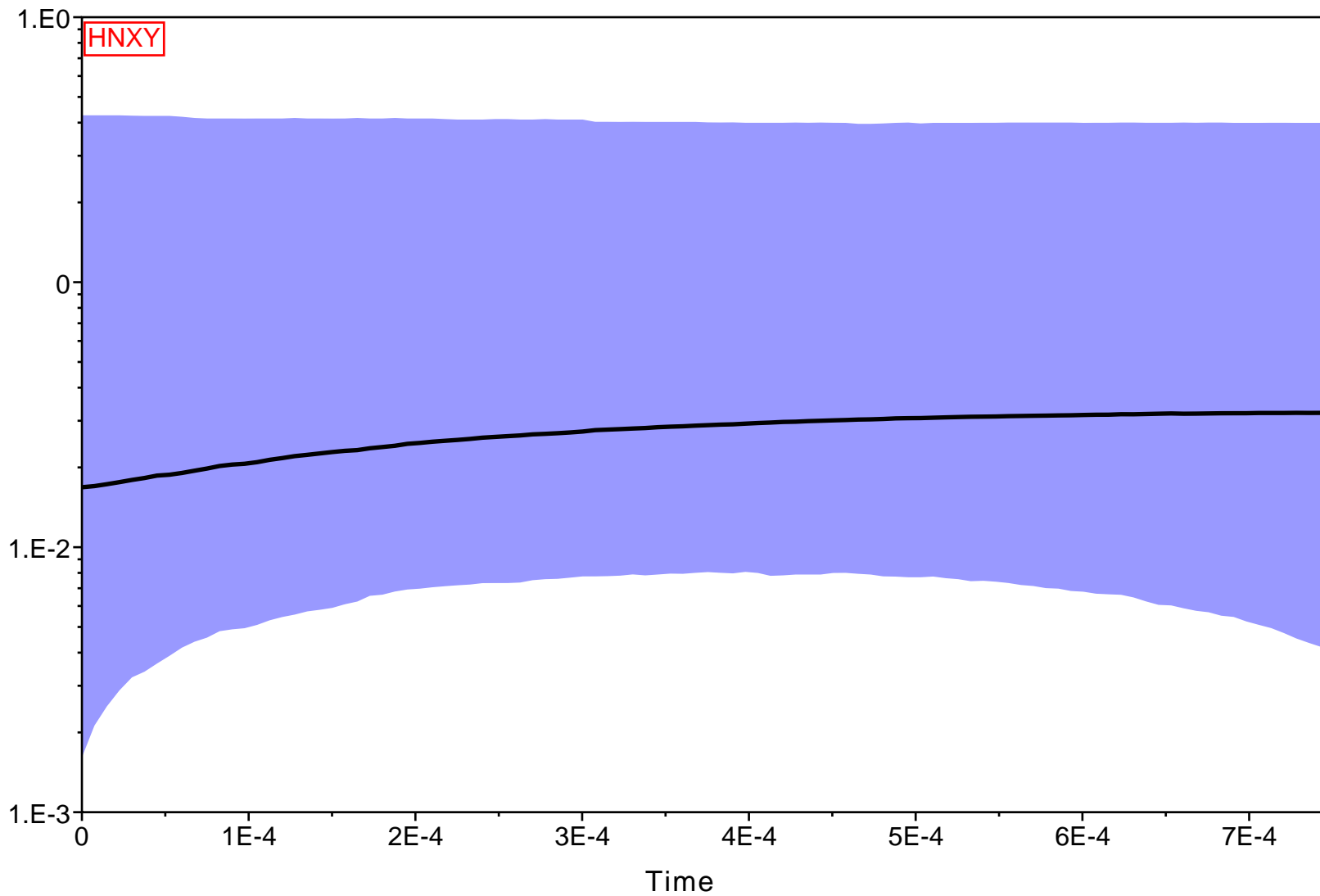

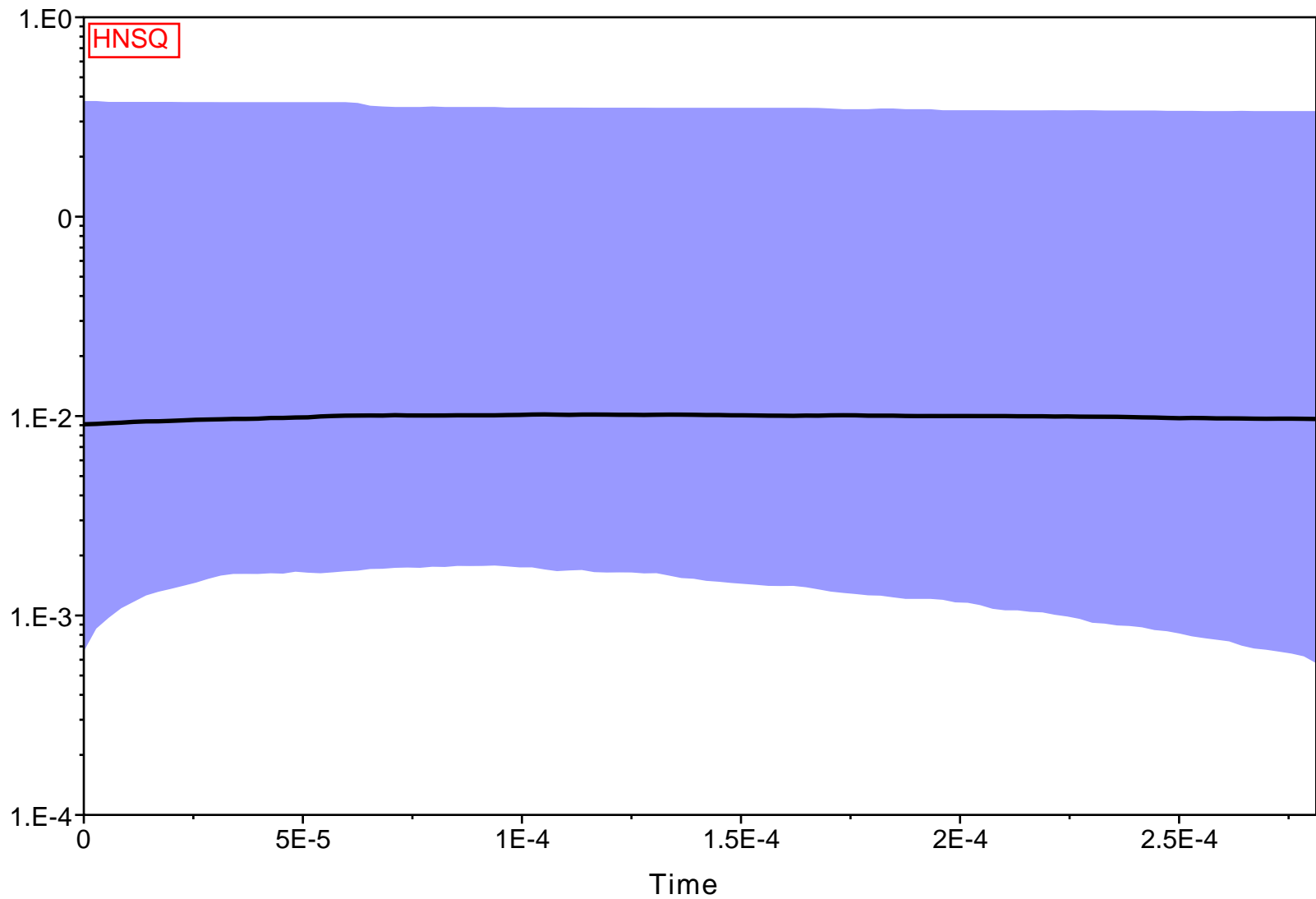

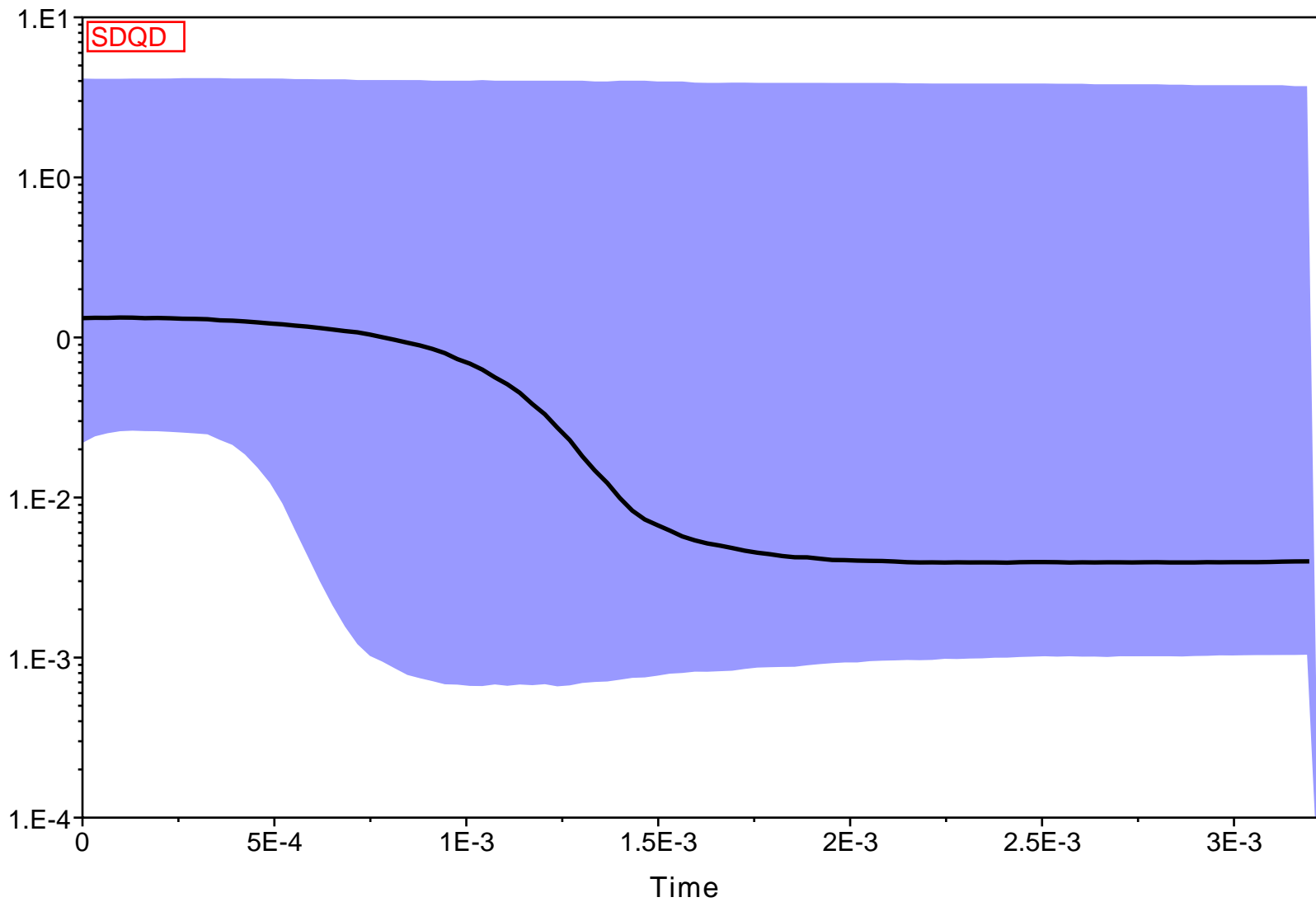

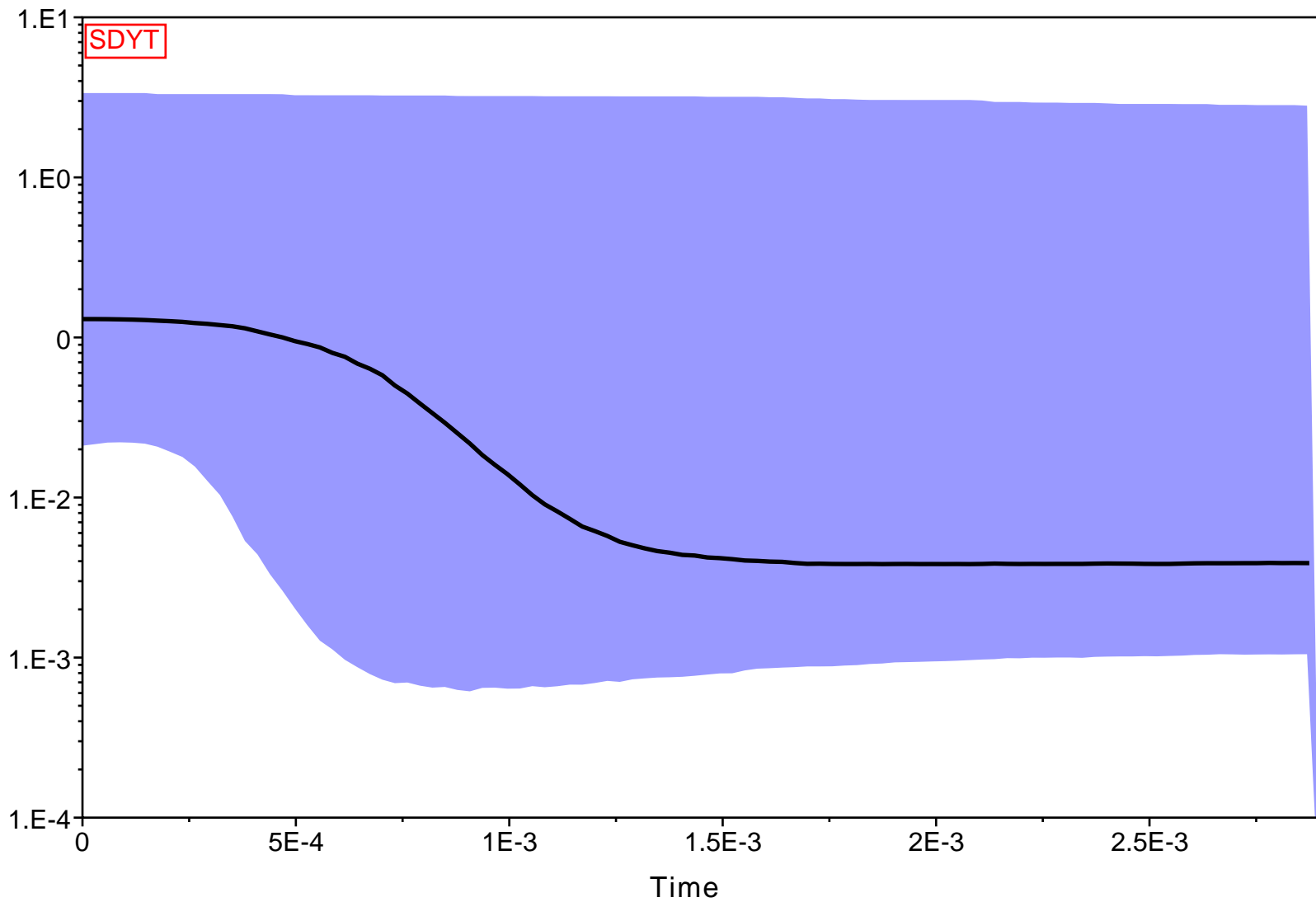

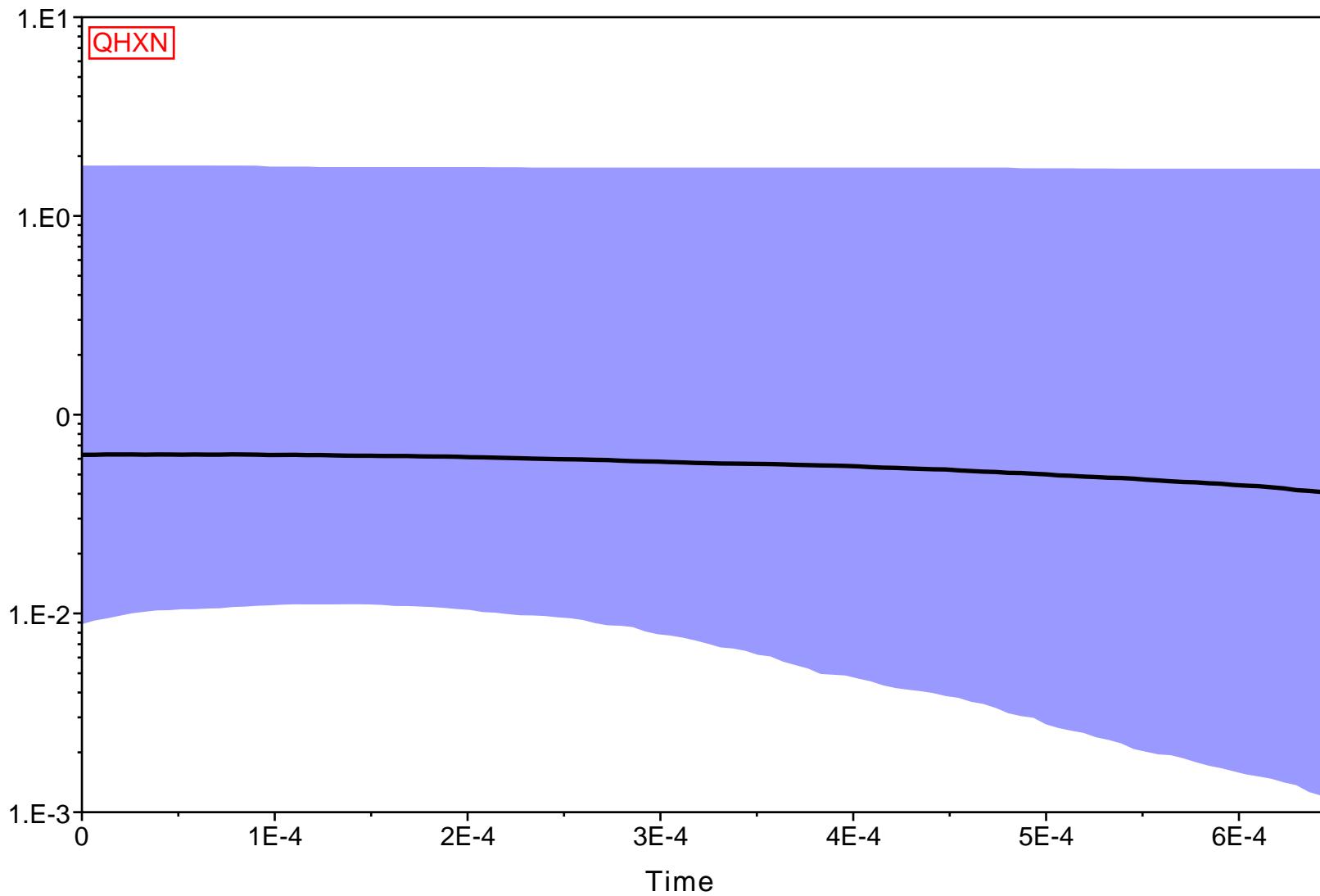

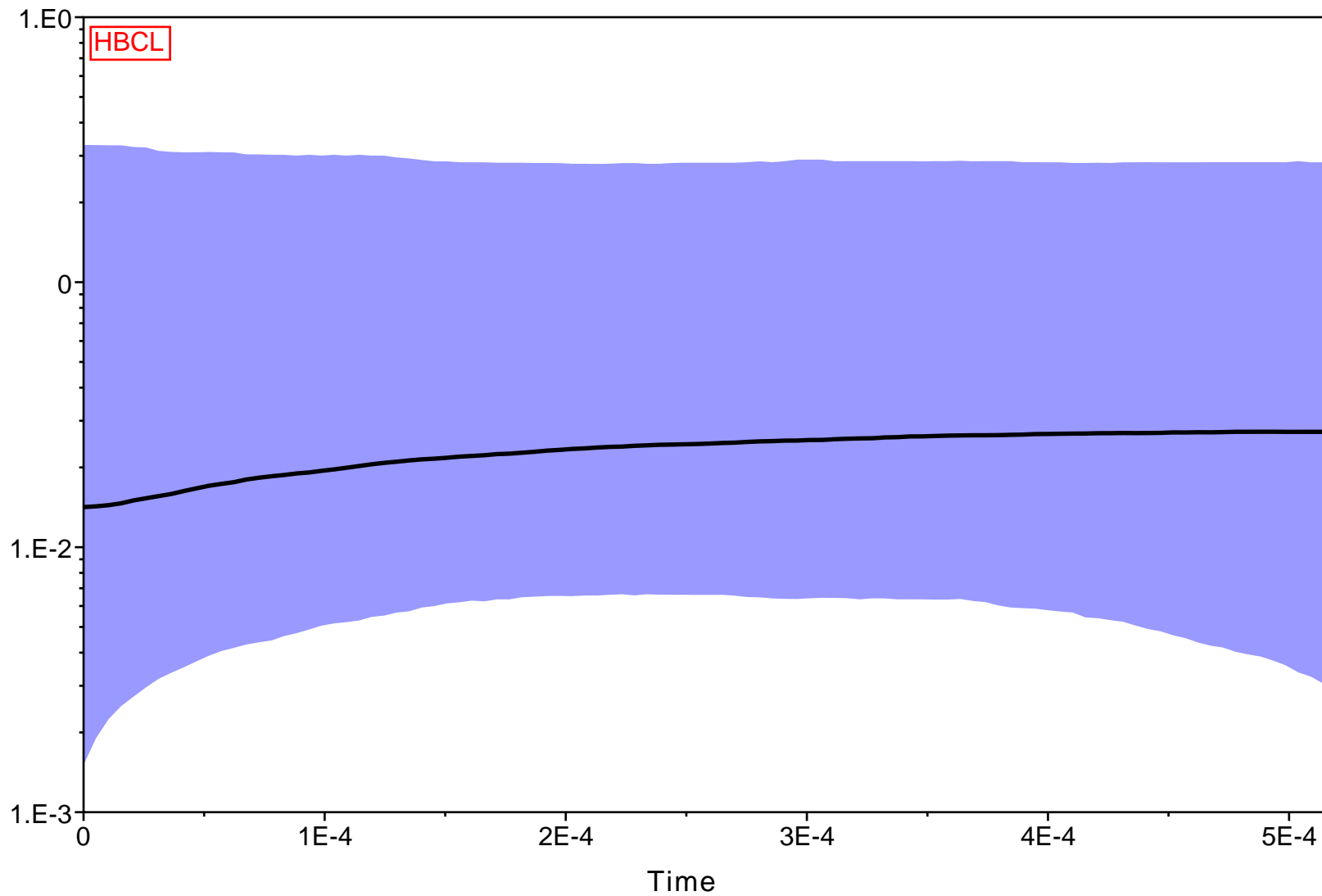

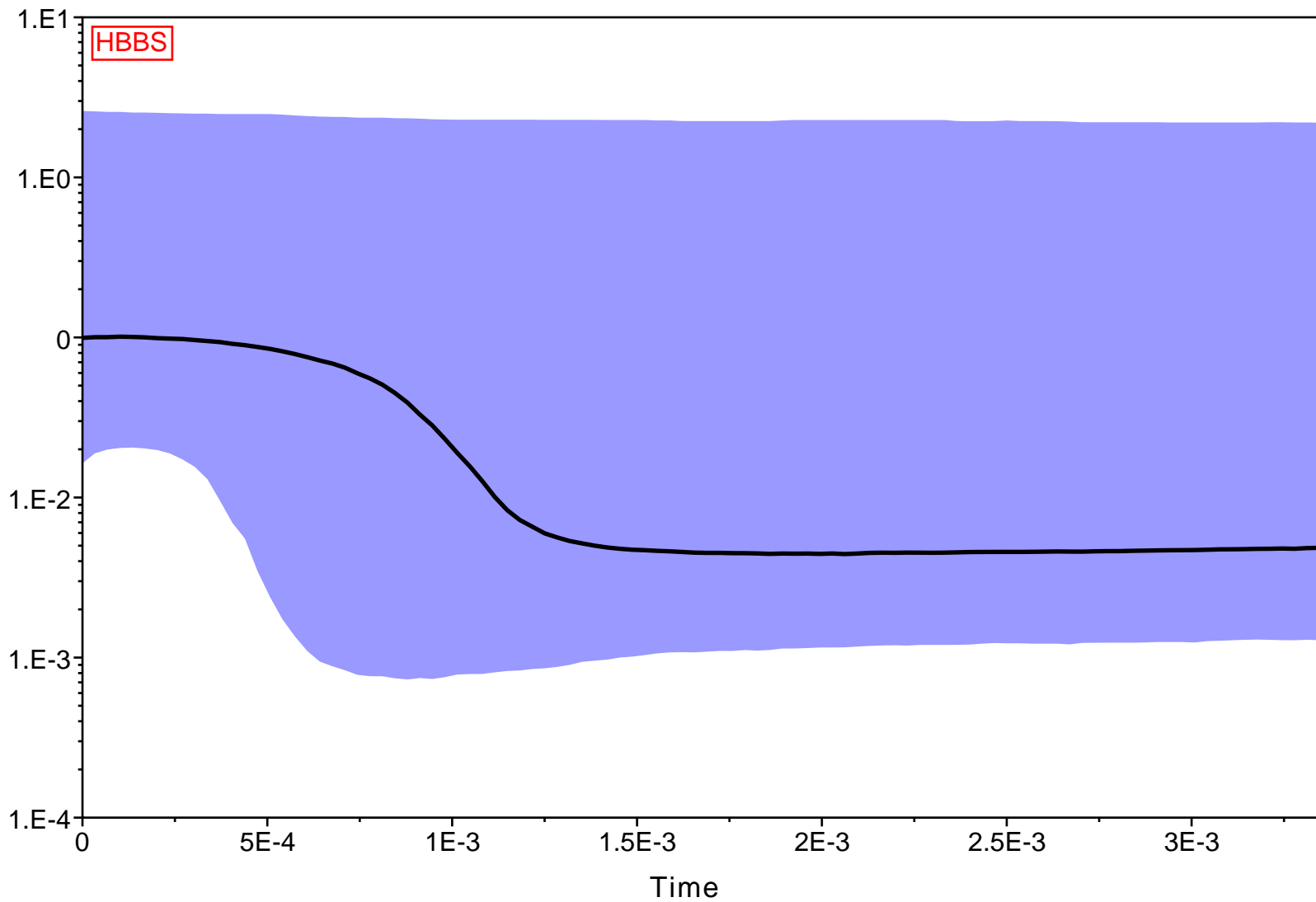

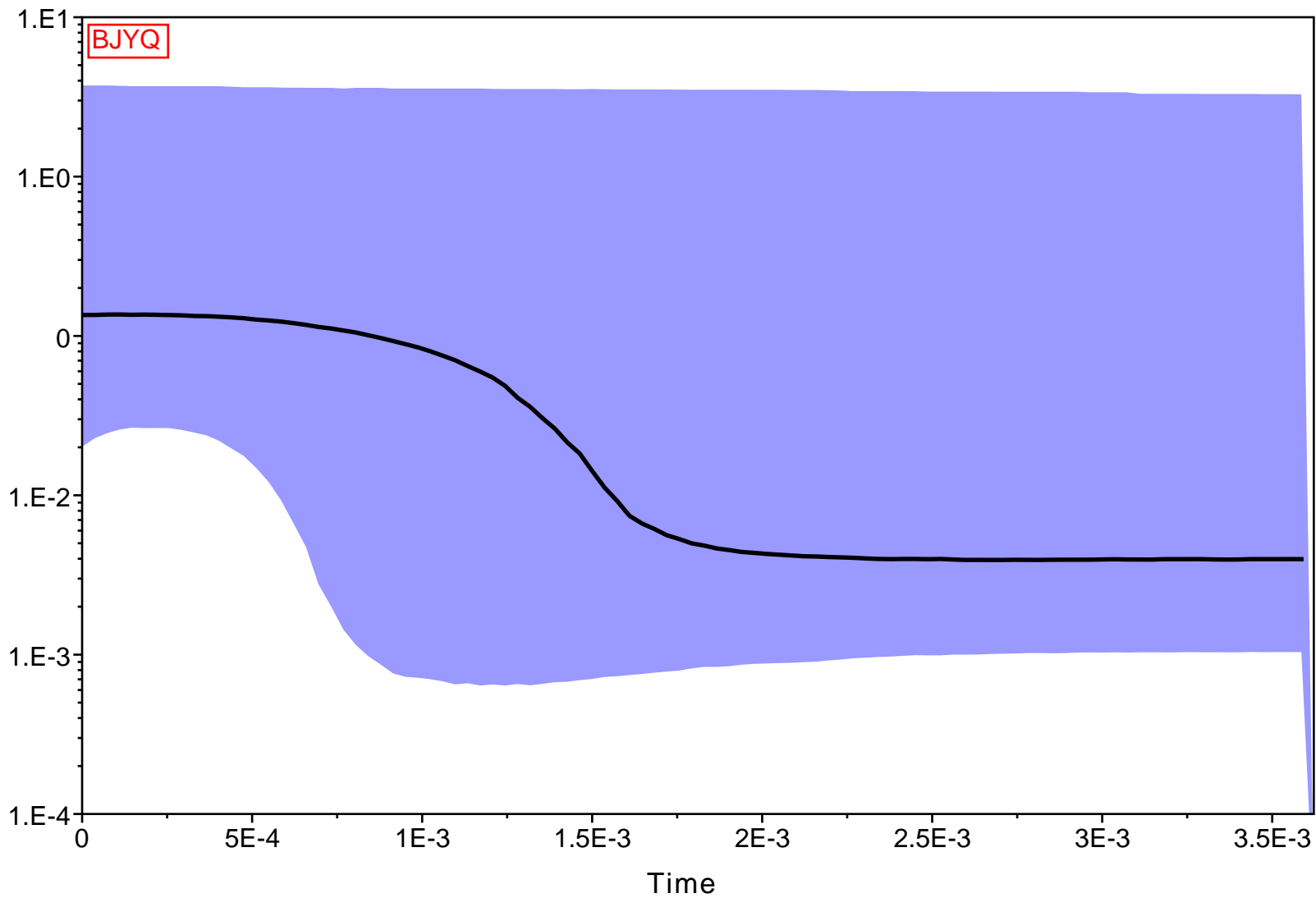

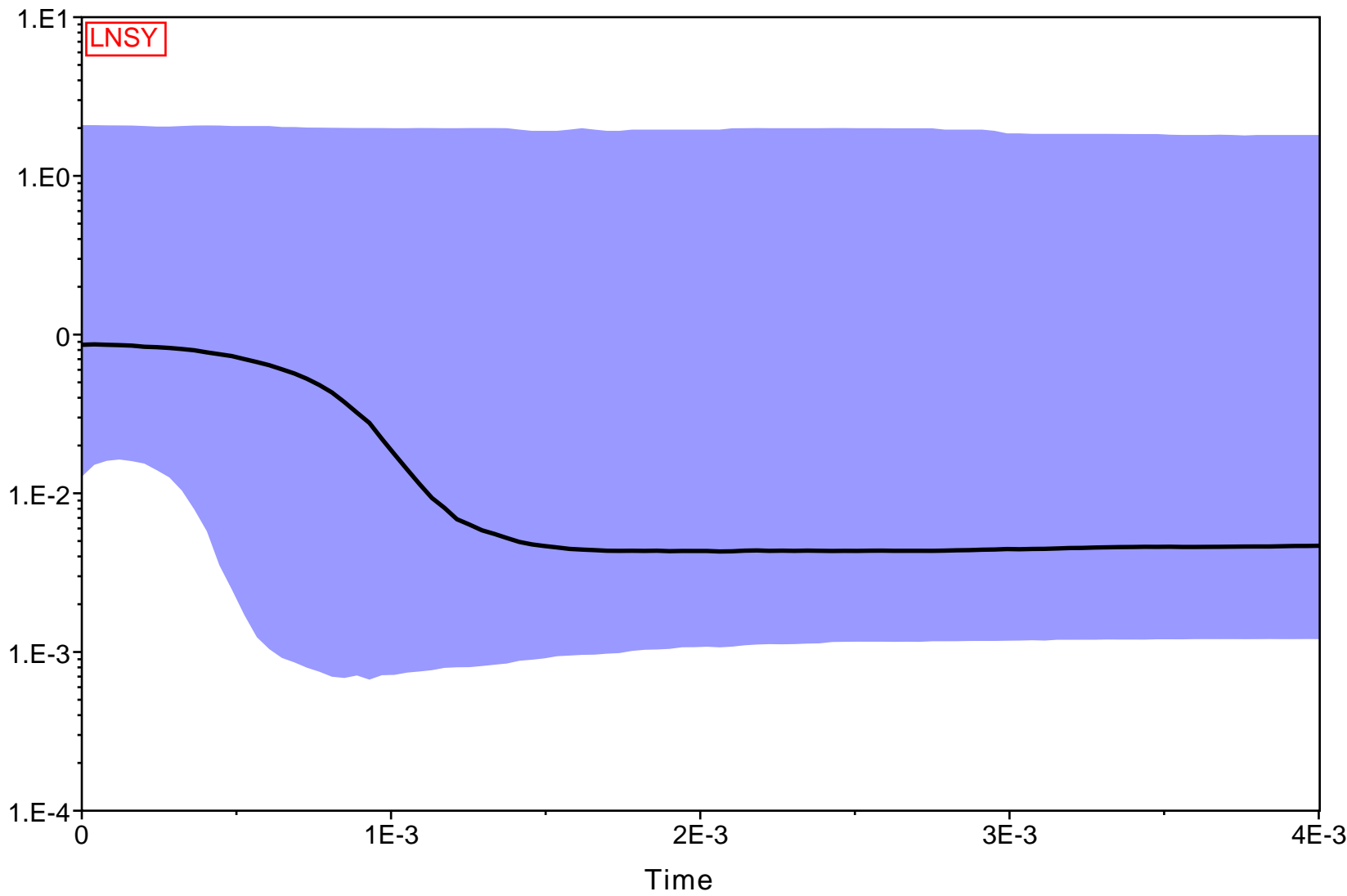

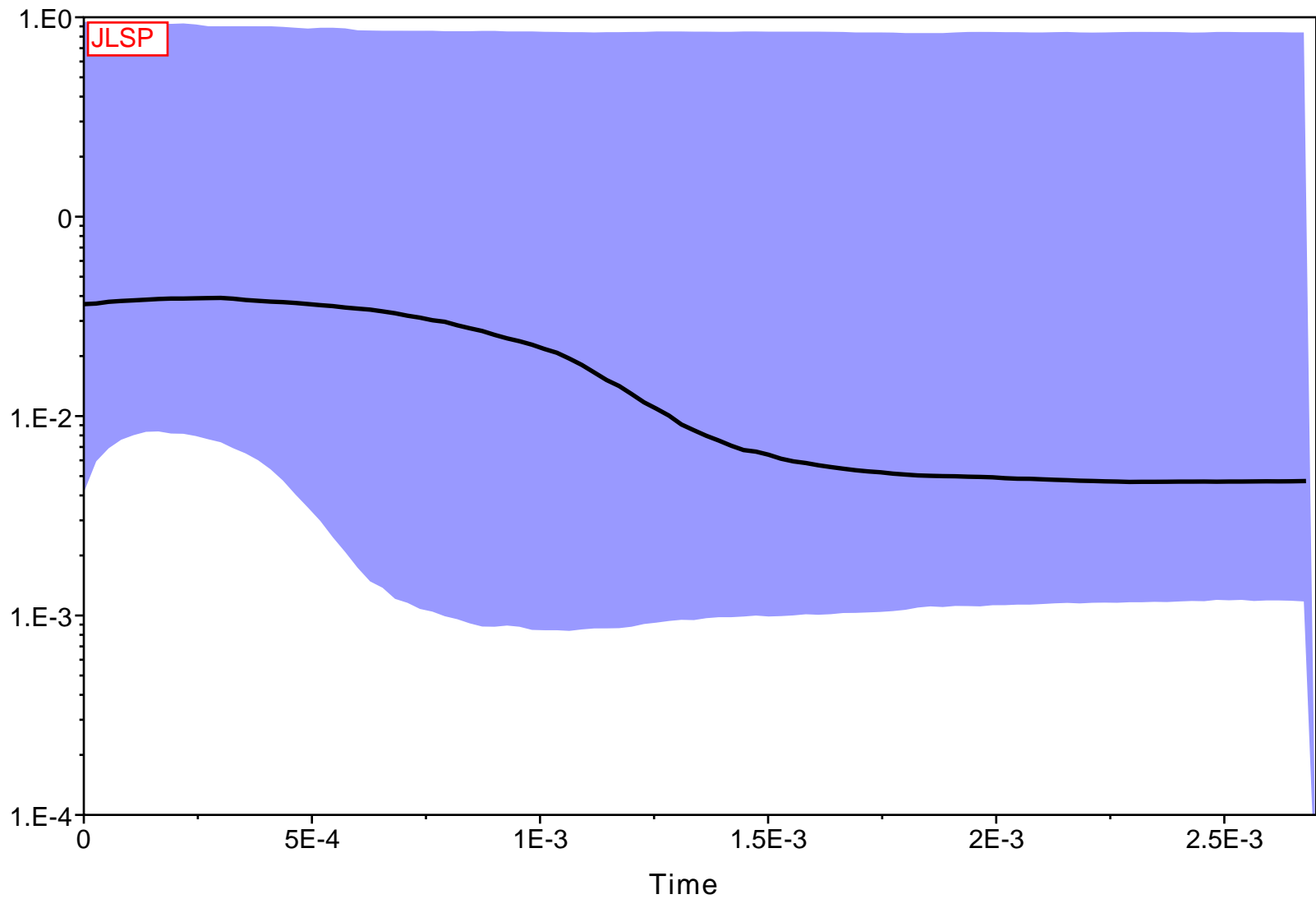

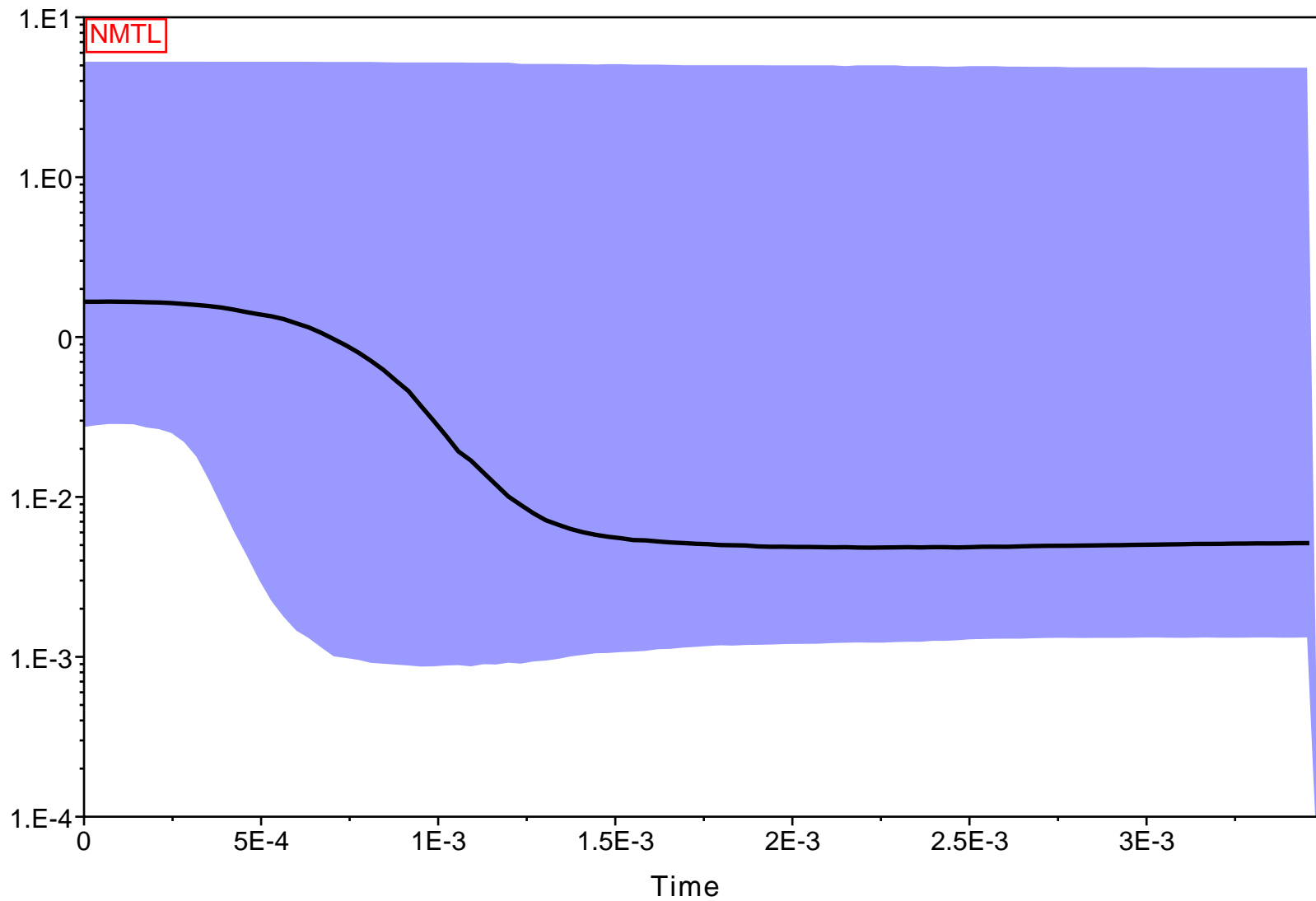

Supplement: Figure S5 — Mismatch distribution of the combined genes of cox1 , atp8 , atp6 and nad5 in the 27 populations of the Plutella xylostella from China using the ARLEQUIN suite, version 3.5 (PDF) [file pone.0059654.s005.pdf]
