# Supplementary material for: Genetic Structure and Demographic History Reveal Migration of the Diamondback Moth Plutella xylostella (Lepidoptera: Plutellidae) from the Southern to Northern Regions of China
Source: PLoS One. 2013 Apr 2;8(4):e59654. doi: 10.1371/journal.pone.0059654 (PMC3614937; doi:10.1371/journal.pone.0059654)
Supplement: Figure S6 — Bayesian Skyline Plot of the 27 populations of the Plutella xylostella from China based on the combined genes of cox1 , atp8 , atp6 and nad5 . (PDF) [file pone.0059654.s006.pdf]

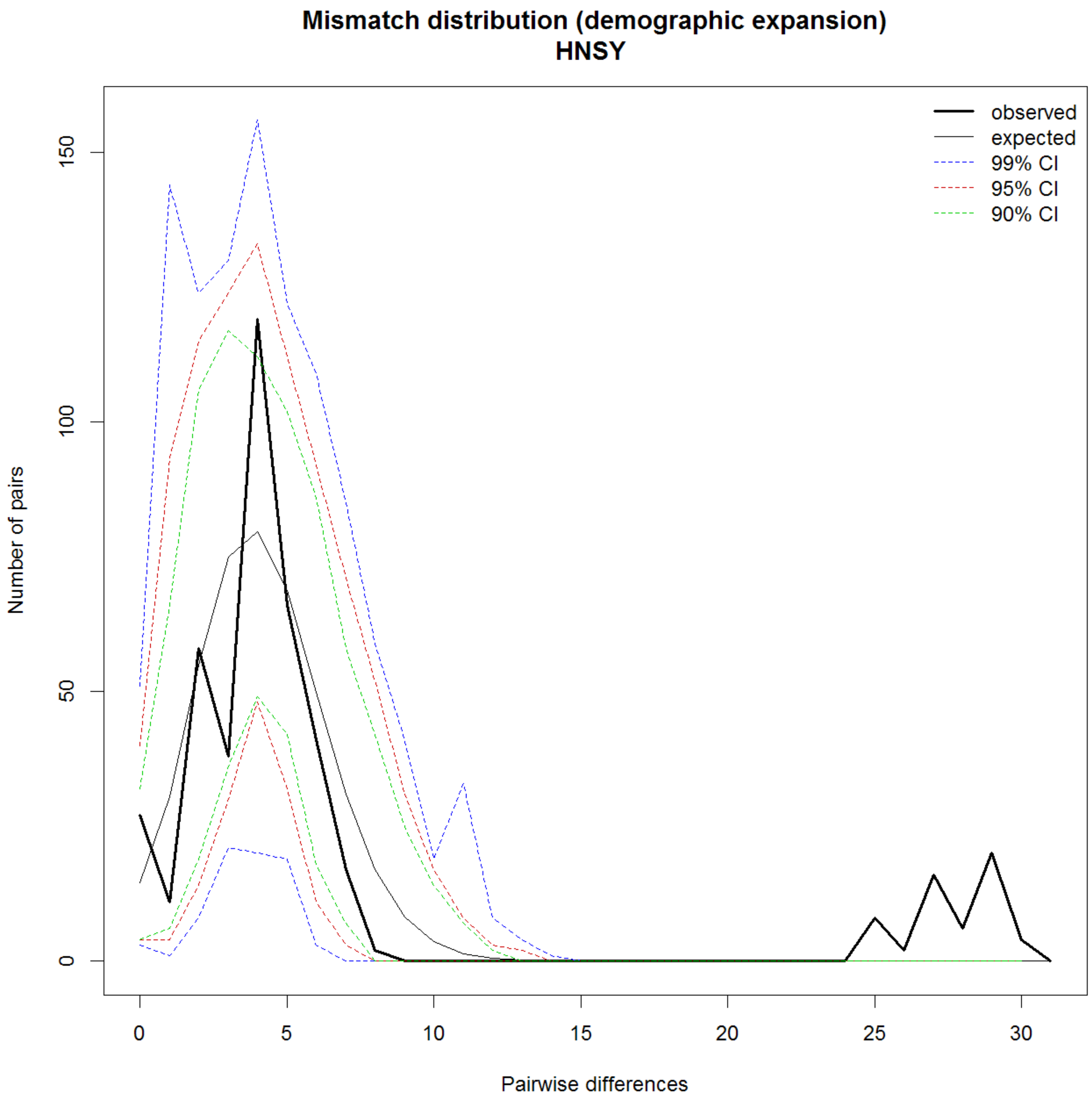
Mismatch distribution (demographic expansion)
HNSY
observed
expected
99% CI
95% CI
90% CI
Number of pairs
Pairwise differences
0
50
100
150
0
5
10
15
20
25
30

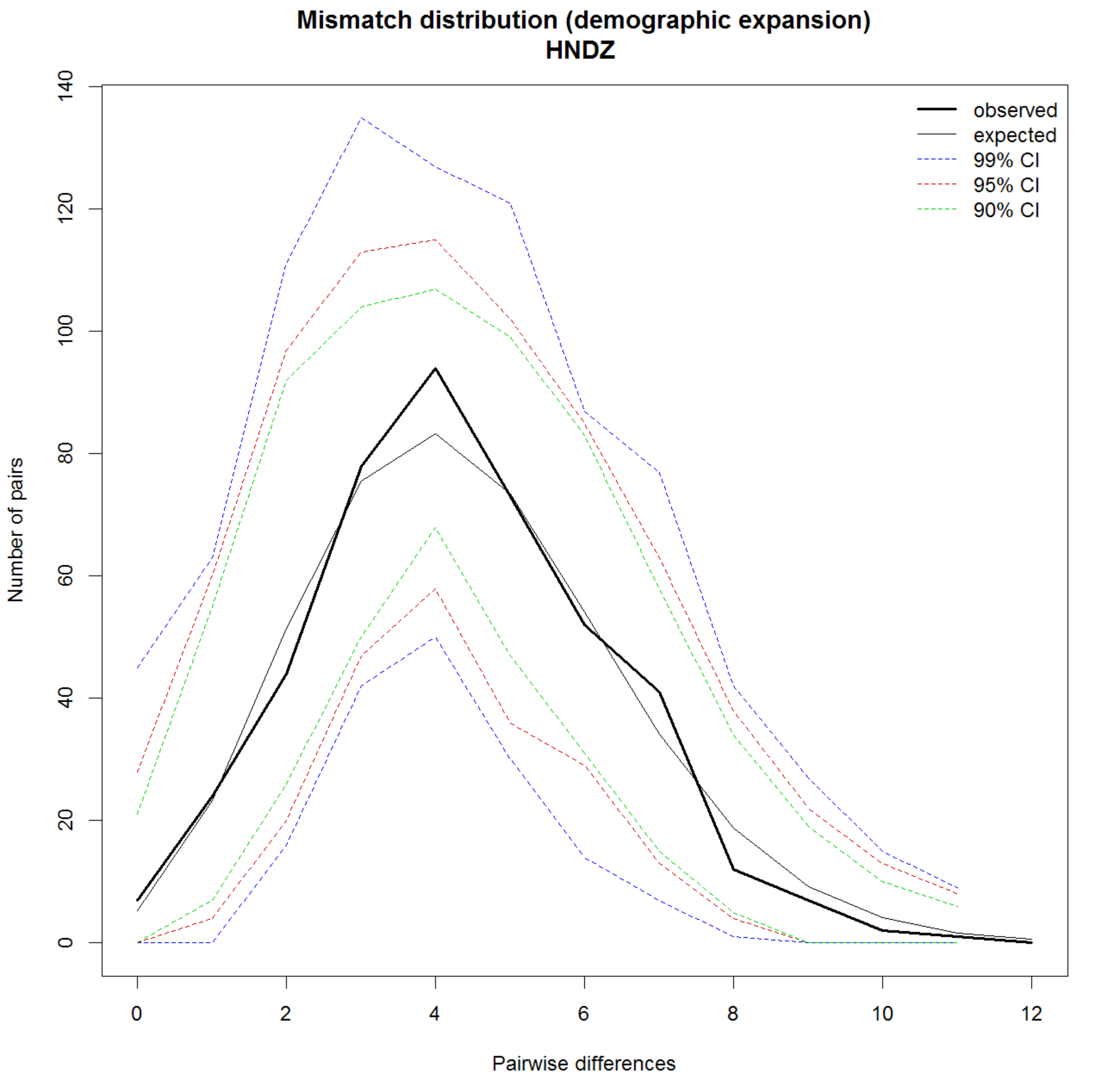

Mismatch distribution (demographic expansion)
HNDZ
observed
expected
99% CI
95% CI
90% CI
Number of pairs
0
20
40
60
80
100
120
140
0
2
4
6
8
10
12
Pairwise differences

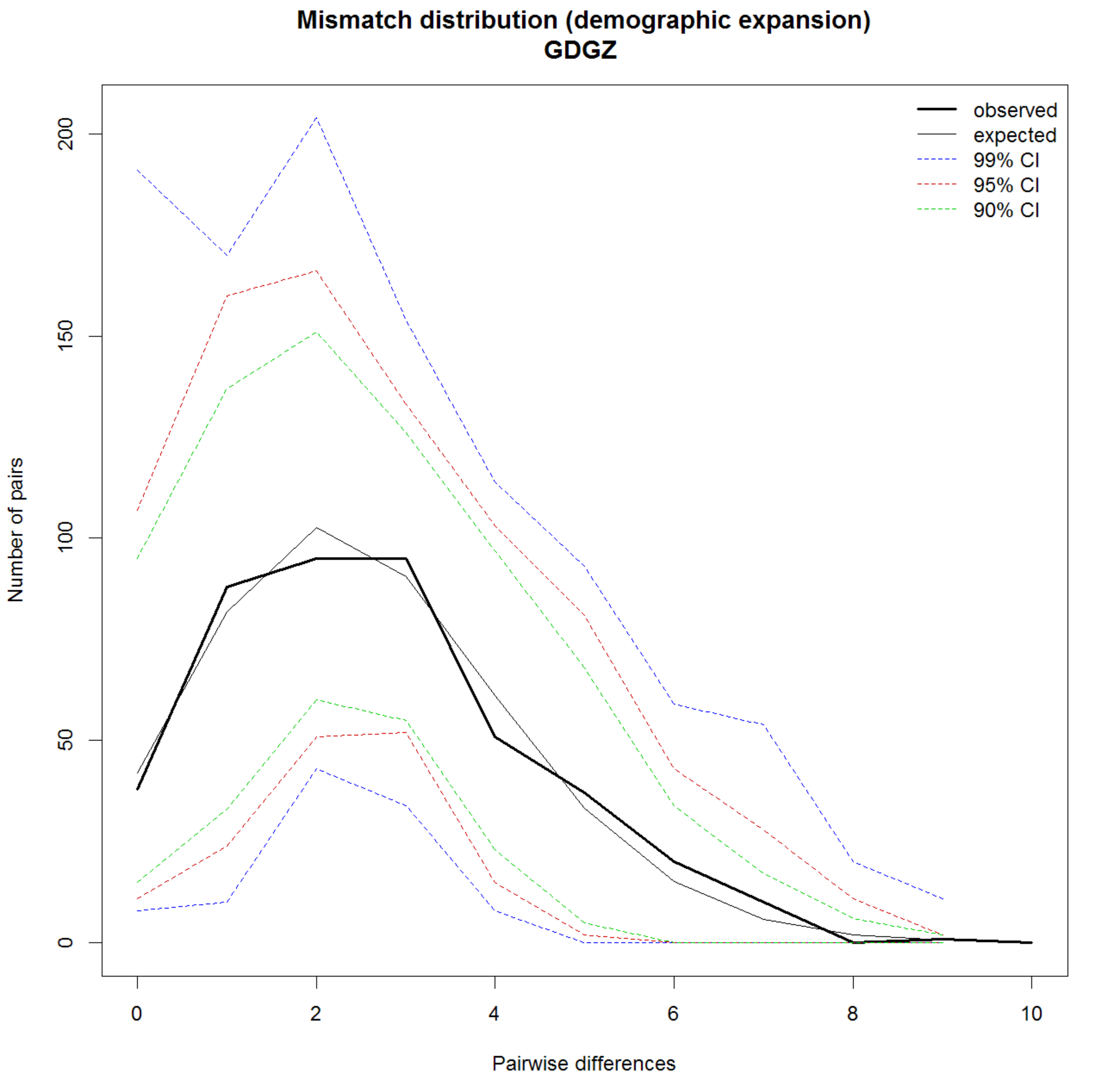
Mismatch distribution (demographic expansion)
GDGZ
observed
expected
99% CI
95% CI
90% CI
Number of pairs
Pairwise differences
0
50
100
150
200
0
2
4
6
8
10

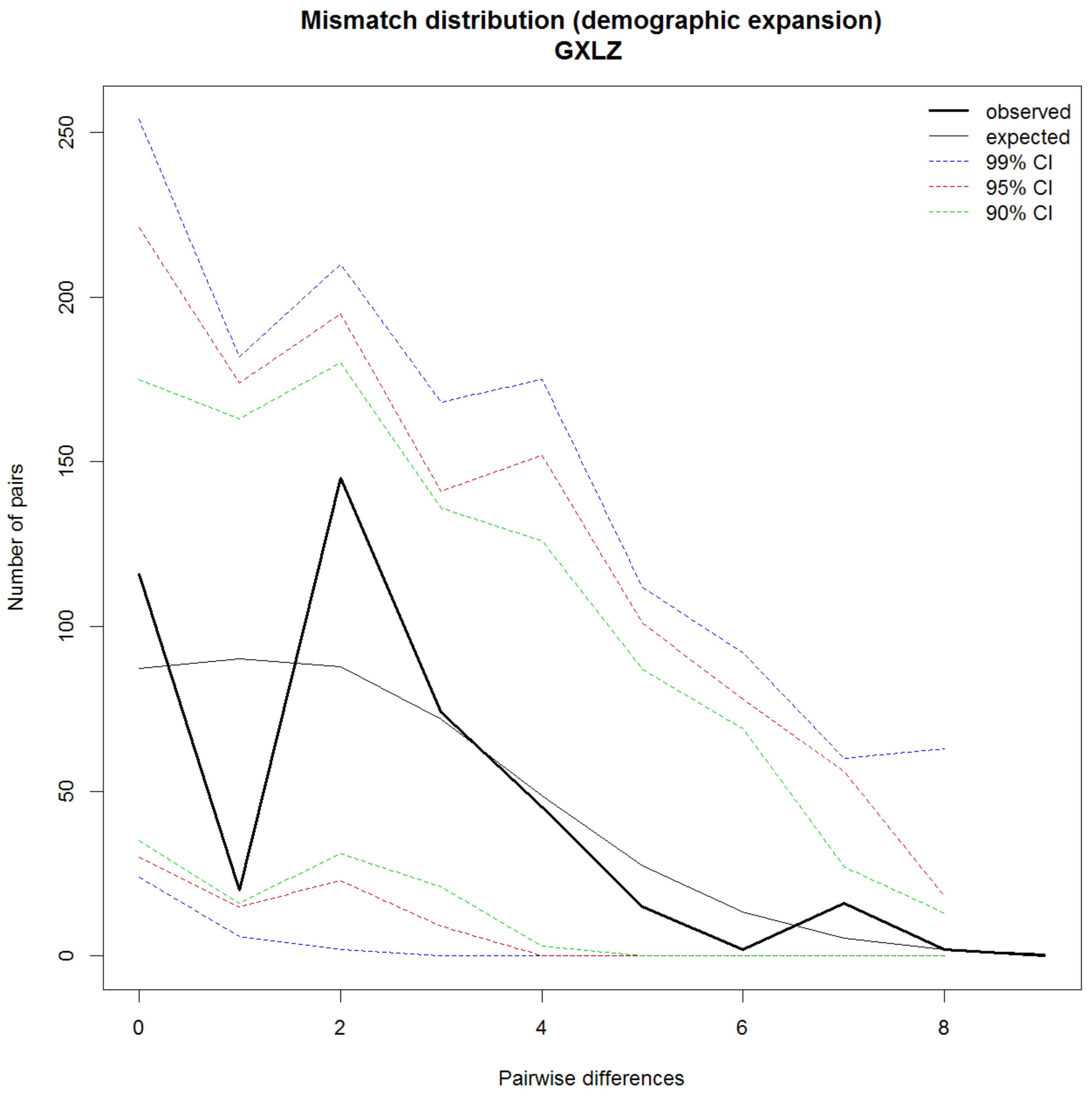

Mismatch distribution (demographic expansion)
GXLZ
observed
expected
99% CI
95% CI
90% CI
Number of pairs
Pairwise differences
0
50
100
150
200
250
0
2
4
6
8

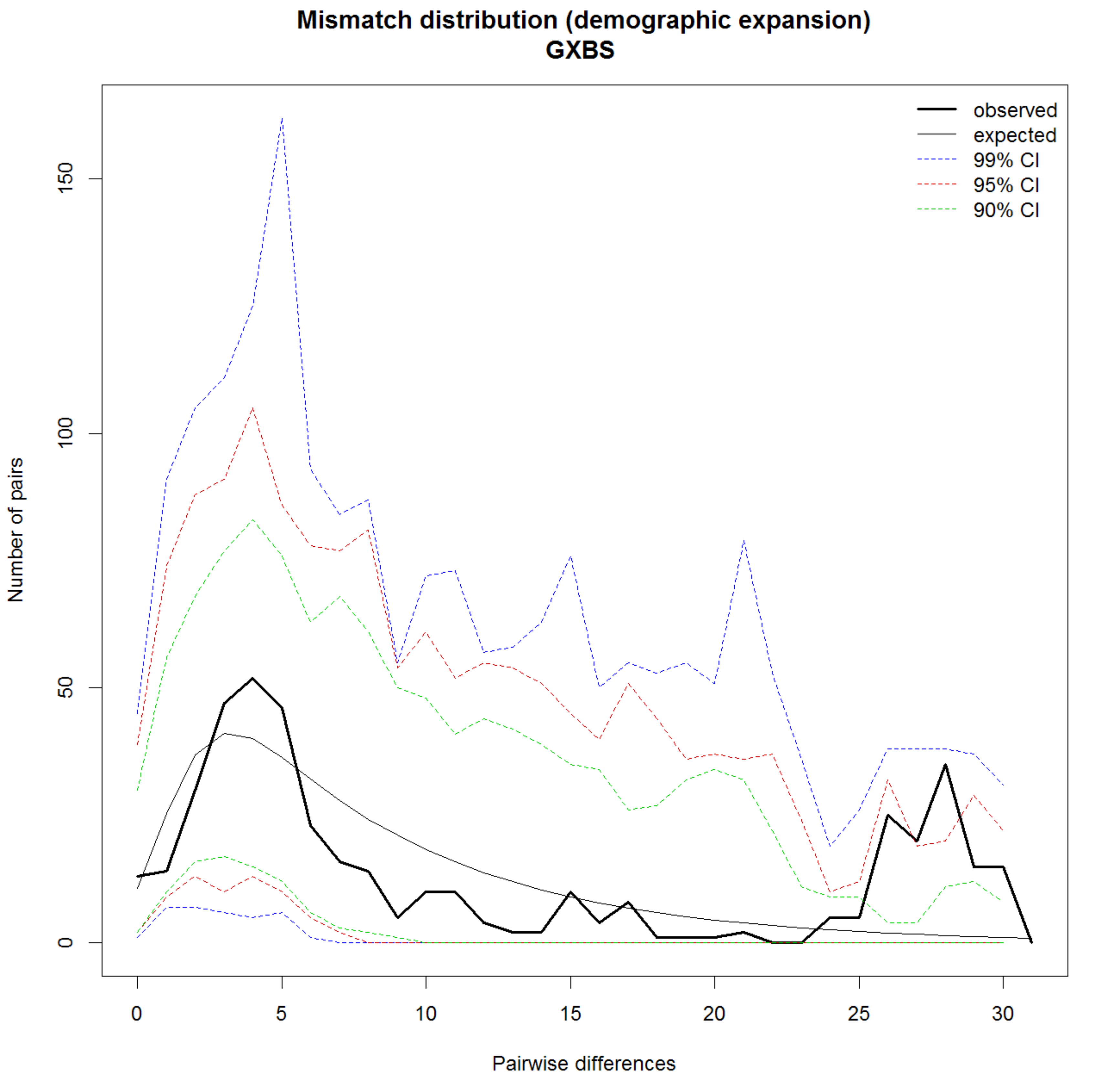

Mismatch distribution (demographic expansion)
GXBS
observed
expected
99% CI
95% CI
90% CI
Number of pairs
Pairwise differences
0
50
100
150
0
5
10
15
20
25
30

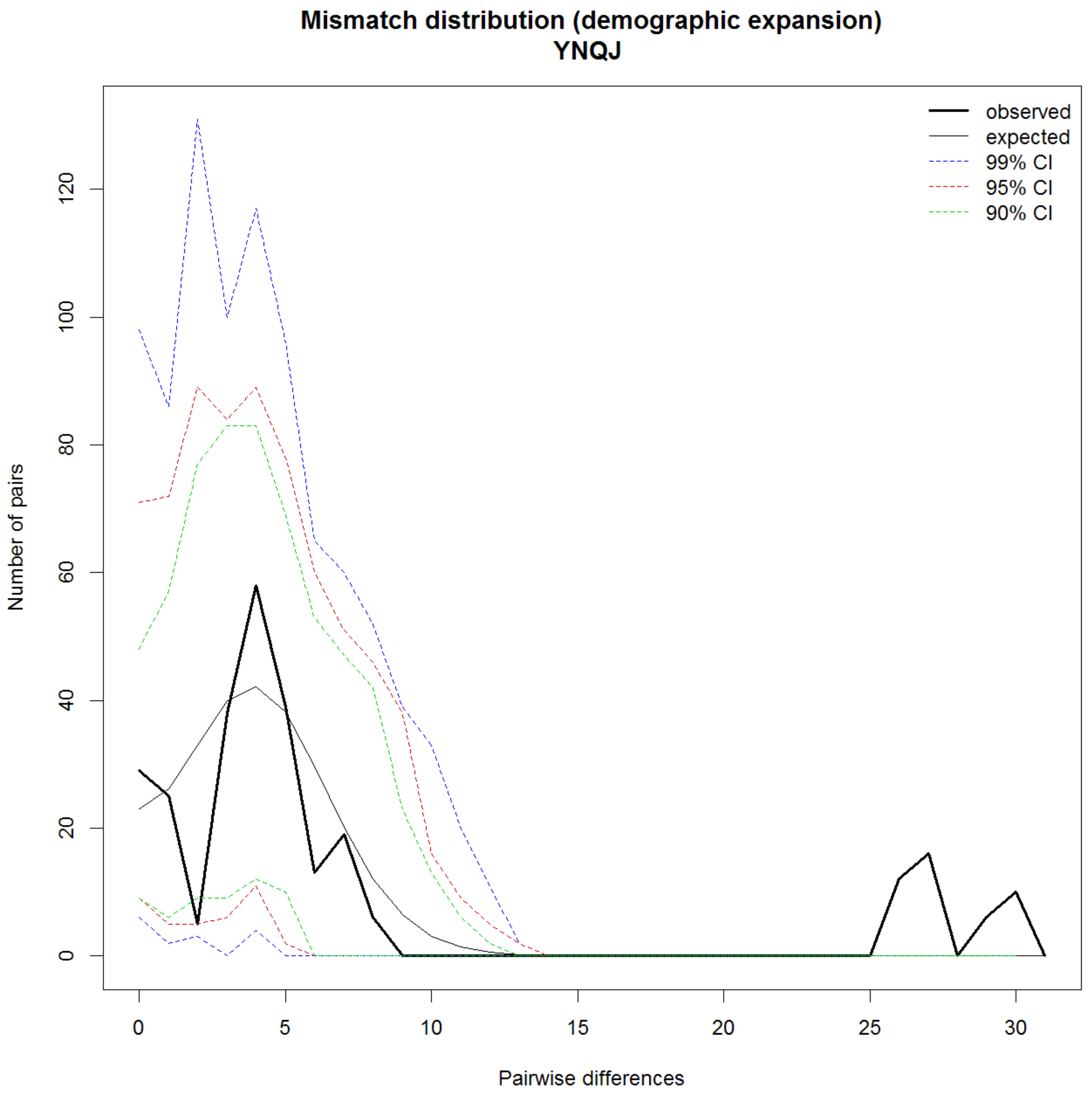
Mismatch distribution (demographic expansion)
YNQJ
observed
expected
99% CI
95% CI
90% CI
Number of pairs
0
20
40
60
80
100
120
0
5
10
15
20
25
30
Pairwise differences

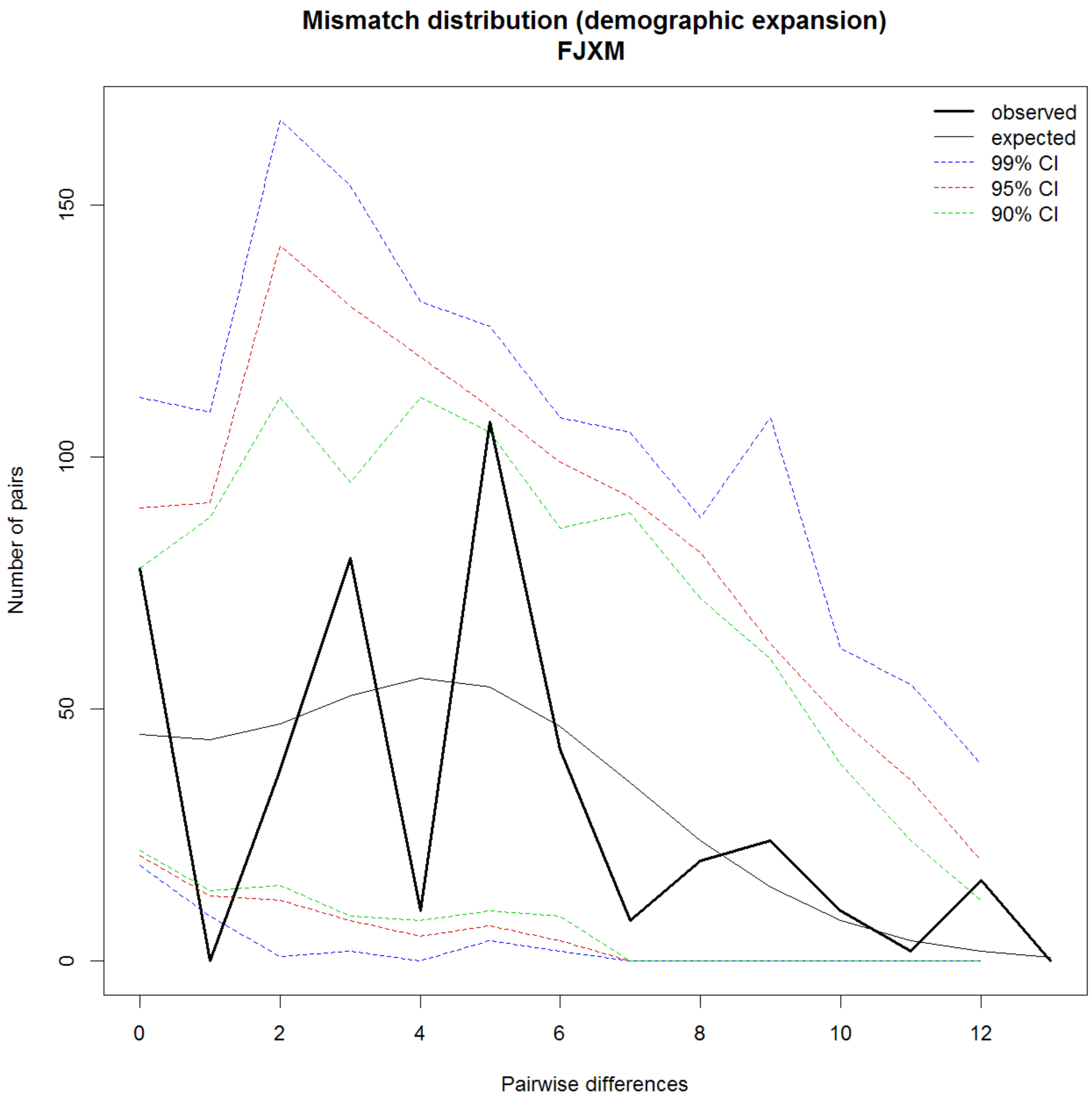
Mismatch distribution (demographic expansion)
FJXM
observed
expected
99% CI
95% CI
90% CI
Number of pairs
0
50
100
150
0
2
4
6
8
10
12
Pairwise differences

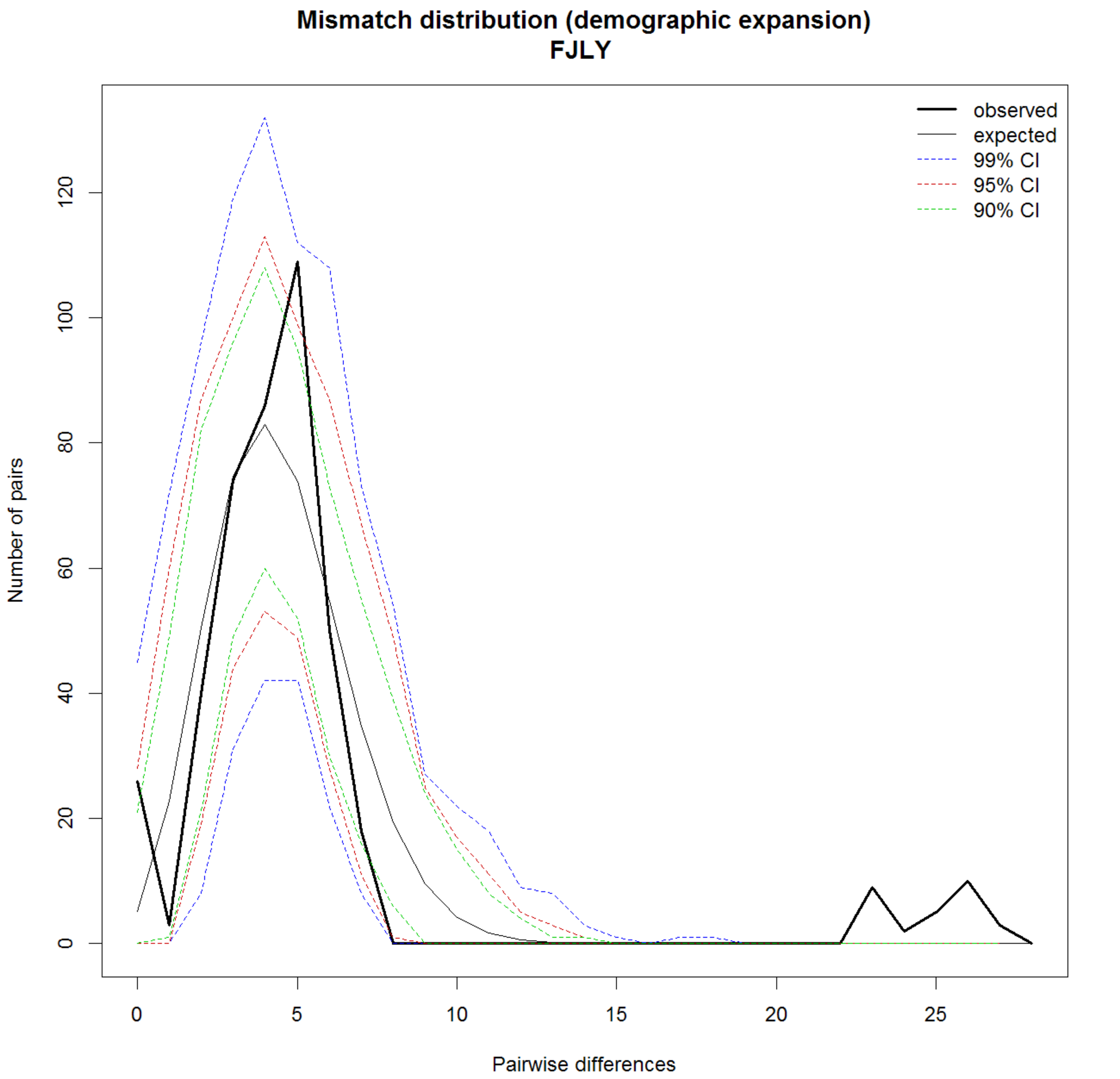
Mismatch distribution (demographic expansion)
FJLY
observed
expected
99% CI
95% CI
90% CI
Number of pairs
Pairwise differences
0
20
40
60
80
100
120
0
5
10
15
20
25

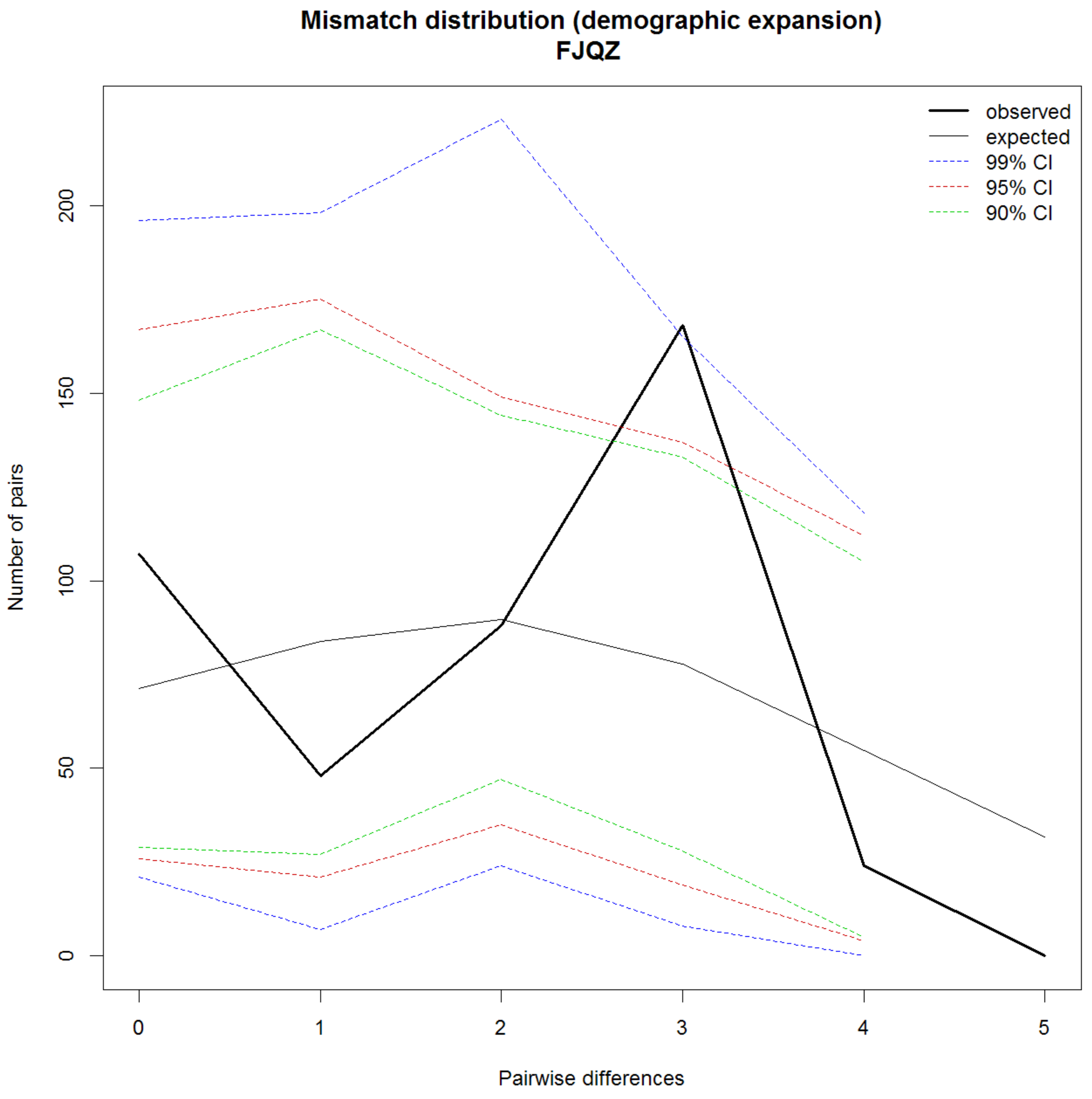
Mismatch distribution (demographic expansion)
FJQZ
observed
expected
99% CI
95% CI
90% CI
Number of pairs
0
50
100
150
200
0
1
2
3
4
5
Pairwise differences

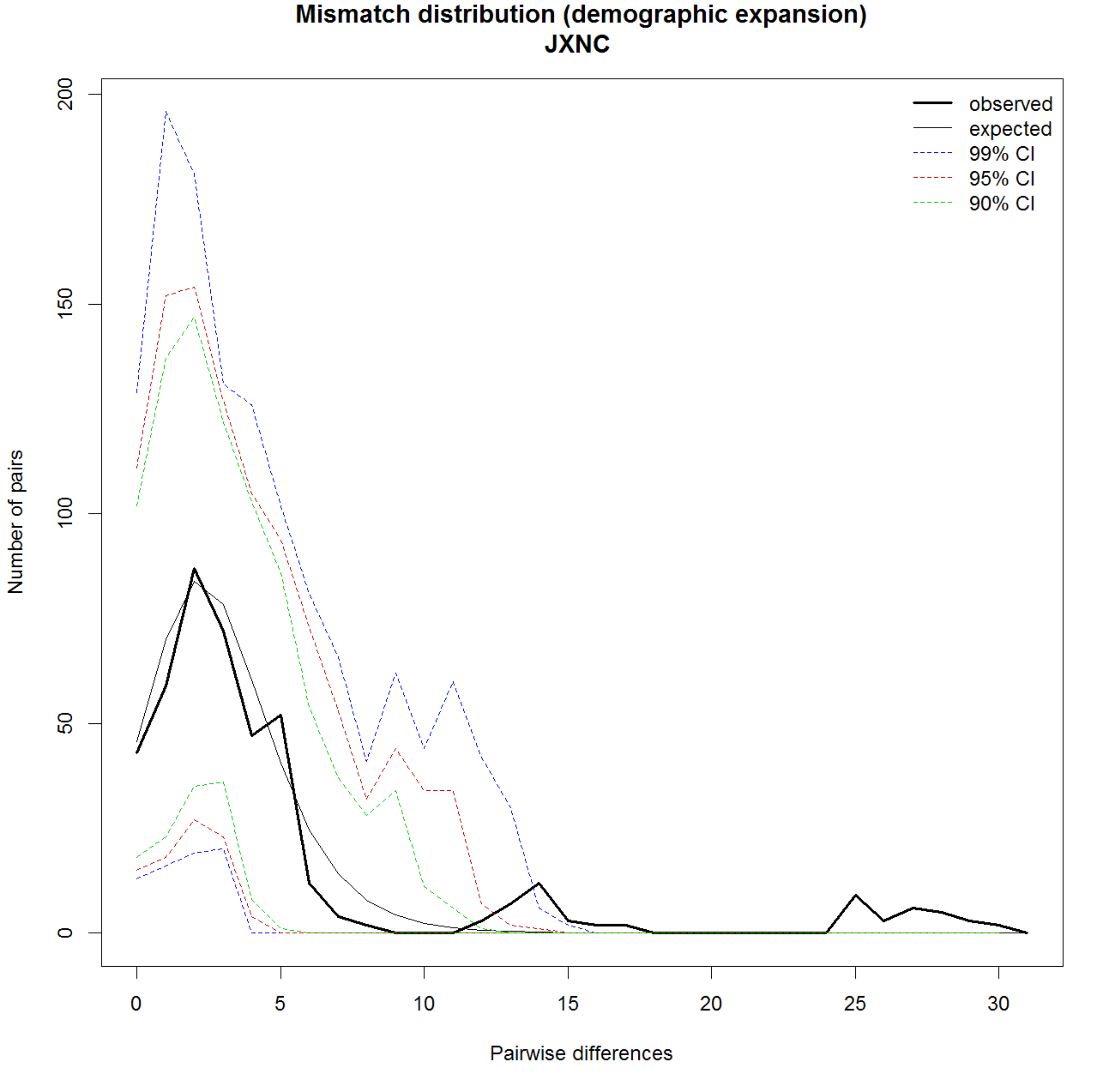
Mismatch distribution (demographic expansion)
JXNC
observed
expected
99% CI
95% CI
90% CI
Number of pairs
Pairwise differences
0
50
100
150
200
0
5
10
15
20
25
30

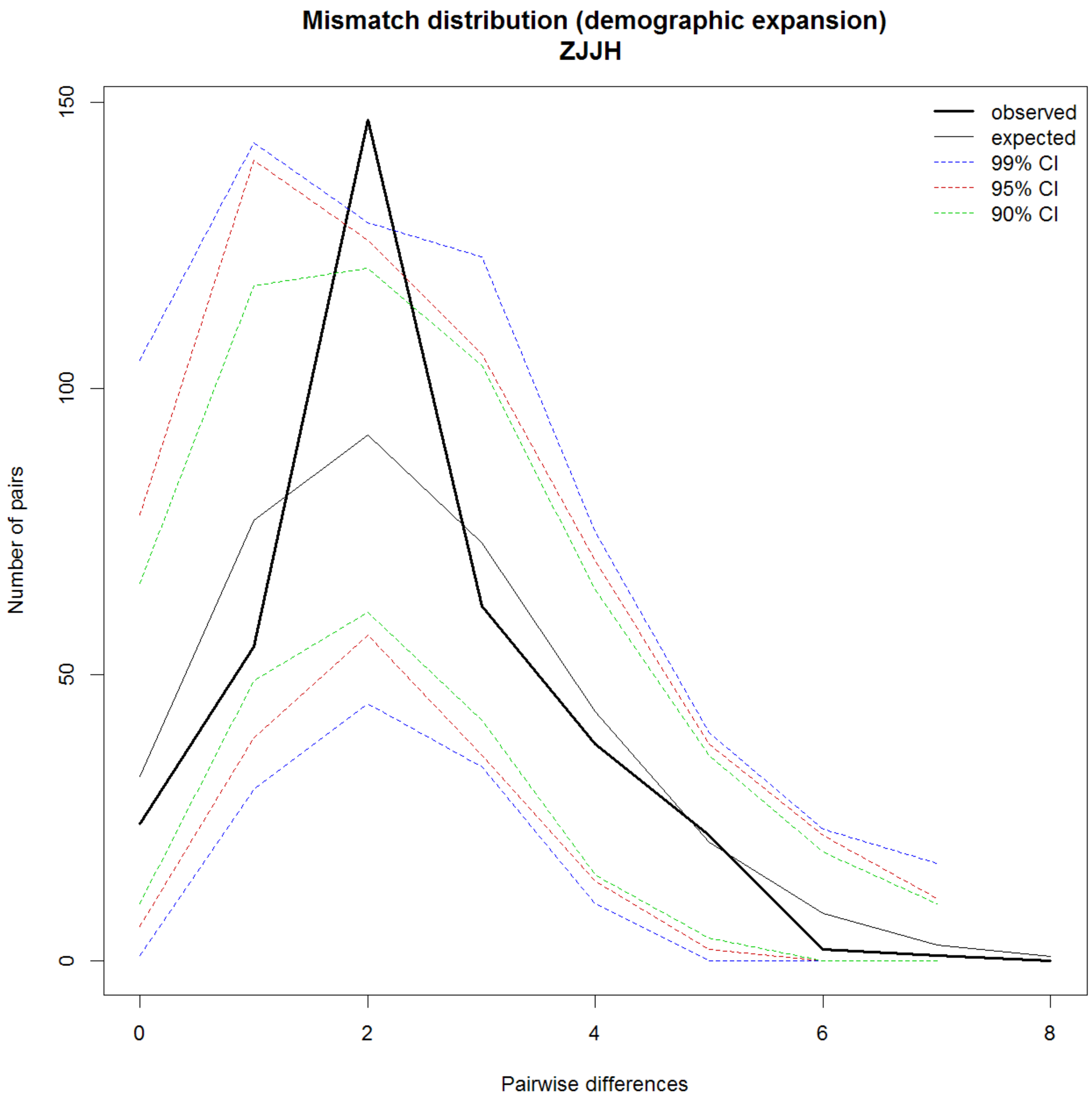
Mismatch distribution (demographic expansion)
ZJJH
observed
expected
99% CI
95% CI
90% CI
Number of pairs
Pairwise differences
0
50
100
150
0
2
4
6
8

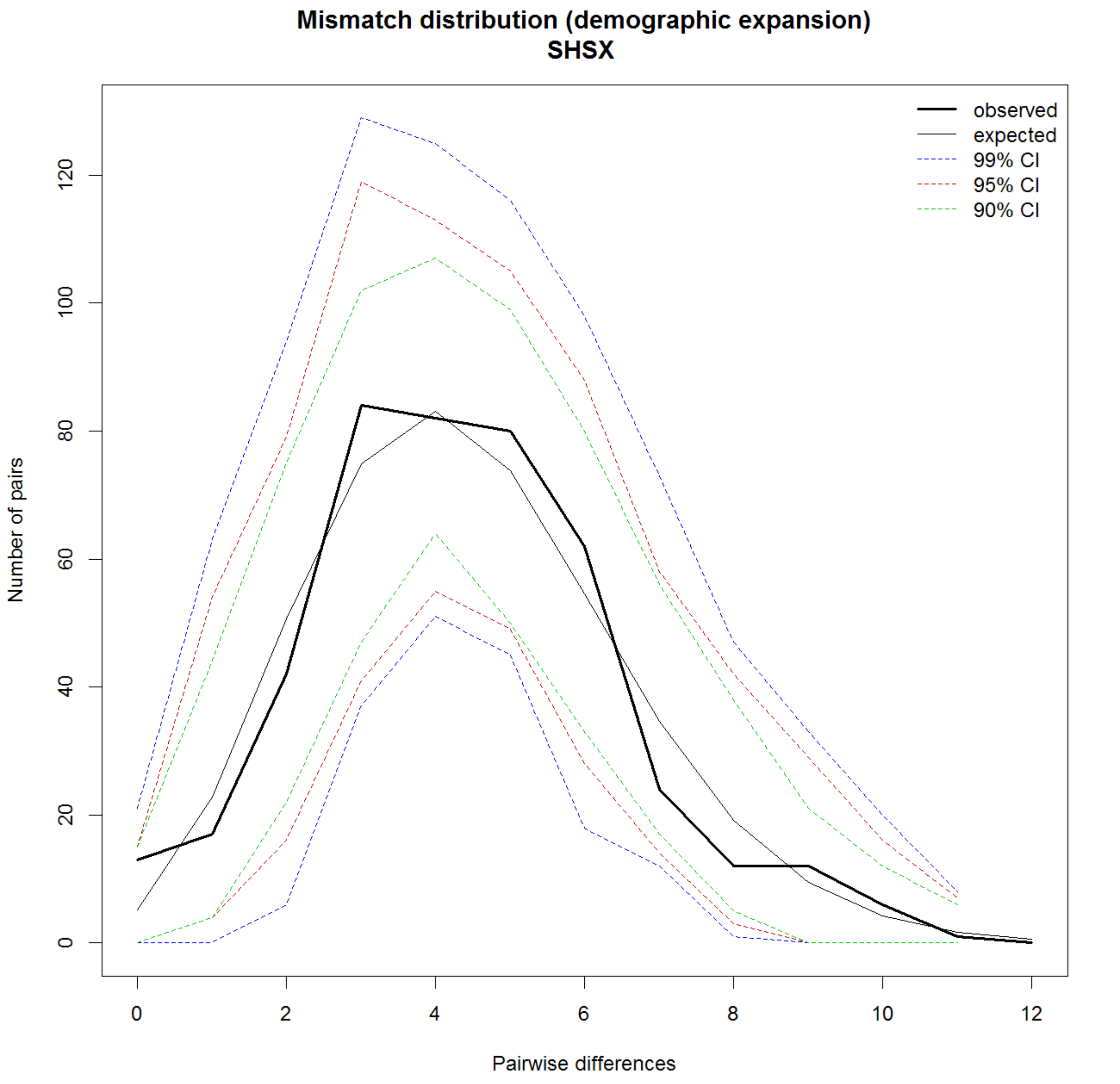
Mismatch distribution (demographic expansion)
SHSX
observed
expected
99% CI
95% CI
90% CI
Number of pairs
Pairwise differences
0
20
40
60
80
100
120
0
2
4
6
8
10
12

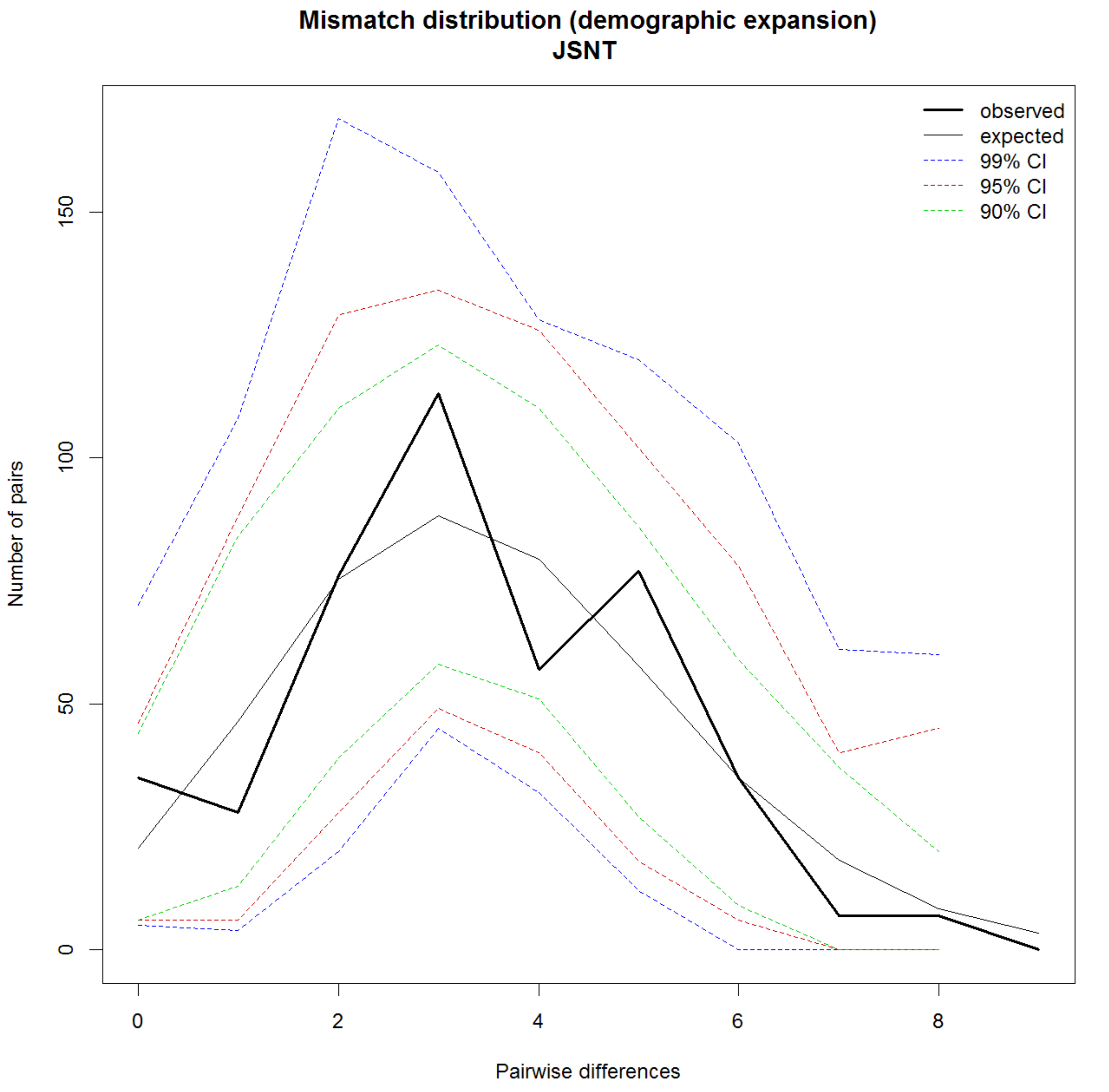
Mismatch distribution (demographic expansion)
JSNT
observed
expected
99% CI
95% CI
90% CI
Number of pairs
0
50
100
150
0
2
4
6
8
Pairwise differences

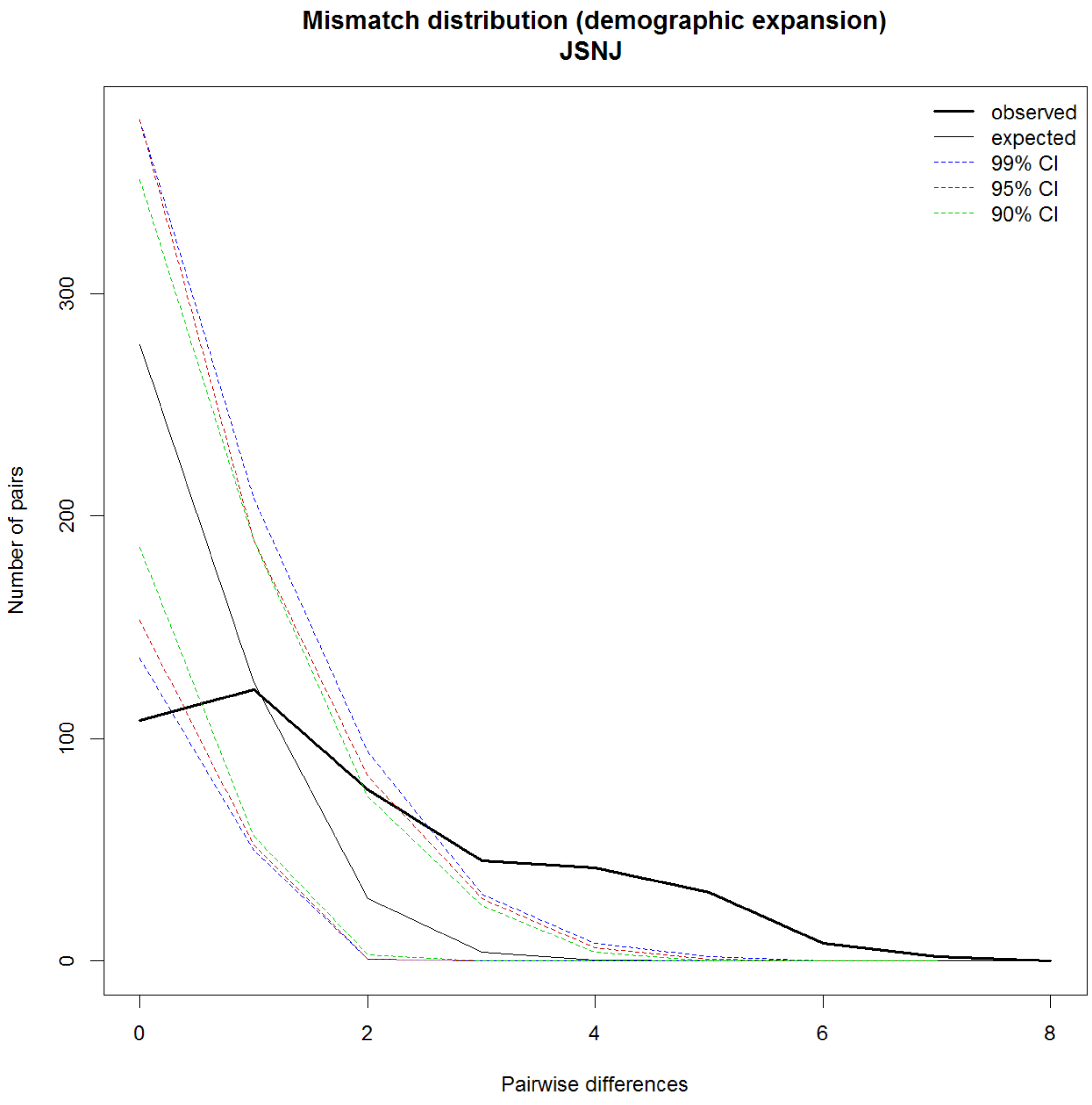
Mismatch distribution (demographic expansion)
JSNJ
observed
expected
99% CI
95% CI
90% CI
Number of pairs
0
100
200
300
Pairwise differences
0
2
4
6
8

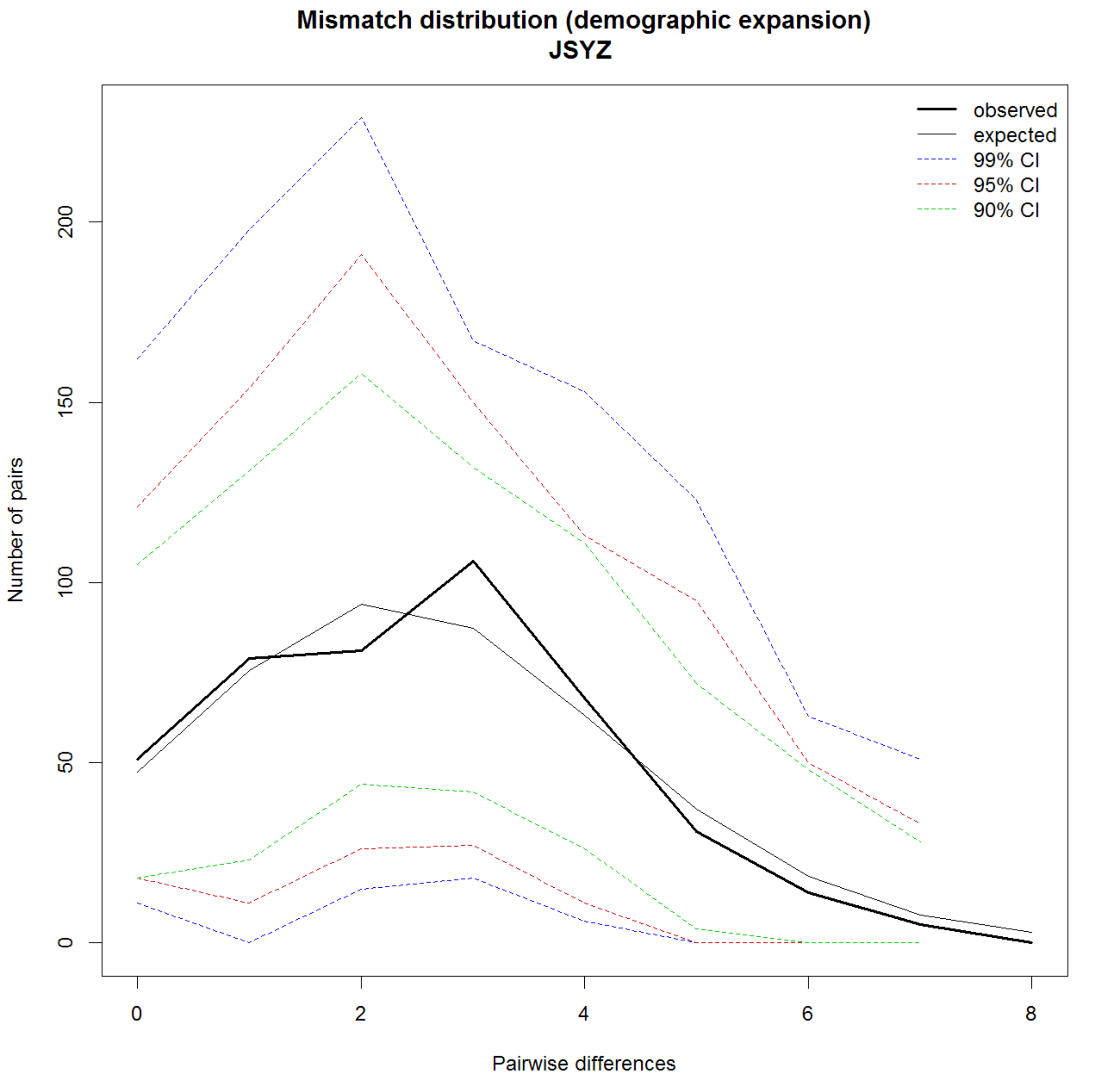

Mismatch distribution (demographic expansion)
JSYZ
observed
expected
99% CI
95% CI
90% CI
Number of pairs
0
50
100
150
200
0
2
4
6
8
Pairwise differences

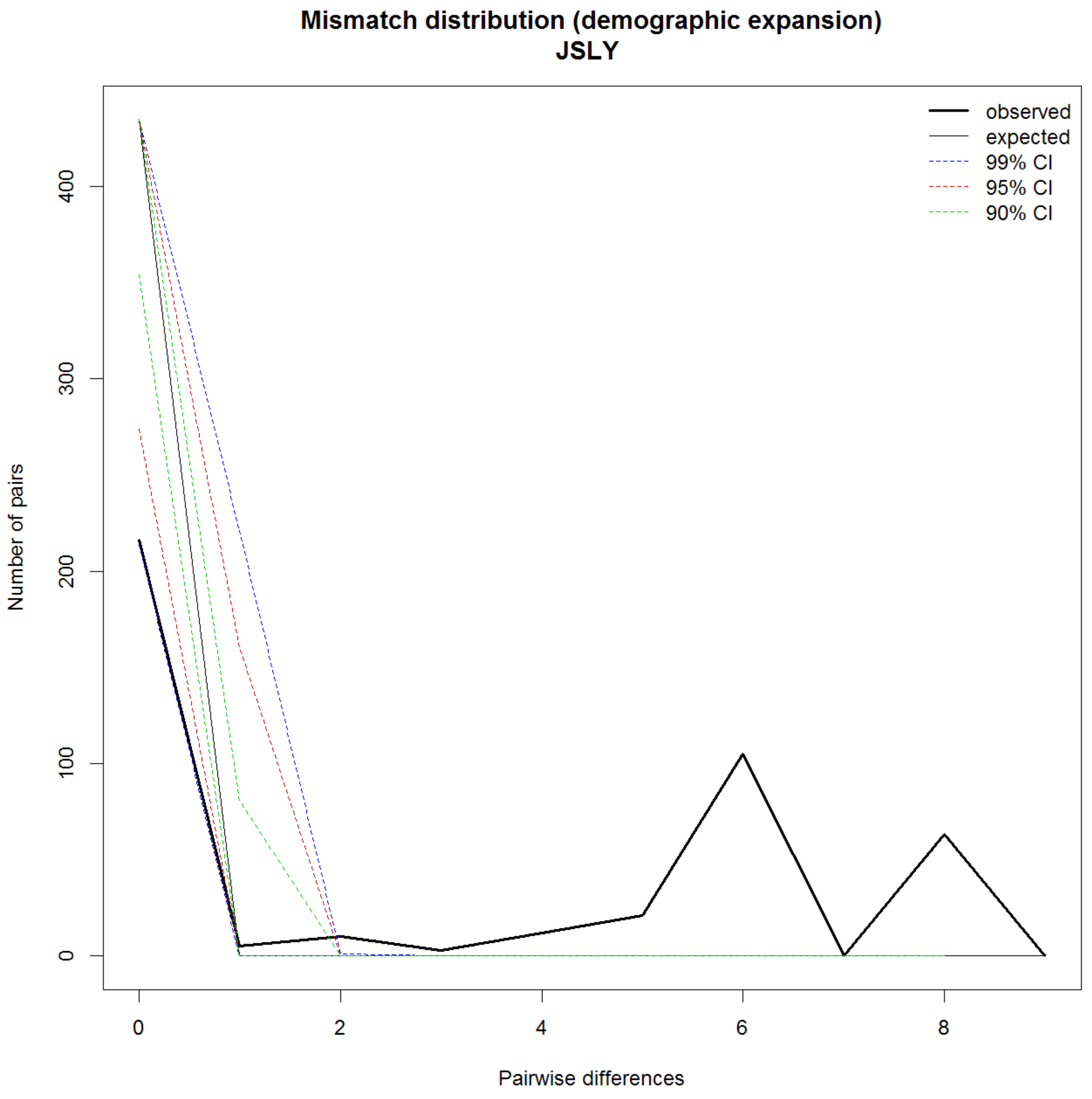
Mismatch distribution (demographic expansion)
JSLY
observed
expected
99% CI
95% CI
90% CI
Number of pairs
Pairwise differences
0
100
200
300
400
0
2
4
6
8

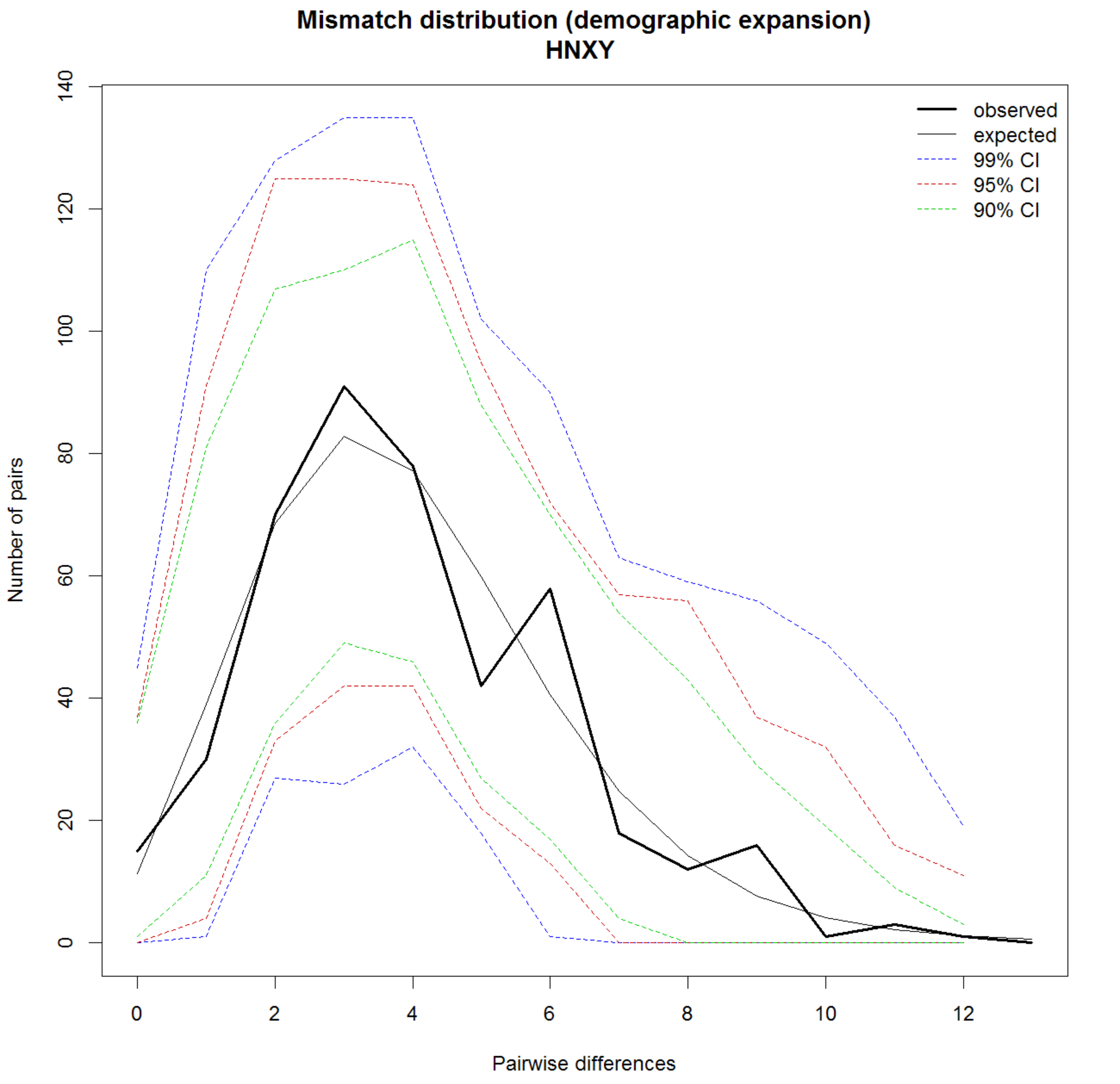
Mismatch distribution (demographic expansion)
HNXY
observed
expected
99% CI
95% CI
90% CI
Number of pairs
Pairwise differences
0
20
40
60
80
100
120
140
0
2
4
6
8
10
12

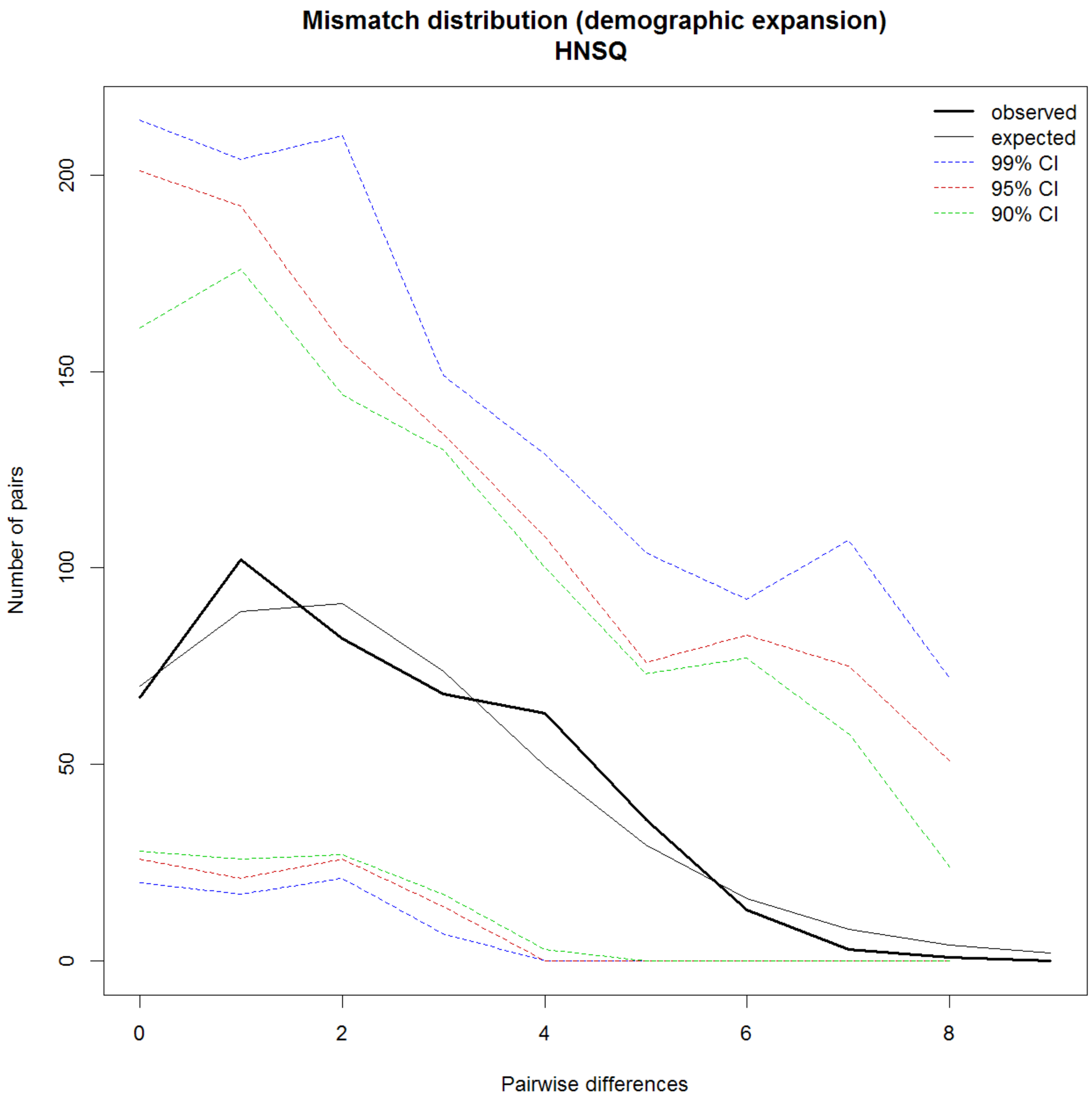

Mismatch distribution (demographic expansion)
HNSQ
observed
expected
99% CI
95% CI
90% CI
Number of pairs
Pairwise differences
0
50
100
150
200
0
2
4
6
8

Mismatch distribution (demographic expansion)
SDQD

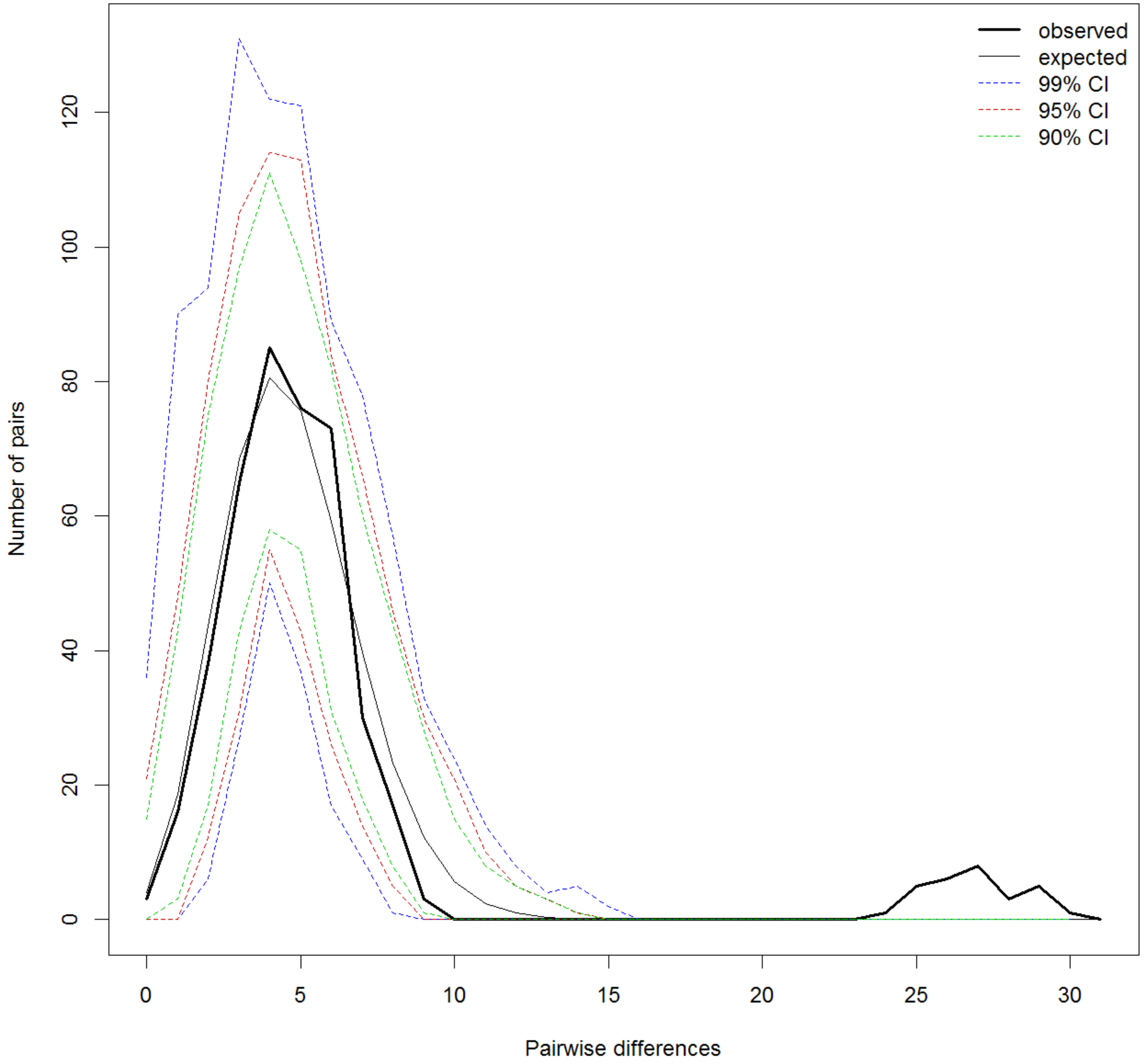

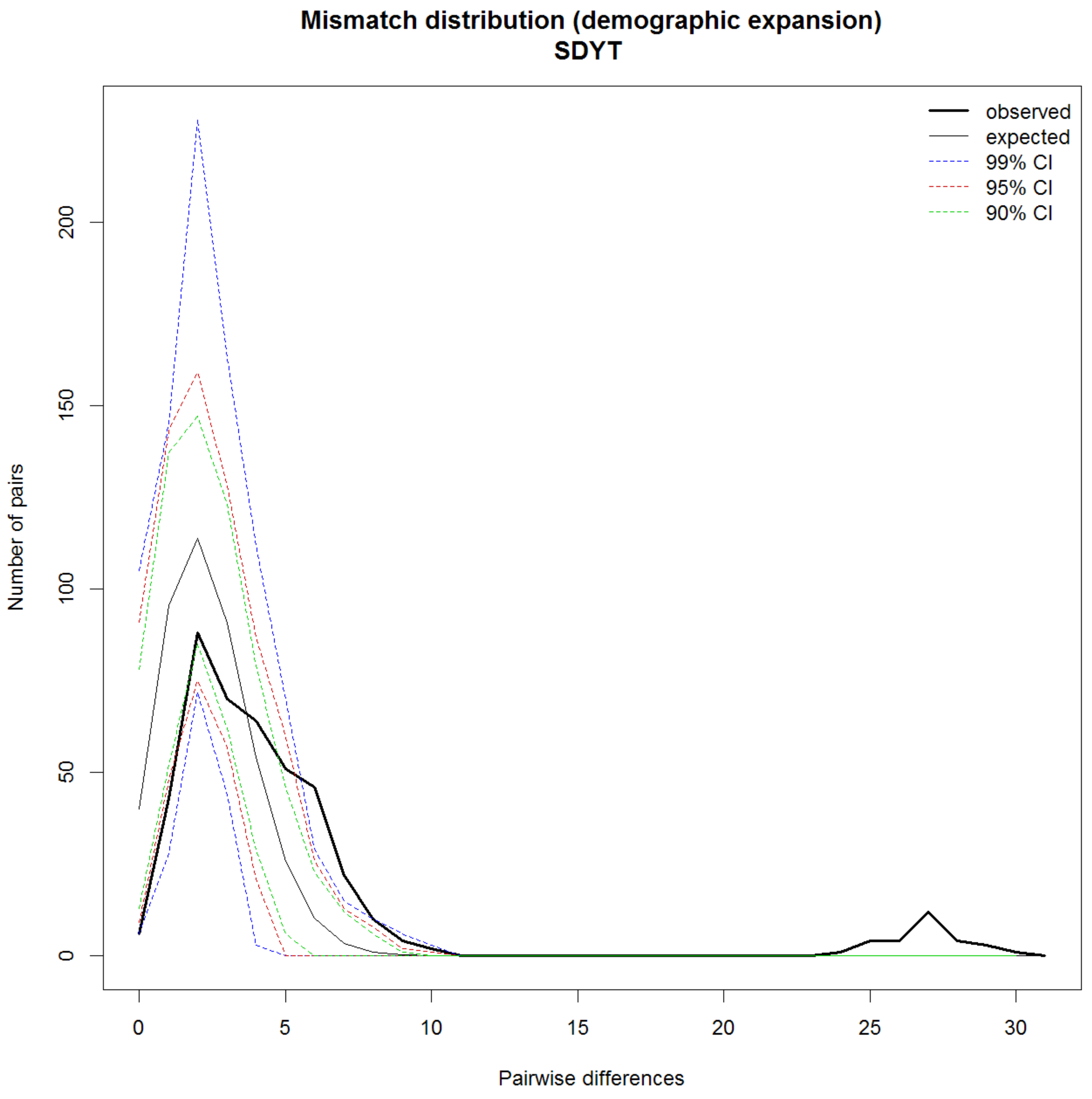

Mismatch distribution (demographic expansion)
SDYT
observed
expected
99% CI
95% CI
90% CI
Number of pairs
0
50
100
150
200
0
5
10
15
20
25
30
Pairwise differences

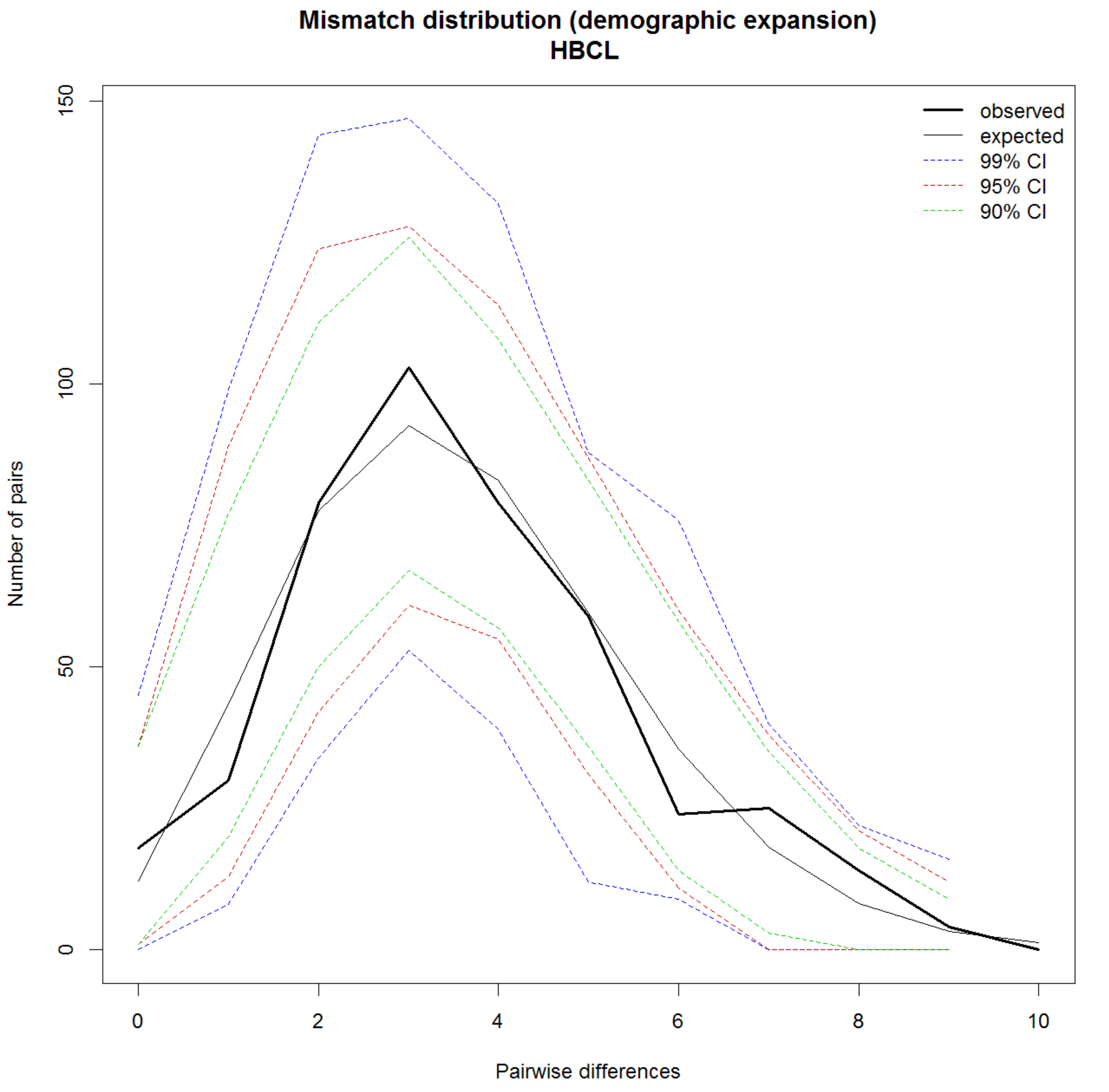

Mismatch distribution (demographic expansion)
HBCL
observed
expected
99% CI
95% CI
90% CI
Number of pairs
Pairwise differences
0
50
100
150
0
2
4
6
8
10

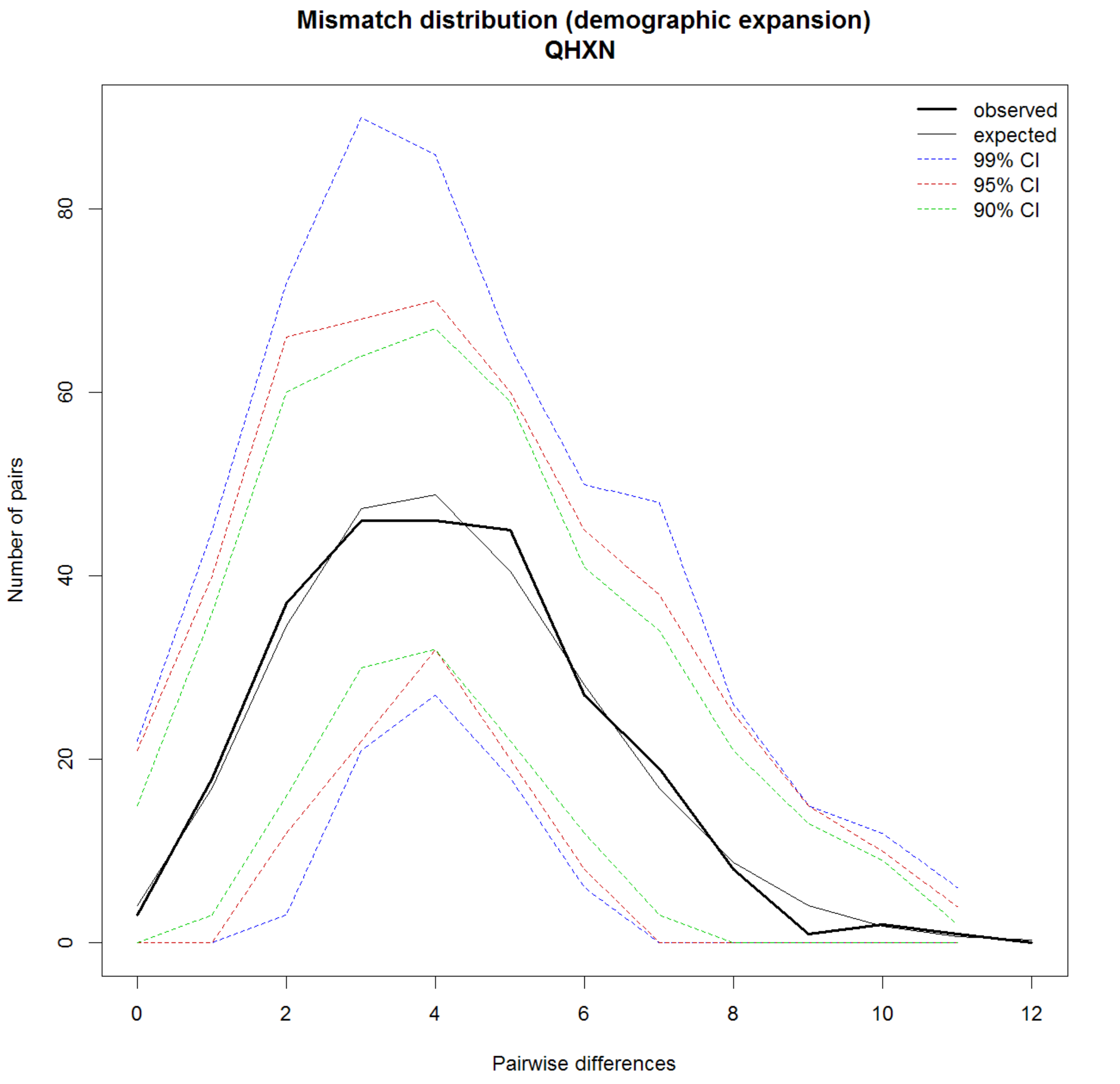
Mismatch distribution (demographic expansion)
QHXN
observed
expected
99% CI
95% CI
90% CI
Number of pairs
0
20
40
60
80
Pairwise differences
0
2
4
6
8
10
12

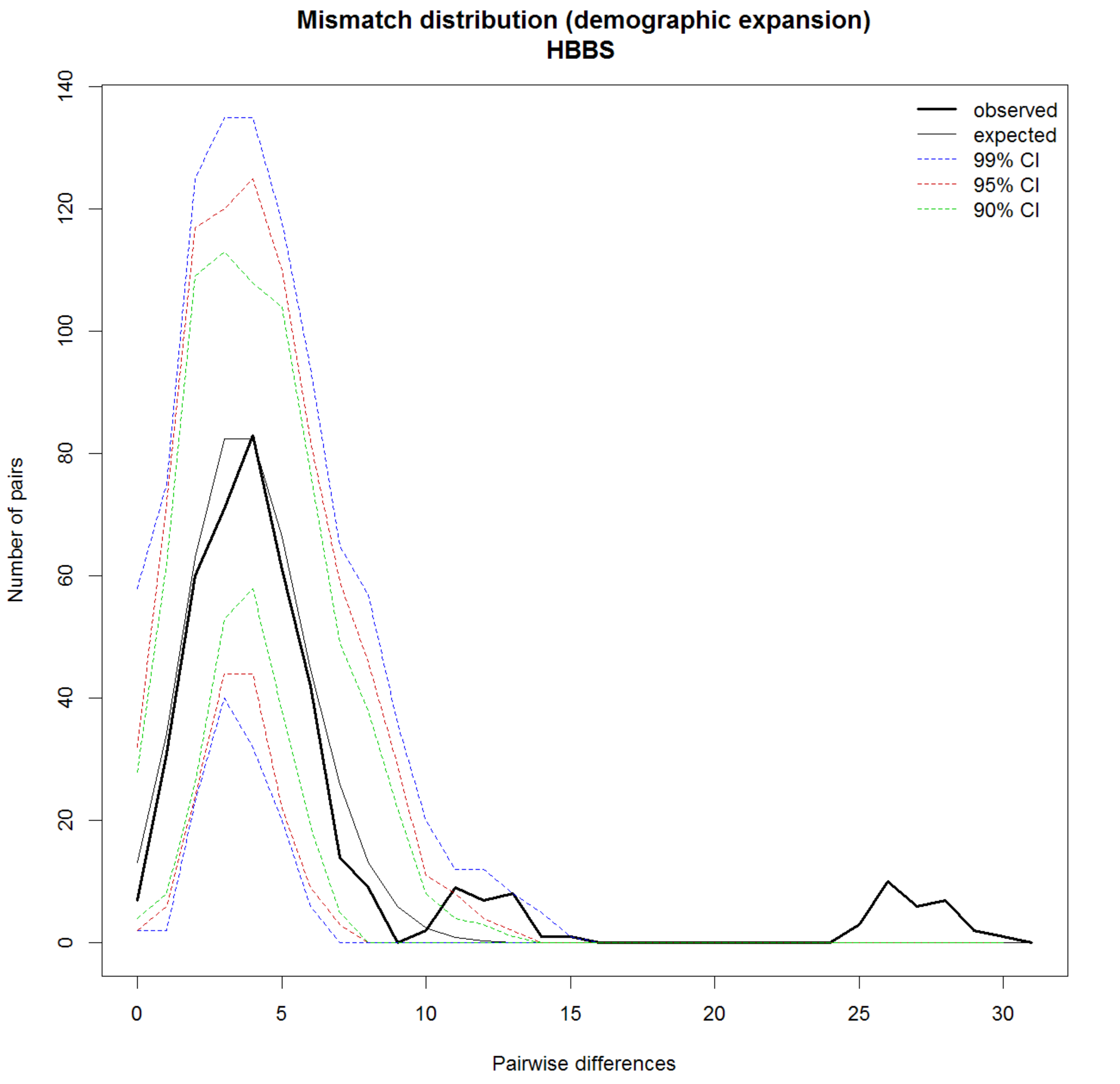

Mismatch distribution (demographic expansion)
HBBS
observed
expected
99% CI
95% CI
90% CI
Number of pairs
Pairwise differences
0
20
40
60
80
100
120
140
0
5
10
15
20
25
30

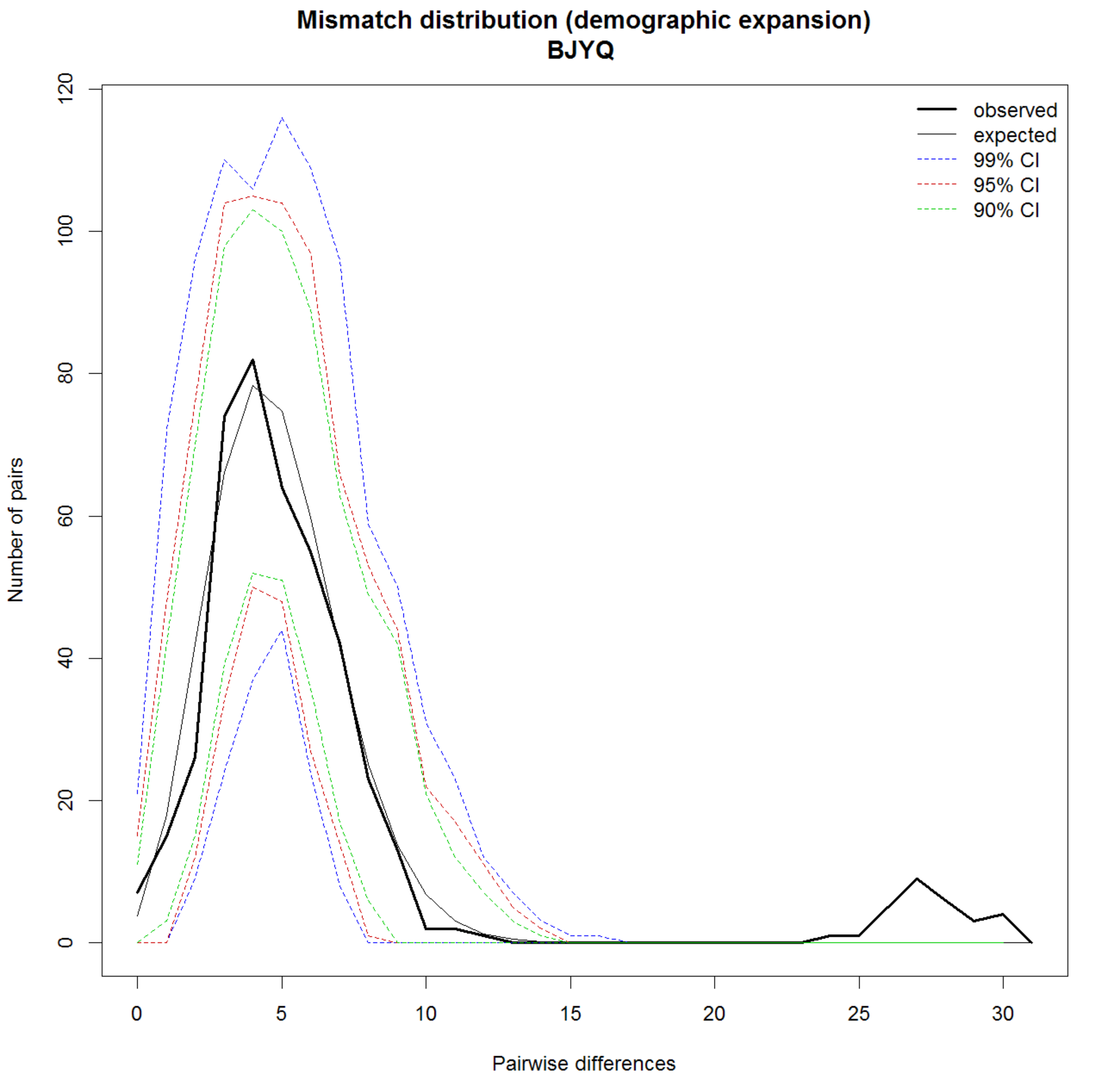
Mismatch distribution (demographic expansion)
BJYQ
observed
expected
99% CI
95% CI
90% CI
Number of pairs
Pairwise differences
0
20
40
60
80
100
120
0
5
10
15
20
25
30

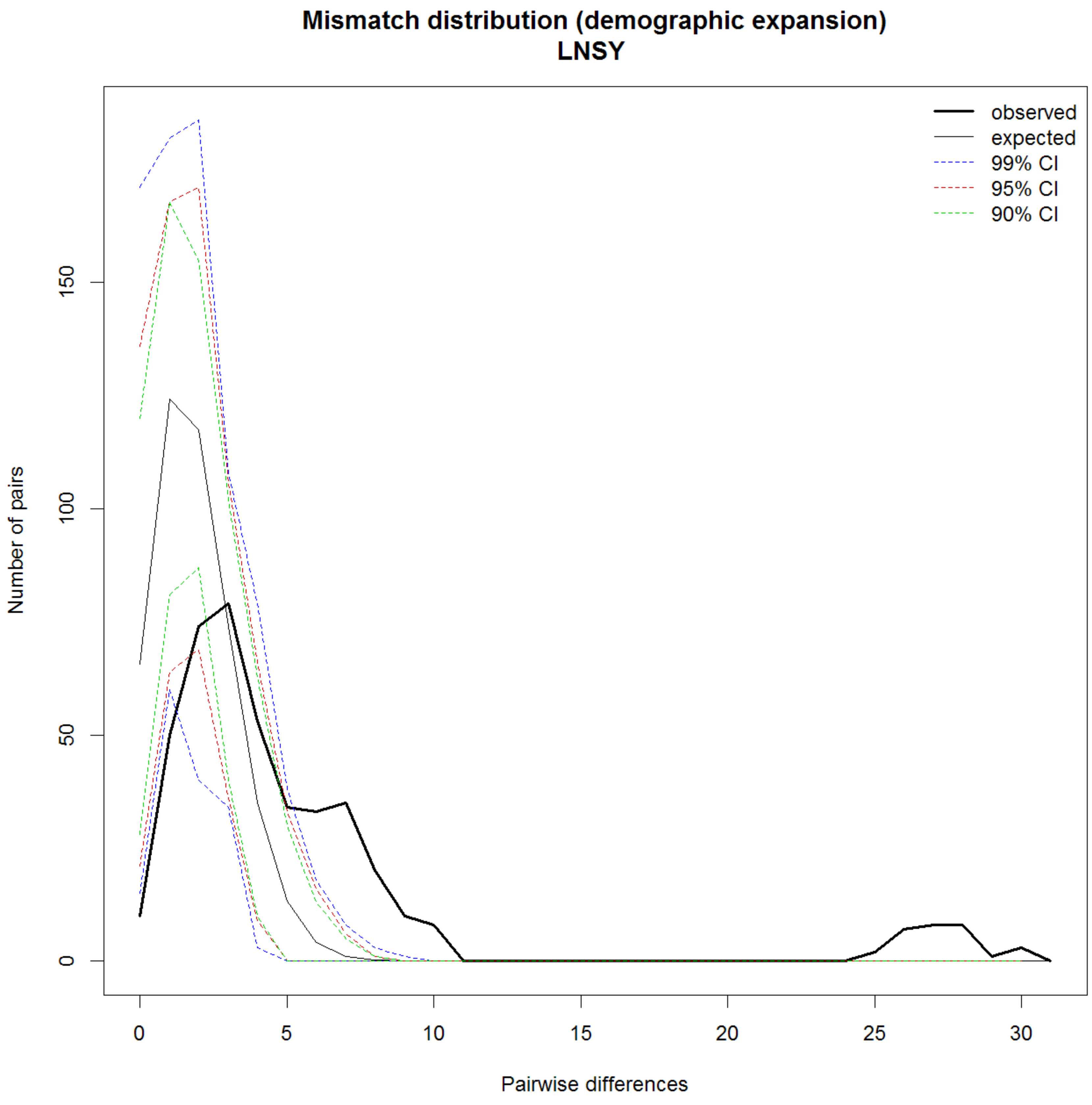

Mismatch distribution (demographic expansion)
LNSY
observed
expected
99% CI
95% CI
90% CI
Number of pairs
0
50
100
150
Pairwise differences
0
5
10
15
20
25
30

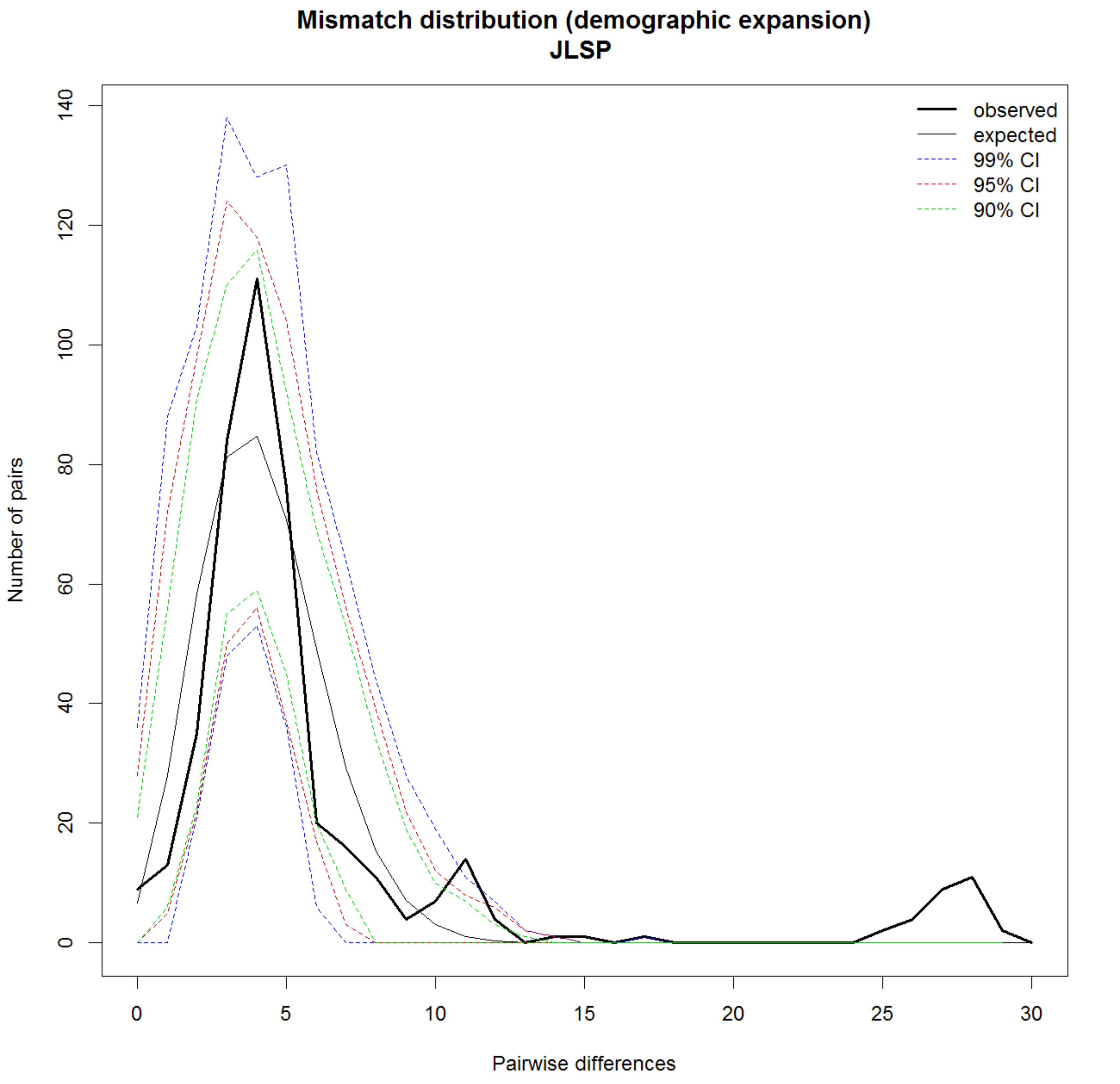

Mismatch distribution (demographic expansion)
JLSP
observed
expected
99% CI
95% CI
90% CI
Number of pairs
Pairwise differences
0
20
40
60
80
100
120
140
0
5
10
15
20
25
30

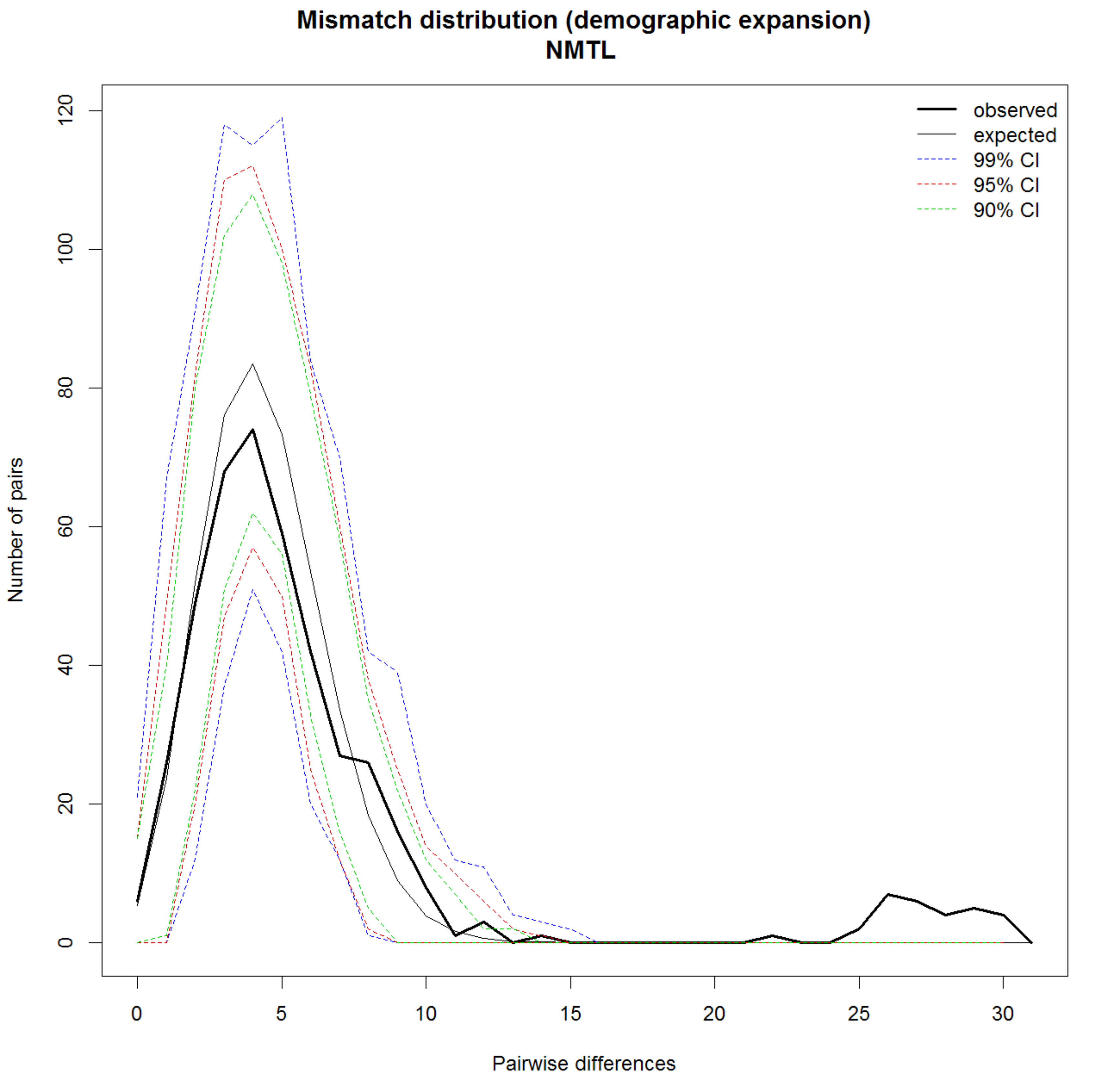
Mismatch distribution (demographic expansion)
NMTL
Number of pairs
Pairwise differences
0
20
40
60
80
100
120
0
5
10
15
20
25
30
observed
expected
99% CI
95% CI
90% CI
