# Supplementary material for: Genetic Structure and Demographic History Reveal Migration of the Diamondback Moth Plutella xylostella (Lepidoptera: Plutellidae) from the Southern to Northern Regions of China
Source: PLoS One. 2013 Apr 2;8(4):e59654. doi: 10.1371/journal.pone.0059654 (PMC3614937; doi:10.1371/journal.pone.0059654)
Supplement: Table S1 — Genetic diversity of the five mitochondrial genes and demographic analysis of the 27 Plutella xylostella populations as one group. (DOCX) [file pone.0059654.s007.docx]

**Table S1** Genetic diversity of five mitochondrial genes and demorgraphic analysis of the 27 *Plutella xylostella* populations as one group

| Gene | H | HI | HP | HS | *S* | *h* | π (%) | *P* | *D* (*p*) | *F_S_* (*p*) |
| --- | --- | --- | --- | --- | --- | --- | --- | --- | --- | --- |
| *cox1* | 205 | 134 | 35 | 36 | 118 | 0.9534 | 0.0039 | 3.2063 | -2.2854 (0.0000) | -25.3403 (0.0010) |
| *trnD* | 2 | 1 | 0 | 1 | 1 | 0.0030 | 0.0000 | 0.0025 | -0.8227 (0.1100) | -3.9936 (0.0020) |
| *atp8* | 12 | 2 | 5 | 5 | 11 | 0.0600 | 0.0004 | 0.0603 | -1.9955 (0.0000) | -27.8641 (0.0000) |
| *atp6* | 60 | 32 | 9 | 19 | 59 | 0.3185 | 0.0011 | 0.5229 | -2.5511 (0.0000) | -28.3818 (0.0000) |
| *nad5* | 93 | 62 | 14 | 17 | 84 | 0.5670 | 0.0019 | 1.2420 | -2.4961 (0.0000) | -27.5340 (0.0000) |

Haplotype number: number of total haplotype (H), number of haplotype unique to individual (HI), number of haplotype unique to population (HP) and number of shared haplotype (HS); Genetic diversity: number of polymorphic sites (*S*), haplotype diversity (*h*), nucleotide diversity (*π*) and average number of pairwise differences (*P*).
