# Supplementary material for: Genetic Structure and Demographic History Reveal Migration of the Diamondback Moth Plutella xylostella (Lepidoptera: Plutellidae) from the Southern to Northern Regions of China
Source: PLoS One. 2013 Apr 2;8(4):e59654. doi: 10.1371/journal.pone.0059654 (PMC3614937; doi:10.1371/journal.pone.0059654)
Supplement: Table S2 — Summary statistics of the nine microsatellite loci examined in the 27 populations of the Plutella xylostella . (DOCX) [file pone.0059654.s008.docx]

**Table S2** Summary statistics of the nine micosatellite loci examined in the 27 population of the diamondback moth

| Population | N | Total no. of alleles | Observed heterozygosity | Expected heterozygosity | F_IS_ |
| --- | --- | --- | --- | --- | --- |
| HNSY | 30 | 114 | 0.5000 | 0.8184 | 0.2474 |
| HNDZ | 30 | 128 | 0.6000 | 0.8424 | 0.3488 |
| GDGZ | 30 | 116 | 0.5333 | 0.8159 | 0.2039 |
| GXLZ | 30 | 97 | 0.6667 | 0.7744 | 0.3053 |
| GXBS | 30 | 117 | 0.9000 | 0.7994 | 0.2012 |
| YNQJ | 24 | 97 | 0.3333 | 0.8083 | 0.2827 |
| FJXM | 30 | 92 | 0.5667 | 0.8033 | 0.3074 |
| FJLY | 30 | 110 | 0.5000 | 0.7920 | 0.2785 |
| FJQZ | 30 | 100 | 0.6333 | 0.8326 | 0.1615 |
| JXNC | 30 | 103 | 0.6000 | 0.7954 | 0.2535 |
| ZJJH | 27 | 105 | 0.7037 | 0.8185 | 0.2899 |
| SHSX | 30 | 101 | 0.1667 | 0.7864 | 0.3918 |
| JSNT | 30 | 104 | 0.5333 | 0.8332 | 0.346 |
| JSNJ | 30 | 79 | 0.7000 | 0.7237 | 0.3025 |
| JSYZ | 30 | 114 | 0.6333 | 0.7999 | 0.3278 |
| JSLY | 30 | 77 | 0.3000 | 0.7509 | 0.3627 |
| HNXY | 30 | 109 | 0.7333 | 0.8220 | 0.3052 |
| HNSQ | 30 | 100 | 0.6667 | 0.8120 | 0.3654 |
| SDQD | 30 | 124 | 0.6000 | 0.8286 | 0.3243 |
| SDYT | 30 | 119 | 0.5667 | 0.8245 | 0.33 |
| QHXN | 23 | 102 | 0.4348 | 0.8224 | 0.4004 |
| HBCL | 30 | 106 | 0.7000 | 0.7982 | 0.303 |
| HBBS | 30 | 135 | 0.5000 | 0.8342 | 0.3199 |
| BJYQ | 30 | 121 | 0.1667 | 0.7728 | 0.4243 |
| LNSY | 30 | 116 | 0.5000 | 0.8123 | 0.4114 |
| JLSP | 30 | 108 | 0.6000 | 0.7584 | 0.3151 |
| NMTL | 30 | 117 | 0.5333 | 0.7734 | 0.3574 |
