# Supplementary material for: Genetic Structure and Demographic History Reveal Migration of the Diamondback Moth Plutella xylostella (Lepidoptera: Plutellidae) from the Southern to Northern Regions of China
Source: PLoS One. 2013 Apr 2;8(4):e59654. doi: 10.1371/journal.pone.0059654 (PMC3614937; doi:10.1371/journal.pone.0059654)
Supplement: Table S3 — Estimates ( M and θ) of the migration among 27 Plutella xylostella populations from China based on combined genes. (DOC) [file pone.0059654.s009.doc]

**Table S3 Estimates (*M* and** **ϴ) of the migration between 27 *Plutella xylostella* populations from the mainland China based on combined genes**

| Population, *i* | *ϴi* | HNSY →*i* | HNDZ→*i* | GDGZ→*i* | GXLZ →*i* | GXBS →*i* | YNQJ →*i* | FJXM →*i* | FJLY →*i* | FJQZ →*i* | JXNC →*i* | ZJJH →*i* | SHSX →*i* | JSNT →*i* |
| --- | --- | --- | --- | --- | --- | --- | --- | --- | --- | --- | --- | --- | --- | --- |
| HNSY | 0.00210 | - | 502.4 | 554.9 | 444.0 | 541.2 | 466.5 | 520.9 | 471.3 | 601.4 | 450.4 | 553.9 | 453.3 | 405.7 |
| HNDZ | 0.03140 | 523.0 | - | 462.8 | 542.7 | 468.5 | 453.0 | 504.4 | 444.2 | 531.8 | 494.6 | 429.0 | 406.3 | 414.1 |
| GDGZ | 0.00183 | 467.8 | 384.1 | - | 496.9 | 455.7 | 388.5 | 415.8 | 436.0 | 508.2 | 553.8 | 415.8 | 418.4 | 393.0 |
| GXLZ | 0.00049 | 420.7 | 497.7 | 380.9 | - | 412.0 | 428.4 | 452.1 | 458.2 | 517.2 | 447.2 | 453.1 | 388.3 | 455.3 |
| GXBS | 0.03328 | 451.0 | 485.4 | 470.5 | 585.1 | - | 567.3 | 470.1 | 483.8 | 492.0 | 460.5 | 449.6 | 479.0 | 424.4 |
| YNQJ | 0.00195 | 463.6 | 443.3 | 440.4 | 522.1 | 506.1 | - | 531.8 | 456.6 | 544.9 | 422.3 | 493.7 | 453.6 | 554.9 |
| FJXM | 0.00067 | 404.0 | 418.9 | 458.6 | 449.8 | 437.2 | 405.8 | - | 444.3 | 558.1 | 502.0 | 465.9 | 437.0 | 474.8 |
| FJLY | 0.00215 | 493.4 | 484.2 | 465.2 | 472.2 | 564.8 | 516.7 | 455.2 | - | 583.1 | 564.7 | 491.1 | 466.3 | 526.7 |
| FJQZ | 0.00041 | 391.1 | 422.8 | 420.8 | 507.7 | 418.4 | 415.1 | 464.1 | 455.3 | - | 477.3 | 432.0 | 398.5 | 494.1 |
| JXNC | 0.00358 | 481.1 | 457.1 | 628.6 | 489.2 | 572.0 | 513.3 | 497.8 | 512.4 | 601.9 | - | 420.8 | 529.6 | 445.0 |
| ZJJH | 0.00861 | 537.0 | 515.3 | 393.3 | 530.8 | 548.8 | 417.5 | 589.2 | 459.7 | 568.2 | 531.8 | - | 620.3 | 446.5 |
| SHSX | 0.00767 | 504.7 | 474.5 | 496.8 | 450.8 | 510.2 | 462.3 | 460.9 | 530.0 | 551.4 | 482.7 | 603.3 | - | 470.2 |
| JSNT | 0.00377 | 517.2 | 437.1 | 460.1 | 518.3 | 389.1 | 522.5 | 511.8 | 525.3 | 613.5 | 485.5 | 461.2 | 501.2 | - |
| JSNJ | 0.00072 | 452.7 | 475.9 | 423.6 | 528.6 | 502.6 | 458.9 | 479.1 | 465.2 | 533.8 | 520.5 | 458.1 | 435.4 | 524.3 |
| JSYZ | 0.01236 | 496.8 | 512.0 | 588.2 | 514.8 | 468.3 | 473.7 | 462.0 | 473.1 | 491.0 | 502.5 | 415.7 | 480.4 | 434.4 |
| JSLY | 0.00025 | 479.1 | 400.7 | 397.3 | 441.5 | 399.1 | 409.5 | 412.0 | 437.9 | 457.1 | 437.7 | 459.8 | 405.4 | 449.6 |
| HNXY | 0.01196 | 447.4 | 454.0 | 444.5 | 440.6 | 504.8 | 438.3 | 481.6 | 608.2 | 503.7 | 443.1 | 423.2 | 513.8 | 527.6 |
| HNSQ | 0.00239 | 454.9 | 460.2 | 437.3 | 521.1 | 445.7 | 468.2 | 492.5 | 392.9 | 568.3 | 505.9 | 442.3 | 443.6 | 511.8 |
| SDQD | 0.06237 | 502.7 | 548.3 | 434.1 | 446.2 | 525.2 | 477.5 | 480.9 | 494.2 | 559.5 | 461.8 | 526.9 | 508.4 | 528.1 |
| SDYT | 0.06377 | 518.6 | 567.4 | 607.2 | 538.0 | 580.3 | 550.3 | 497.2 | 477.5 | 584.4 | 456.9 | 559.2 | 426.2 | 450.1 |
| QHXN | 0.05435 | 453.4 | 467.4 | 524.9 | 534.4 | 547.4 | 455.3 | 543.5 | 402.1 | 571.8 | 600.3 | 533.7 | 456.3 | 512.8 |
| HBCL | 0.01117 | 527.8 | 500.7 | 485.6 | 503.5 | 513.3 | 405.6 | 434.9 | 505.5 | 530.7 | 467.2 | 449.5 | 452.9 | 396.7 |
| HBBS | 0.06404 | 466.3 | 556.8 | 479.5 | 473.3 | 502.6 | 395.2 | 483.6 | 408.7 | 506.9 | 535.1 | 424.9 | 465.9 | 480.9 |
| BJYQ | 0.06248 | 541.9 | 420.7 | 372.0 | 513.9 | 479.2 | 578.4 | 460.4 | 447.3 | 521.0 | 474.5 | 412.1 | 436.0 | 508.0 |
| LNSY | 0.05803 | 418.8 | 430.1 | 396.5 | 480.1 | 485.4 | 518.0 | 388.6 | 428.6 | 662.8 | 589.5 | 538.4 | 453.7 | 592.0 |
| JLSP | 0.02269 | 487.7 | 518.6 | 483.1 | 488.3 | 488.7 | 506.0 | 457.1 | 421.0 | 547.2 | 497.9 | 515.1 | 405.0 | 523.9 |
| NMTL | 0.06468 | 536.2 | 589.6 | 436.1 | 513.2 | 438.2 | 446.1 | 419.3 | 481.5 | 501.2 | 434.7 | 513.9 | 491.4 | 414.8 |

| Population, *i* | JSNJ →*i* | JSYZ →*i* | JSLY →*i* | HNXY →*i* | HNSQ →*i* | SDQD →*i* | SDYT →*i* | QHXN →*i* | HBCL →*i* | HBBS →*i* | BJYQ →*i* | LNSY →*i* | JLSP →*i* | NMTL →*i* | Total *i* |
| --- | --- | --- | --- | --- | --- | --- | --- | --- | --- | --- | --- | --- | --- | --- | --- |
| HNSY | 425.9 | 520.0 | 548.9 | 456.3 | 431.7 | 494.6 | 480.8 | 569.8 | 506.5 | 514.2 | 400.5 | 537.6 | 490.0 | 429.8 | 12772.5 |
| HNDZ | 524.5 | 476.7 | 512.4 | 424.3 | 500.7 | 415.5 | 414.5 | 500.6 | 440.1 | 498.0 | 540.4 | 591.0 | 552.6 | 450.6 | 12516.3 |
| GDGZ | 419.8 | 456.5 | 491.0 | 463.4 | 446.9 | 489.1 | 450.5 | 414.7 | 437.6 | 437.0 | 438.0 | 436.5 | 518.3 | 422.3 | 11655.6 |
| GXLZ | 463.8 | 433.3 | 546.8 | 414.7 | 459.2 | 455.7 | 424.0 | 453.2 | 387.1 | 428.3 | 445.2 | 405.6 | 400.5 | 490.0 | 11518.5 |
| GXBS | 490.1 | 471.4 | 447.7 | 561.2 | 419.6 | 424.8 | 510.1 | 506.1 | 497.0 | 530.6 | 508.6 | 489.3 | 506.7 | 511.7 | 12693.6 |
| YNQJ | 403.5 | 524.4 | 551.0 | 485.3 | 418.3 | 531.0 | 563.0 | 565.3 | 472.3 | 500.7 | 465.5 | 486.9 | 450.6 | 485.2 | 12736.3 |
| FJXM | 423.0 | 399.9 | 505.2 | 409.3 | 468.4 | 442.9 | 531.6 | 464.4 | 477.2 | 410.9 | 460.5 | 503.5 | 440.2 | 490.8 | 11884.2 |
| FJLY | 373.4 | 524.0 | 566.5 | 403.1 | 445.7 | 488.5 | 484.0 | 533.9 | 517.2 | 515.5 | 451.8 | 447.8 | 428.4 | 502.7 | 12766.1 |
| FJQZ | 372.1 | 392.4 | 529.9 | 357.5 | 394.5 | 388.0 | 513.4 | 331.7 | 390.8 | 372.9 | 439.4 | 409.1 | 454.3 | 467.0 | 11110.2 |
| JXNC | 497.1 | 452.5 | 521.3 | 438.1 | 479.6 | 467.6 | 507.9 | 510.0 | 559.9 | 467.7 | 576.7 | 492.2 | 410.6 | 526.0 | 13056.0 |
| ZJJH | 456.7 | 444.3 | 549.0 | 470.4 | 483.9 | 457.5 | 473.2 | 495.0 | 516.4 | 425.5 | 562.9 | 452.5 | 509.4 | 465.2 | 12920.3 |
| SHSX | 535.9 | 445.8 | 469.7 | 495.4 | 438.7 | 507.0 | 517.2 | 512.5 | 488.7 | 506.8 | 451.8 | 506.9 | 498.0 | 475.7 | 12847.9 |
| JSNT | 440.0 | 444.1 | 511.9 | 440.3 | 481.0 | 484.2 | 462.9 | 540.8 | 492.8 | 444.8 | 448.1 | 436.9 | 558.9 | 491.3 | 12620.8 |
| JSNJ | - | 472.5 | 502.0 | 493.8 | 476.2 | 519.6 | 424.7 | 488.8 | 434.8 | 515.8 | 456.6 | 412.7 | 512.1 | 526.7 | 12495.0 |
| JSYZ | 611.0 | - | 615.1 | 448.5 | 454.8 | 554.1 | 536.4 | 489.3 | 484.9 | 453.7 | 450.6 | 484.2 | 411.4 | 472.2 | 12779.1 |
| JSLY | 456.1 | 406.0 | - | 455.7 | 415.7 | 434.4 | 468.0 | 441.4 | 380.4 | 403.4 | 426.7 | 489.9 | 421.6 | 466.6 | 11252.6 |
| HNXY | 650.9 | 484.7 | 515.1 | - | 405.1 | 435.5 | 520.4 | 527.3 | 560.8 | 560.2 | 597.9 | 538.4 | 535.9 | 586.1 | 13149.1 |
| HNSQ | 432.8 | 470.8 | 562.8 | 486.0 | - | 433.9 | 523.5 | 472.3 | 464.6 | 465.3 | 511.2 | 547.3 | 510.9 | 453.9 | 12480.0 |
| SDQD | 451.7 | 469.4 | 475.2 | 527.4 | 474.3 | - | 525.3 | 502.8 | 559.0 | 435.3 | 566.6 | 592.8 | 503.6 | 501.4 | 13078.6 |
| SDYT | 513.0 | 465.7 | 481.0 | 523.6 | 466.8 | 573.4 | - | 483.1 | 507.5 | 526.5 | 563.6 | 470.9 | 466.5 | 472.3 | 13327.2 |
| QHXN | 485.3 | 497.5 | 525.6 | 422.4 | 565.2 | 595.5 | 526.1 | - | 436.6 | 424.8 | 485.0 | 619.8 | 535.8 | 437.5 | 13160.4 |
| HBCL | 464.6 | 544.4 | 599.3 | 567.8 | 466.6 | 549.5 | 578.8 | 494.9 | - | 429.8 | 475.1 | 482.4 | 562.9 | 541.0 | 12931.0 |
| HBBS | 444.3 | 480.9 | 571.8 | 482.9 | 405.4 | 562.0 | 536.3 | 431.4 | 506.4 | - | 502.7 | 532.1 | 419.4 | 570.2 | 12625.5 |
| BJYQ | 571.2 | 401.2 | 472.8 | 493.9 | 494.9 | 474.9 | 481.8 | 497.8 | 553.6 | 484.2 | - | 561.9 | 485.1 | 477.7 | 12616.4 |
| LNSY | 539.0 | 471.0 | 553.4 | 588.4 | 502.9 | 549.1 | 469.9 | 473.8 | 441.1 | 541.9 | 449.3 | - | 546.1 | 495.9 | 13004.3 |
| JLSP | 437.1 | 470.7 | 498.0 | 470.6 | 494.9 | 536.7 | 511.6 | 477.0 | 505.6 | 570.1 | 434.0 | 455.3 | - | 483.2 | 12684.4 |
| NMTL | 443.4 | 584.6 | 603.0 | 535.9 | 522.9 | 421.9 | 400.5 | 507.3 | 469.7 | 505.6 | 558.5 | 515.3 | 463.9 | - | 12748.7 |

ϴ: mutation-scaled population size, which is effective population size × mutation rate per site per generation; *M*: mutation-scaled immigration rate, which is the immigration rate divided by the mutation rate
