# Supplementary material for: Genetic Structure and Demographic History Reveal Migration of the Diamondback Moth Plutella xylostella (Lepidoptera: Plutellidae) from the Southern to Northern Regions of China
Source: PLoS One. 2013 Apr 2;8(4):e59654. doi: 10.1371/journal.pone.0059654 (PMC3614937; doi:10.1371/journal.pone.0059654)
Supplement: Table S4 — The number of the dominant haplotypes in the 27 populations. Hap4, Hap31 and Hap8 are the three most dominant haplotypes in the combined genes, and Hap4, Hap2 and Hap2 are the most dominant haplotypes in the genes cox1, atp6 and nad5, respectively. (DOCX) [file pone.0059654.s010.docx]

**Table S4** The number of the dominant haplotypes in the 27 populations

| Population | Combined gene | | | *cox1* | *atp6* | *nad5* |
| --- | --- | --- | --- | --- | --- | --- |
|  | Hap4 | Hap31 | Hap8 |  |  |  |
| **HNSY** | 0 | 3 | 0 | 2 | 24 | 16 |
| **HNDZ** | 3 | 1 | 0 | 3 | 23 | 15 |
| **GDGZ** | 1 | 0 | 0 | 1 | 27 | 19 |
| **GXLZ** | 9 | 0 | 1 | 10 | 14 | 28 |
| **GXBS** | 2 | 1 | 1 | 4 | 19 | 13 |
| **YNQJ** | 0 | 0 | 5 | 1 | 20 | 19 |
| **FJXM** | 0 | 10 | 0 | 2 | 28 | 16 |
| **FJLY** | 0 | 1 | 1 | 1 | 28 | 21 |
| **FJQZ** | 2 | 14 | 2 | 2 | 30 | 30 |
| JXNC | 2 | 9 | 0 | 5 | 26 | 20 |
| ZJJH | 1 | 3 | 5 | 6 | 27 | 20 |
| SHSX | 0 | 2 | 1 | 0 | 26 | 16 |
| JSNT | 3 | 0 | 1 | 5 | 23 | 28 |
| JSNJ | 0 | 0 | 2 | 0 | 24 | 28 |
| JSYZ | 10 | 0 | 0 | 10 | 28 | 26 |
| JSLY | 2 | 0 | 1 | 4 | 27 | 9 |
| HNXY | 3 | 0 | 0 | 5 | 25 | 19 |
| HNSQ | 4 | 0 | 11 | 6 | 26 | 22 |
| **SDQD** | 2 | 2 | 1 | 3 | 24 | 18 |
| **SDYT** | 3 | 1 | 3 | 6 | 25 | 22 |
| **QHXN** | 1 | 2 | 2 | 6 | 19 | 13 |
| **HBCL** | 4 | 0 | 0 | 9 | 27 | 16 |
| **HBBS** | 5 | 1 | 1 | 5 | 26 | 15 |
| **BJYQ** | 1 | 0 | 4 | 2 | 23 | 19 |
| **LNSY** | 4 | 1 | 3 | 7 | 20 | 19 |
| **JLSP** | 0 | 1 | 2 | 5 | 23 | 16 |
| **NMTL** | 4 | 0 | 1 | 4 | 23 | 17 |
| Total | 66 | 52 | 48 | 114 | 655 | 520 |

The Hap4, Hap31 and Hap8 are the three most dominant haplotypes in the combined gene, and Hap4, Hap2 and Hap2 are the most dominant haplotype in the gene *cox1*, *atp6* and *nad5*, respectively.
