# Supplementary material for: Genetic Structure and Demographic History Reveal Migration of the Diamondback Moth Plutella xylostella (Lepidoptera: Plutellidae) from the Southern to Northern Regions of China
Source: PLoS One. 2013 Apr 2;8(4):e59654. doi: 10.1371/journal.pone.0059654 (PMC3614937; doi:10.1371/journal.pone.0059654)
Supplement: Table S5 — Mitochondrial genes and their amplification conditions used in this study. (DOCX) [file pone.0059654.s011.docx]

**Table S5** Mitochondrial genes and their amplification conditions used in this study

| Gene | Length (bp) | Primer sequence | Tm ( C) |
| --- | --- | --- | --- |
| *cox1* | 870/825 | TACAATTTACCGCTTAAACTC (Forward);  GCTCGGGTATCAATATCTATA (Reverse) | 54 |
|  |  |  |  |
| *trnD* | 66/66 | CATTAGATGACTGAAAGCAAGTA (Forward);  ATGTCCTGCAATTATATTAGC (Reverse) | 48 |
| *atp8* | 168/168 |  |  |
| *atp6* | 540/462 |  |  |
| *nad5* | 700/645 | TTATATCCTTAGAATAAAATCC (Forward);  TTAGGTTGAGATGGTTTAGG (Reverse) | 48 |
|  |  |  |  |

*Gene of *trnD*, *atp8* and *atp6* were amplified together on one segment, while *cox1* and *nad5* were on other two separate segments. The amplified length of each gene are shown before the “/”. Ambiguously sequenced regions at the boundary regions of the PCR products were excluded in analysis and the length of each gene used in final data preparation are shown after the “/”.
