# Supplementary material for: Genetic Structure and Demographic History Reveal Migration of the Diamondback Moth Plutella xylostella (Lepidoptera: Plutellidae) from the Southern to Northern Regions of China
Source: PLoS One. 2013 Apr 2;8(4):e59654. doi: 10.1371/journal.pone.0059654 (PMC3614937; doi:10.1371/journal.pone.0059654)
Supplement: Table S6 — Microsatellite loci used in this study. (DOCX) [file pone.0059654.s012.docx]

**Table S6** Microsatellite loci used in this study

| Locus no. | Locus name in Esselink *et al*. (2006)* | Repeat unit | Range (bp) |
| --- | --- | --- | --- |
| Locus1 | DBMPRI02 | TCG | 110-434 |
| Locus2 | DBMPRI03 | CA | 110-378 |
| Locus3 | DBMPRI012 | GTC | 120-354 |
| Locus4 | DBMPRI014 | CGC | 114-273 |
| Locus5 | DBMPRI015 | CCG | 115-208 |
| Locus6 | DBMPRI016 | CCG | 138-231 |
| Locus7 | DBMPRI021 | GT | 152-356 |
| Locus8 | DBMPRI029 | TGA | 99-372 |
| Locus9 | DBMPRI030 | GCA | 130-265 |

*Esselink GD, Den Belder E, Elderson J, Smulders MJM (2006) Isolation and characterization of trinucleotide repeat microsatellite markers for Plutella xylostella L. *Molecular Ecology Notes* **6**, 1246-1248.
